# High-throughput sequencing and morphology perform equally well for benthic monitoring of marine ecosystems

**DOI:** 10.1038/srep13932

**Published:** 2015-09-10

**Authors:** Franck Lejzerowicz, Philippe Esling, Loïc Pillet, Thomas A. Wilding, Kenneth D. Black, Jan Pawlowski

**Affiliations:** 1 Department of Genetics and Evolution, University of Geneva, Switzerland; 2 IRCAM, UMR 9912, Université Pierre et Marie Curie, Paris, France; 3 ADMM UMR 7144, CNRS, Station Biologique de Roscoff, 29682 Roscoff, France; 4 SAMS, Scottish Marine Institute, Oban, Argyll, UK

## Abstract

Environmental diversity surveys are crucial for the bioassessment of anthropogenic impacts on marine ecosystems. Traditional benthic monitoring relying on morphotaxonomic inventories of macrofaunal communities is expensive, time-consuming and expertise-demanding. High-throughput sequencing of environmental DNA barcodes (metabarcoding) offers an alternative to describe biological communities. However, whether the metabarcoding approach meets the quality standards of benthic monitoring remains to be tested. Here, we compared morphological and eDNA/RNA-based inventories of metazoans from samples collected at 10 stations around a fish farm in Scotland, including near-cage and distant zones. For each of 5 replicate samples per station, we sequenced the V4 region of the 18S rRNA gene using the Illumina technology. After filtering, we obtained 841,766 metazoan sequences clustered in 163 Operational Taxonomic Units (OTUs). We assigned the OTUs by combining local BLAST searches with phylogenetic analyses. We calculated two commonly used indices: the Infaunal Trophic Index and the AZTI Marine Biotic Index. We found that the molecular data faithfully reflect the morphology-based indices and provides an equivalent assessment of the impact associated with fish farms activities. We advocate that future benthic monitoring should integrate metabarcoding as a rapid and accurate tool for the evaluation of the quality of marine benthic ecosystems.

Aquaculture is a rapidly growing industry^1^, which impact on marine benthic ecosystems needs to be evaluated quickly and efficiently^2^. This is traditionally done using physico-chemical measurements and the response of benthic biological communities^3^,^4^. The latter approach is referred to as benthic monitoring and consists of making morphotaxonomic inventories of macro-invertebrates from which various indices are calculated^5^. Beyond generic alpha-diversity measures such as the Shannon diversity H’ or species richness S, specific biotic indices have been formalized in order to ascribe samples into environmental quality classes. These indices include the Infaunal Trophic Index (ITI^6^), the AZTI Marine Biotic Index (AMBI^7^), the Norwegian Sensitivity and Quality Indices (NQI1^8^, NSI^9^) or the Enrichment Stage index (ES^10^). Their formulas include taxon- or cohort-specific weights empirically defined from the autecology of macrofaunal species. The rapid development of salmon farming activities led the main producing countries (Norway, Scotland, Canada, New Zealand) to adopt specific regulations using different reference biotic indices: ITI in Scotland, NSI, NQI1 and AMBI in Norway, ES in New Zealand.

The realization of morphotaxonomic inventories involves the morphological identification of numerous sorted specimens, which is extremely time consuming and taxonomic-expertise demanding. As results take typically several months, it is not possible to respond in a timely manner for effective adaptive management. High-throughput sequencing (HTS) of taxonomic markers enriched from environmental DNA (eDNA) (i.e. metabarcoding) offers an alternative to the morphotaxonomy-based biomonitoring^11^,^12^,^13^. This approach has already been used extensively for exploring the microbial and meiofaunal diversity in various environments^14^. It has also been successful for assessing the quality of freshwater environments, based on the HTS of diatoms^15^,^16^ and aquatic insects^17^,^18^. However, only few studies examined the application of metabarcoding to marine ecosystems^19^,^20^,^21^.

The west coast of Scotland is characterized by sheltered sea lochs, which have been exploited by salmon farmers since the 1980s. Salmon farms consist of a series of nets or pens hanging 10–20 m below the sea surface. Fish farms typically consisting of between 4 and 20 pens are located in areas sheltered from severe storms, but exposed to moderate current flows. The pens are usually aligned with the predominant current flow. Fish-faeces, and uneaten fish-feed, fall down through the water column and accumulate on the seabed around the farm, usually in an ellipsoid shape with the major axis occurring in the direction of the main current. The culture of salmon impacts the benthic environment primarily as a consequence of the accumulation of farm-related detritus (uneaten feed, faeces) around the farm. The detritus increases the biological oxygen demand of the sediment and, if this demand is not met, the sediment becomes hypoxic. Sedimentary hypoxia following organic enrichment is typically associated with the replacement of relatively few large, long-lived, burrowing species by numerous small, short-lived opportunistic species^22^,^23^.

Here, we compare the metabarcoding and morphological approaches in their ability to indicate environmental quality gradients occurring around fish farms. The morphotaxonomic approach is usually restricted to benthic macrofaunal taxa whereas the sequencing of eDNA/RNA molecules can extend the taxonomic analysis by including the meiofauna. From both morphotaxonomic inventories and normalized eDNA/RNA sequence data, we reconstruct both the ITI and AMBI indices for 10 stations located at different distances from salmon cages. We compared the indices inferred from molecular and morphotaxonomic diversity datasets and evaluate how these two different views on the benthic communities impact the assessment of the quality of environmental samples.

## Material and Methods

### Sampling

A total of 10 macrobenthic stations were sampled (Supplementary Table 1). Station 1–9 were distributed along a transect (bearing 240°), extending 400 m from the most southerly (cage centre: −5.500, 56.502, decimal °, WGS84) of 9 circular salmon cages located on the east side of the Isle of Lismore, on the west coast of Scotland. The samples of stations 1–9 were taken in-line with the cages and with the dominant current-flow. Station 10 was situated perpendicularly to the other samples (bearing 135°) but since the dispersion of detritus around cages is elliptical along the water currents axis, it was treated as distant station.

At each station, one macrobenthic sample was collected using a Van-veen grab, from which the redox was measured, five sediment replicates were subsampled for metabarcoding, and the remaining sediment (i.e. the 2 first centimetres over a 0.1 m^2^ area) was treated for morphotaxonomic inventory. The location of each grab was recorded by noting the position of the boat’s A-frame (via a dedicated A-frame mounted dGPS aerial) from which the grab was lowered vertically to the seabed. The position was noted as soon as the grab reached the bottom (as indicated by a slackening of the winch wire), the survey vessel regaining its position (if necessary) prior to recovery. The distance to the fish-cage was determined using the boat’s radar.

Redox was measured immediately following collection using a redox probe (Model CMPtr 106/300 mm; Russel pH Ltd, Auchtermuchty, UK). Prior to use, the probe was checked against a standard solution^24^. The probe was inserted 10 mm into the sediment and the redox value recorded once the reading had stabilized (generally after two to three minutes). Whilst the redox was being measured, the five sediment replicates were sub-sampled from the top 2 cm of the grab using disposable spatulas and immersed into 6 ml of LifeGuard Preservation Solution (MoBio), in order to preserve labile RNA molecules. Once sub-sampling had been completed the sediment was washed through a 1 mm sieve and the residue fixed in 4% borax-buffered formaldehyde prior to macrobenthic sorting and counting. The sieve-retained fauna were identified to species level under the National Marine Biological Quality Control Scheme (NMBAQCS)^25^ by Myriad Taxonomy (Campbeltown, Argyll).

### Molecular analyses

We extracted the total environmental RNA and DNA content of each of the fifty sub-samples using the PowerSoil RNA kit in combination with the DNA Elution Accessory kit (MoBio), according to the manufacturer instructions. The RNA molecules were treated to remove carried-over DNA contaminants and reverse-transcribed to obtain complementary DNA (cDNA) as previously^21^. Then, we enriched the 50 DNA and 50 cDNA extracts for the V4 region of the SSU rRNA gene by PCR amplification. The PCR were realized with the eukaryotic primers pair TAReuk454FWD1 (5′ – CCAGCASCYGCGGTAATTCC – 3′) and TAReukREV3 (5′ – ACTTTCGTTCTTGATYRA – 3′) according to previously published thermo-cycling conditions^26^ and PCR reactors^21^. We used tagged PCR primers to label and multiplex PCR products in one HTS library (Supplementary Table 2) following previous primer design and workflow^21^ and according to a desaturated Latin Square Design in order to reduce the impact of sequence-to-sample misidentifications^27^. We then quantified the amount of amplicons generated by each reaction using relative gel electrophoresis band intensities in order to pool the PCR products in equimolar quantities. We prepared one HTS library from the pool of PCR products according to the instructions of the TruSeq Nano DNA LT Sample Prep kit (Illumina). We then sequenced the resulting library on a MiSeq instrument for 502 cycles (251 cycles paired-end) using the MiSeq Reagent Nano Kit v2.

### Bioinformatics

We quality-filtered and assembled the paired-end reads into full-length sequences following the stringent approach described previously^21^. Then, we performed the de-multiplexing of these sequences into their samples of origin. During the de-multiplexing, we filtered sequence-to-sample misidentifications (i.e. cross-contaminants) due to the mistagging phenomenon as described in the recently published method accounting for unexpected tagged primers^27^. We then dereplicated the filtered set of sequences into Individual Sequence Units (ISUs) and we removed singletons. We considered as singletons every ISU represented by only one read throughout the entire dataset (i.e. we would keep an ISU represented by one read in more than one sample). Then, we extracted all ISUs matching any entry of a subset of the PR2 reference database^28^ containing all Metazoa V4 sequences (23,999 records). We performed BLASTn v. 2.2.25+ searches^29^ as follows: blastn –word_size 20 –max_target_seqs 50 –perc_identity 70 –strand plus. We then used MOTHUR v.1.33.3^30^ to compute pairwise global alignments (Needleman-Wunsch algorithm) and we built Operational Taxonomic Units (OTUs) using a 3% sequence dissimilarity threshold (average linkage clustering). We chose the threshold of 3% in order to avoid inflated diversity estimates, as it has been shown that the 18S rDNA marker reduces the magnitude of diversity estimates, particularly for the meiofauna^31^. We removed the chimeric OTUs originating from the artificial recombination of different sequences by manual inspection of all the candidates identified by Uchime v.4.2^32^ in both “self” and “reference” modes using the following parameters: –abskew 1 –minh 0.3 –xn 5 –minchunk 32.

We then assigned the OTU reference sequences using another round of BLAST searches and phylogenetics. Briefly, we kept the taxonomic consensus of all metazoan reference sequences that best match an OTU sequence along decreasingly stringent combinations of identity (from 100 to 90%) and coverage (100 to 80%) thresholds for all BLAST high scoring pairs (HSPs). If no genus or species could be assigned using HSPs, we used PhyML v.3.0^33^ to build trees from all metazoan reference sequences matching the OTU sequence – but this time along increasingly stringent thresholds – until a supported clade (bootstrap superior or equal to 80/100) containing the OTU sequence was found. In each tree we incorporated three extra sequences belonging to the closest family (or order depending on the taxonomies of the BLAST results) and sharing less than 30% identity with the OTU sequence. For the assignment of the OTU, we kept the taxonomy shared among all the reference sequences constituting the supported clade, but only if we obtained a more precise taxonomy than the taxonomy obtained after the BLAST search.

### Biotic indices

For both morphological and molecular data (but separately for DNA and RNA), we calculated the Infaunal Trophic Index (ITI) as well as three alternative versions of the AMBI^7^. This allows comparing the performance of using either the sequence abundance (H-AMBI) or the OTU richness (S-AMBI) information^34^, or both as it is commonly calculated from morphotaxonomic data (M-AMBI). The taxon-specific bioindicator values for ITI and AMBI were extracted from previous works^35^,^36^,^37^ and from the AZTI software v.5.0, respectively. Because of the uneven distribution of sequences among the samples, we performed a normalization of the OTU-to-sample dataset prior to the indices computations^38^. Briefly, we randomly subsampled the OTUs of each sample replicate 100 times (with replacement), picking a number of reads corresponding to the median of the number of reads per sample (n = 4102). We kept the average number of reads per OTU and no OTUs represented by less than 1.01 reads.

## Results

### Morphotaxonomic analyses

In total, 18,351 specimens representing 116 taxa (including 98 genera) were sorted from 10 grab samples (Supplementary Table 3). On average (±standard deviation), 17.2 (±6.67) species occur at stations close to the cages (within 60 meters), and 47.25 (±8.6) species occur at remote stations. The number of specimens ranges from 366 to 7083 in AZE stations (st. 1 to 6), and from 217 to 320 in distant stations. Both the Shannon H’ and Pielou J indices indicate a lower diversity close to cages (H’ = 0.77 ± 0.24 and J = 0.27 ± 0.07) as compared to remote stations (H’ = 3.2 ± 0.08 and J = 0.83 ± 0.035). The benthic communities in AZE stations are dominated by the annelids: *Capitella spp*, (76.7 ± 11%), *Tubificoides benedii* (1.66 ± 2.13%) and *Malacoceros fuliginosus* (3.03 ± 2.55%) and unidentified nematods (15.8 ± 9.4%). In distant stations (st. 7 to 10), only five specimens belonging to these taxa could be found. The seven species which dominate in the distant stations belong to diverse phyla, including one gastropod (10.79 ± 4.40%), one bivalve (8.47 ± 2.99%), one Echinoderm (5.03 ± 2.31%) and four annelids orders: Capitellida (4.34 ± 2.41%), Terebellida (8.57 ± 2.40%), Spionida (1.93 ± 0.71%) and Phyllodocida (9.15 ± 1.10%).

### HTS data statistics

We obtained about 4.5 million eukaryotic reads distributed across 100 samples (5 DNA and 5 cDNA replicates at 10 sampling stations), from which a subset of 583,574 and 295,727 sequence reads correspond to Metazoa, for DNA and RNA respectively (Supplementary Table 4). We discarded five DNA samples including four from station 1 and one from station 2 because after filtering they contained no sequence or less than three metazoan sequences, respectively. We found significantly more metazoan sequences in the DNA than in the RNA samples (Friedman rank sum test excluding samples 1 and 2 because of missing sample pairs, p-value = 0.008), as well as in two out of three stations situated far from the cages (stations 7 and 9) (Pairwise Wilcoxon Mann-Whitney tests with FDR correction: p-value = 0.036, Supplementary Fig. 1 and Supplementary Table 5). Similarly, the OTU richness is systematically higher in DNA samples than in RNA samples, irrespective of the threshold used for OTUs clustering (Friedman rank sum test excluding samples 1 and 2 because of missing sample pairs, p-value: 1.54 10^−5^).

**Figure 1:**
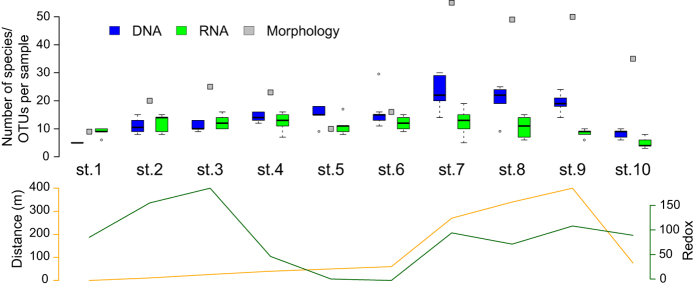
Morphological species and molecular OTU richness. For each station the number of taxa identified morphologically (grey squares) as well as the number of OTUs obtained using DNA (blue boxes) and RNA (green boxes) are shown. The boxplots entail up to 5 samples (PCR replicates) per station for the molecular data. In the lower panel the distance to the cage in meters (yellow) and the redox value (green) for each station are indicated.

We used the sequence dataset of 163 OTUs clustered at 3% dissimilarity for further analyses of metazoan communities. The OTU richness is significantly higher at remote stations than close to the cages, but only for DNA samples (Fig. 1, Supplementary Table 6). For the RNA data, the OTU richness does not show any particular pattern, except for the most distant station 9 appearing less OTU-rich than the stations close to the cages. Interestingly, even in the DNA data the increase of the OTU richness observed in distant stations is much lower than the number of morphologically identified species.

### Taxonomic composition

The molecular assemblage of 163 metazoan OTUs is dominated by annelids (28.2%), Platyhelminthes (20.8%), nematodes (17.8%) and arthropods (14.1%). Other major metazoan phyla, such as molluscs, cnidarians or echinoderms are represented by relatively few OTUs (from 1.22 to 4.29%). Four phyla (Bryozoa, Entoprocta, Priapulida and Sipuncula) occurring in the morphotaxonomic inventory could not be detected in the HTS data. Although represented with reduced OTU diversity, we could detect the presence of Hemichordates and several small-sized phyla (Gastrotricha, Kinorhyncha and Rotifera) that are not reported in the morphotaxonomic study. The most striking discrepancy between morphological and molecular data is that numerous OTUs could be assigned to Platyhelminthes and Acoelomorpha whereas these meiofaunal taxa are not included in the morphotaxonomic inventories (Supplementary Tables 3 and 7).

There are also important differences between morphotaxonomic and metabarcoding analyses at lower taxonomic levels. Although the annelids dominate both assemblages, their richness at the family level is much lower in the HTS data (12 families) compared to the morphological inventory (26 families). This difference is even higher for molluscs, with only 7 genera detected with HTS versus 24 genera with morphological examination. Similarly, none of the 11 Malacostraca species identified morphologically are present in the HTS crustacean assemblage dominated by the copepods and ostracods. Yet, the proportions of the taxa that can be found using both techniques are fairly similar. For instance, out of 11 shared genera, only the proportions of Arthropoda and Mollusca genera are highly skewed (Supplementary Fig. 2). This is also the case for the 15 shared families, that are represented by a majority of the OTUs (60.2%) and species (53.5%) assigned to a family.

In spite of these differences, the congruence between morphological and HTS data is high for the most abundant morphotaxa. The genus *Capitella* by far dominates both morphological and molecular datasets. The next three most abundant morphotaxa (Nemertea, *Malacoceros fuliginous*, *Tubificoides*) represented by more than 200 specimens are also present in the HTS data. Nevertheless, the proportion of taxa present in both morphological and HTS datasets decreases rapidly for the rare ones. In total, less than 20% of morphotaxa are found in the HTS data.

### Metazoan OTUs distribution

**Figure 2:**
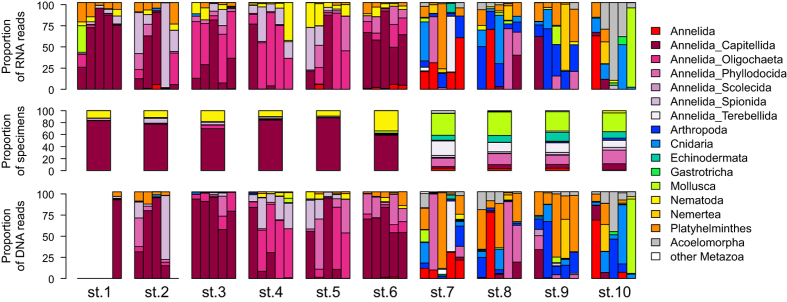
Taxonomic composition. For each of the samples grouped per station the proportions of taxa identified morphologically (middle panel) as well as using RNA (upper panel) and DNA (lower panel) are shown. The proportions of taxa at the phylum level and at the order level for Annelida taxa are shown.

**Figure 3:**
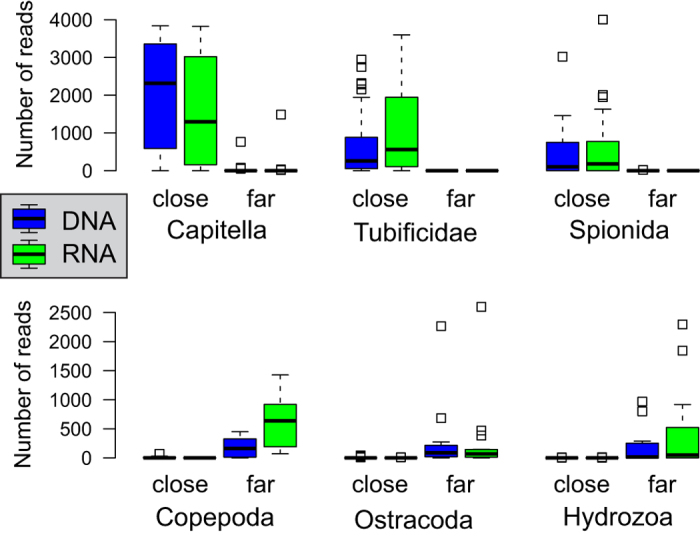
Sequence reads abundances of selected bioindicator taxa binned per class of distance to the cage. The two bins correspond to the stations found at or closer than 60 meters from a cage (close) and to the stations located over 60 meters from a cage (far). The numbers of DNA- (blue boxes) and RNA- (green boxes) derived reads are shown separately.

We analyzed the distribution of OTUs in different stations, separately for DNA and RNA, and compared it to the morphotaxonomic inventories (Fig. 2). The metazoan assemblage in AZE samples collected close to the cages is very different from the distant samples. In the AZE samples the dominant taxa are *Capitella*, Tubificidae, *Malacoceros* (Spionida), an unassigned species of Cirratulidae (OTU6) and the nematodes (Fig. 3). The genus *Capitella* is present in all AZE samples where it accounts for up to 93.7% of sample sequences. However, it is rare in distant samples, with relative sequence abundances usually lower than 2% (excepted in one sample of station 9). The main Tubificidae, Cirratulidae and *Malacoceros* OTUs are also abundantly sequenced in AZE samples, representing up to 87.8%, 48.8% and 97.6% of sample sequences, respectively. Interestingly, the presence of Cirratulidae is restricted from 25 to 60 m off the cage while *Malacoceros* only occur in the samples within 11 m. Both OTUs are absent from distant samples. The nematode OTUs exceptionally reach the abundance of 43.7% in a sample, but in general never exceed 10% in AZE samples (on average 5.04 ± 7.78% SD) and 5% in distant samples.

The distant samples are characterized by highly diversified assemblages compared to the AZE samples. The replicates taken at the same distant station rarely present the same taxonomic composition, as evidenced by sample dissimilarities computed based on the presence/absence of OTUs (Supplementary Fig. 3). When compared along with the distant samples, the AZE samples all seem similar, but the replicates remain more similar when the sequence abundance information is used (especially for DNA). Moreover, there is a good congruence between the sequence abundance observed in DNA and RNA datasets (Fig. 2).

The distant samples are rarely dominated by a single OTU, except the OTU assigned to the bivalve genus *Corbula*, which comprises 91% of one of the replicates of the station 10. The taxonomic groups most commonly sequenced in the distant samples are Copepods, Ostracods, Hydrozoans, Nemerteans, Acoelomorpha and Platyhelminthes (Fig. 3). Some of these groups are totally absent from AZE samples (Acoelomorpha, Nemertea), while others can be found but with relative sequence abundances generally below 1% in a sample. Interestingly, some OTUs can be very abundant in a single distant sample and absent (Ampharetidae OTU21) or moderately common (*Phyllodoce maculata*) in other samples.

The distribution of OTUs matches relatively well the morphotaxonomic inventories. In morphological counts, the AZE samples are dominated by Capitellids (58.5–87.8%) like in the HTS data. The Tubificidae and Spionidae (*Malacoceros*) are also present in both datasets, but their abundance is much higher in the HTS data. In contrast, the nematodes show reverse pattern, being much more abundant in morphological counts (9.1–33.3%) than in the DNA/RNA samples from the AZE stations. This high congruence between morphological and HTS data in AZE stations is clearly less pronounced in the distant samples. The morphological inventories of these samples are largely dominated by molluscs (31.3–39.3%) and ophiuroids (10.7–14.7). Both groups are represented by few OTUs, which relative abundance is generally low (except *Corbula gibba).* Several taxonomic groups represented by the OTUs common in distant stations, such as Hydrozoa or Acoelomorpha, are absent from morphotaxonomic inventories. On the other hand, some abundant OTUs, particularly within annelids, possibly correspond to the sorted morphospecies but could only be identified to the family level.

### Biotic indices

**Figure 4:**
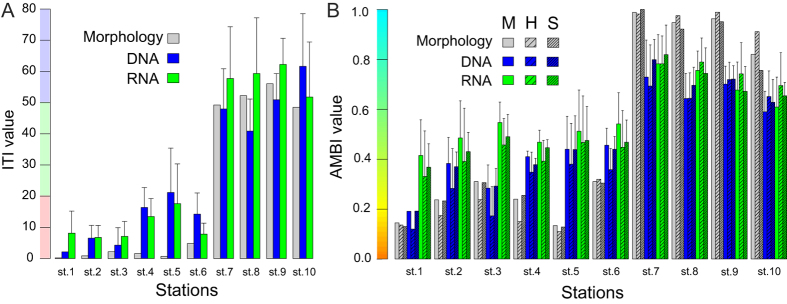
Infaunal Trophic Index (ITI) and AZTI’s Marine Biotic Index (AMBI). (**A**) Only one ITI value could be calculated for the morphologically derived values (grey bars) while the mean value of up to 5 samples PCR replicates is shown for both DNA (blue bars) and RNA (green bars). For the molecular data, the error bars indicate the standard deviation obtained after 1000 ITI calculations based on the OTUs diversity of one randomly picked sample per station out of up to 5 PCR replicate samples sequenced per station. For the AMBI calculations, the error bars indicate the standard deviation across the sample replicates. The coloured background indicates the three discrete environmental quality values corresponding to ITI values. (**B**) For each station is indicated the AMBI values calculated based either on the Shannon diversity (H-AMBI; sparsely hatched bars), the species richness (S-AMBI; densely hatched bars) or both (M-AMBI; plain bars). The ramping coloured background indicates the continuous environmental quality depicted by the AMBI.

The ITI and AMBI (M-AMBI, H-AMBI and S-AMBI) values inferred from the molecular data reflect similar ecological conditions to the corresponding values inferred from the reference morphological data (Fig. 4). The morphology-based ITI values are extremely low for the AZE stations and very high for the distant stations, indicating a clear separation between the strongly impacted conditions (ITI < 20) of the former and the low impact of the fish farms on the benthic communities living father than 300 m from the cages (ITI > 50). The same clear-cut difference between AZE and distant stations are observed in values of AMBI, regardless of whether the index calculation is based on sequence abundance (H-AMBI), OTU richness (S-AMBI) or both (M-AMBI) and of the fact that only half of the sequenced taxa are associated with an AMBI ecological group (Supplementary Fig. 4). Interestingly, the station 10, situated on the other side of the fish farm, at distance of 76 m, shows ITI and AMBI values similar to the distant stations, presumably because this station was oriented perpendicularly to the direction of the residual current, corresponding to the main depositionary axis of the fish farm.

**Figure 5:**
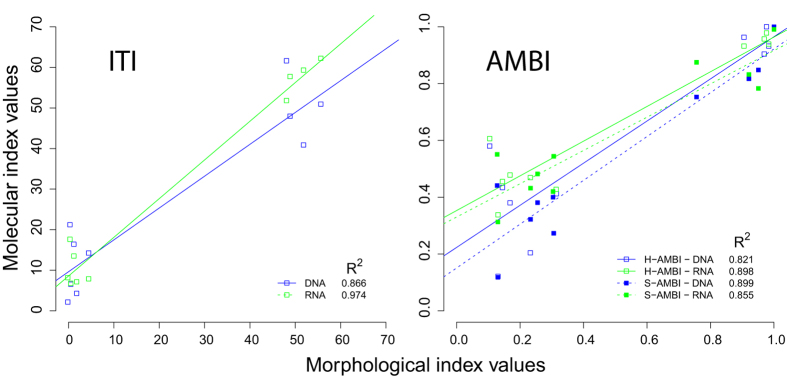
Relationship between biotic index values derived morphological and molecular data. The index values are shown separately for the ITI (left panel) and AMBI (right panel) indices. The relationships are represented for both the H-AMBI and S-AMBI calculations. Linear regressions and associated coefficients of determination are indicated. All correlations are highly significant (p < 0.001).

The correlation between values inferred from morphological and molecular data is very high for both ITI (DNA: R^2^ = 0.866, RNA: R^2^ = 0.974) and AMBI indices (H-AMBI DNA: R^2^ = 0.821, RNA: R^2^ = 0.898; S-AMBI DNA: R^2^ = 0.899, RNA: R^2^ = 0.855; M-AMBI DNA: R^2^ = 0.811, RNA: R^2^ = 0.868) (Fig. 5). In the case of AZE stations, the values of ITI and AMBI indices inferred from DNA/RNA data are higher than those based on morphological analyses. This difference is particularly pronounced in the case of station 5 (50 m off the cage), with ITI, H-AMBI, S-AMBI and M-AMBI values inferred from DNA being 30.8, 3.45, and 3.4 and 3.3 times higher, respectively. In general, the correlation seems better in the case of RNA than DNA. Interestingly, the index values inferred from RNA are higher than those inferred from DNA data in the three closest stations from the cage (1 to 3) as well as in the three distant stations (7 to 9) but only for ITI.

## Discussion

This study confirms the usefulness of metabarcoding to estimate the biotic indices routinely used in benthic monitoring of marine ecosystems. The outcome of the traditional morphotaxonomic approach is similar to the HTS eDNA approach, even though both involve very different sampling volumes and rely on the contrasting diversity of different set of taxa. Our results are promising but need to be interpreted with caution in order to understand the challenges of the new approach and fully appreciate its potential.

Applying metabarcoding for benthic monitoring offers numerous practical advantages. Current developments towards automation and reduction of analytic steps will greatly simplify the use of DNA sequences as species identifiers and accelerate benthic biomonitoring surveys. It will make analysis independent of taxonomic expertise, overcoming the issue of taxonomic impediment and misidentification biases^39^. Moreover, the metabarcoding will allow extending the range of potential bio-indicators to meiofauna^40^,^41^ and protists^21^,^42^.

Compared to the morphological approach, metabarcoding provides a more holistic view of the metazoan taxonomic diversity, regardless of the size and developmental stage. Our HTS data not only include the macrofaunal species that dominate in the morphological samples, but also small-sized (<1 mm) species, extending the scope of analysis to the much broader meiofaunal diversity. In fact, taxa such as harpacticoid copepods, ostracods and many minor groups (gastrotriches, kinorhynchans, rotifers) are currently not included in the morphology-based bioassessments. Yet, some of them (kinorhynchans, turbellarians, ostracods) have been shown to be good indicators for assessing the impact of finfish^43^ and shellfish farming^44^. Hence, metabarcoding might be the only way to account for these meiofaunal-size organisms.

It is not surprising that the metabarcoding data is enriched with meiofauna sequences. Indeed, it is more likely that small rather than large organisms would be captured in 2-grams sediment samples used for molecular analyses. The macrofaunal species sequences may well originate from tissue fragments, mucus, eggs or larvae^45^. However, the majority of macrofaunal DNA likely originates from extracellular DNA. Large quantities of extracellular DNA are preserved in the sediment^46^. This extracellular DNA accumulates over seasons and thus integrates the diversity of several population turnovers, including pioneer species that may constitute the short-time response to environmental perturbation. This is supported by the fact that the DNA-based biotic indices better reflect the sites environmental quality than those based on short-lived RNA molecules. RNA reflects the active fraction of the diversity^47^, and thus might be less efficient than DNA to capture the macrofaunal diversity. It seems that DNA buffers the high natural variability observed between biological replicates^41^. Nevertheless, the presence of extracellular DNA might represents a major strength of the metabarcoding approach as for the detection of macrofaunal taxa, especially annelids that are well represented in our samples. We obtained fewer sequences for other macrofaunal groups, such as molluscs and echinoderms. This may be due to the presence of shells or hard walls, which impede the diffusion of their DNA into the surrounding environment or the absence of mucus and small-sized benthic developmental stages.

Beyond its many advantages and ease of use, the routine application of metabarcoding for benthic monitoring requires overcoming some limitations. The main shortcomings involve the incompleteness of reference sequence databases as well as the fragmented knowledge on meiofaunal autecology. Despite considerable barcoding efforts, reference sequences are still very rare for benthic meiofaunal species. Given the prevalence of meiofauna in molecular assemblages, it is crucial to further describe potential meiofaunal bioindicator taxa not only through their genetic identification but also to specify their ecological values. Indeed, only half of the taxa detected with HTS could be ascribed to an ecological group and a unique ecological value is often assigned to an entire meiofaunal phylum. For instance, the nematodes form a hyper diverse group^48^, but all are ascribed to the same AMBI ecological group. Given the immense phylogenetic diversity of meiofaunal groups, the relevance of these values and thus of the inferred indices is doubtful.

It is also important that molecular databases include more than one gene. At present, most of benthic metazoans are represented either by 18S rRNA gene or COI gene. Although these markers have different advantages and offer different taxonomic resolutions, both suffer similar limitations related to database incompleteness and primer specificities, and ideally should be coupled as in a recent comparative study^49^. We chose the V4 fragment of the 18S because it is shorter and easier to PCR amplify and sequence using Illumina technology. However, the resolution of the V4 region is limited for species-level assignments and has been shown to provide less accurate diversity estimates than COI^31^. Nevertheless, some key species could only be detected using the 18S marker, as *Malacoceros fulginosus,* for which there is no reference COI sequence. With upcoming extensions of Illumina sequencing read lengths, it will be possible to sequence the full COI barcode, which might be more informative for future metazoan-based metabarcoding. Alternatively, improvements are being proposed towards the design of new primers targeting shorter COI fragments^50^ or new amplification strategies^51^. However, the COI marker is a protein-coding gene and thus remains less suitable than the 18S rDNA marker for the design of universal primers^40^. Moreover, the use of COI is hampered by difficulties in assigning higher taxonomic level to those sequences that lack close correspondence in the reference database^52^. Finally, it is necessary to expand the dimension of DNA sequence databases by gathering knowledge on gene copy numbers and polymorphisms. With this new information on intra-genomic variation in hand, it will be possible to refine sequence taxonomic assignments and quantitative ecological inferences.

Another important challenge is to develop biotic indices specifically for HTS data and assign appropriate scores to species given their autecology^53^. The currently used ITI and AMBI formulas have been developed for morphotaxonomic inventories of marine species. In their formulas, the ecological weight of each taxon morphologically isolated and identified in an environmental sample is used as a factor of its abundance in the sample. However, HTS sequence abundance data depend on many technical and biological biases and its exploitation for quantitative analyses remains a major issue. In fact, the relative abundance of DNA template molecules can be obtained if rigorous HTS data filtering is undertaken^27^, and useful relative abundance information could be drawn from analyses performed at coarse taxonomic levels^54^, which is inherent to the use of the 18S marker^31^. In our comparative study, the sequence abundance is not completely disconnected from the abundance of specimens, as shown by the high similarities of sequence proportions for the taxa found with both approaches, as well as among station replicates and in terms of relative abundances of the dominant taxa (e.g. *Capitella*). Such up-weighting of the dominant bioindicator taxa certainly improved the reconstruction of biotic indices. Biotic indices may integrate multiple diversity metrics (M-AMBI^55^). However, it has been shown that relying on the richness only (S-AMBI) or on the Shannon diversity only (H-AMBI) performs equally well and avoids unnecessary statistical noise^34^. Therefore, we recommend the use of the sequence abundance information for biomonitoring purposes, and thus the use of H-AMBI over S-AMBI.

Beyond the use of alpha-diversity metrics, beta-diversity patterns could be incorporated in a HTS index, as recently proposed in the novel index designed for coralligenous macroalgal assemblages^56^. For example, accounting for the dispersion propensity and distance of bioindicator species when studying communities sampled along distance-to-cage gradients remain a major challenge^3^. Further surveys along smoother environmental gradients are needed as our sampling strategy involves a categorical jump from impacted to non-impacted (and therefore high correlations values). In fact, this is crucial for DNA-based studies given the ability of extracellular DNA to be carried over great distances by water currents and turbulences. It is also important to reconsider the HTS data normalization step, as statistical models accounting for sequence abundances heteroscedasticities could replace rarefaction approaches^57^.

To conclude, our study shows that the metabarcoding has potential to revolutionize benthic monitoring surveys. Its implementation will require some efforts, especially concerning the adaptation of biotic indices to molecular data. However, the advantages provided by the standardization and automation of the eDNA-based benthic monitoring fully justifies the further developments of this approach.

## Additional Information

**How to cite this article**: Lejzerowicz, F. *et al.* High-throughput sequencing and morphology perform equally well for benthic monitoring of marine ecosystems. *Sci. Rep.* **5**, 13932; doi: 10.1038/srep13932 (2015).

## Supplementary Material

Supplementary Information
