# Supplementary material for: High-throughput sequencing and morphology perform equally well for benthic monitoring of marine ecosystems
Source: Sci Rep. 2015 Sep 10;5:13932. doi: 10.1038/srep13932 (PMC4564730; doi:10.1038/srep13932)
Supplement: Supplementary Information [file srep13932-s1.pdf]

Supplementary Information:

High-throughput sequencing and morphology perform equally well for benthic monitoring of marine ecosystems

Franck Lejzerowicz[1], Philippe Esling[1,2], Loïc Pillet[1,3], Thomas A. Wilding[4], Kenneth D. Black[4], Jan Pawlowski[1]

[1] Department of Genetics and Evolution, University of Geneva, Switzerland

[2] IRCAM, UMR 9912, Université Pierre et Marie Curie, Paris, France

[3] ADMM UMR 7144, CNRS, Station Biologique de Roscoff, 29682 Roscoff, France

[4] SAMS, Scottish Marine Institute, Oban, Argyll, UK

* To whom correspondence should be addressed. Tel: +41223793077; Fax: +41223793340; Email: franck.lejzerowicz@unige.ch

Present Address: [Franck Lejzerowicz], Department of Genetics and Evolution, University of Geneva, Sciences 3, 30, Quai Ernest Ansermet, CH-1211 Geneva 4, Switzerland

Short title: High-throughput sequencing for benthic monitoring

Supplementary Figure 1. Sequence reads filtering. The log10-transformed numbers of DNA (upper panel) and RNA (lower panel) sequence reads are shown after sequencing (raw), after the quality-filtering[23] and de-multiplexing[29] as (filt) and after selection of Metazoan sequences (meta). Each boxplot represents a maximum of 5 samples for each of the 10 stations

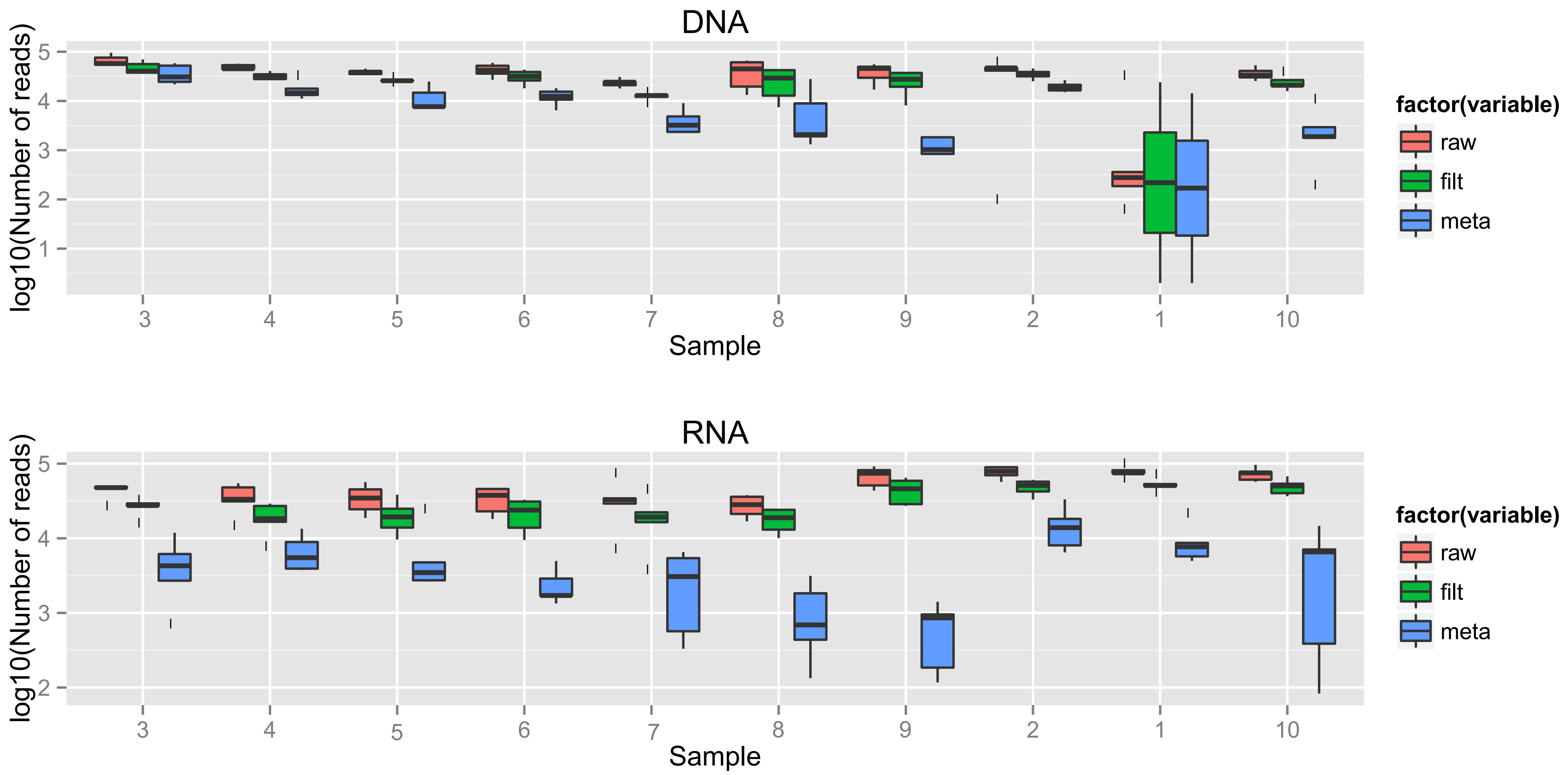
DNA
RNA
log10(Number of reads)
Sample
3
4
5
6
7
8
9
2
1
10
factor(variable)
raw
filt
meta

Supplementary Figure 2: Comparison of the diversity detected by the molecular and morphotaxonomic approaches at each assigned taxonomic level. The taxa to which the OTUs/species could be assigned are either found in both the molecular and morphological dataset (“Both”), in the molecular dataset only (“Molecular”) or in the morphological dataset only (“Morphological”). For each of these fractions, a pie chart represents the proportions of taxa in terms of OTU (for the molecular approach) or species (for the morphotaxonomic approach). For the “Both” fraction, the outer chart represents the molecular data and the inner chart represents the morphotaxonomic data. The numbers inside the circles correspond to the numbers of assigned OTU/species while the numbers outside the circles indicate the number of taxa that could have be assigned the taxonomic level under consideration.

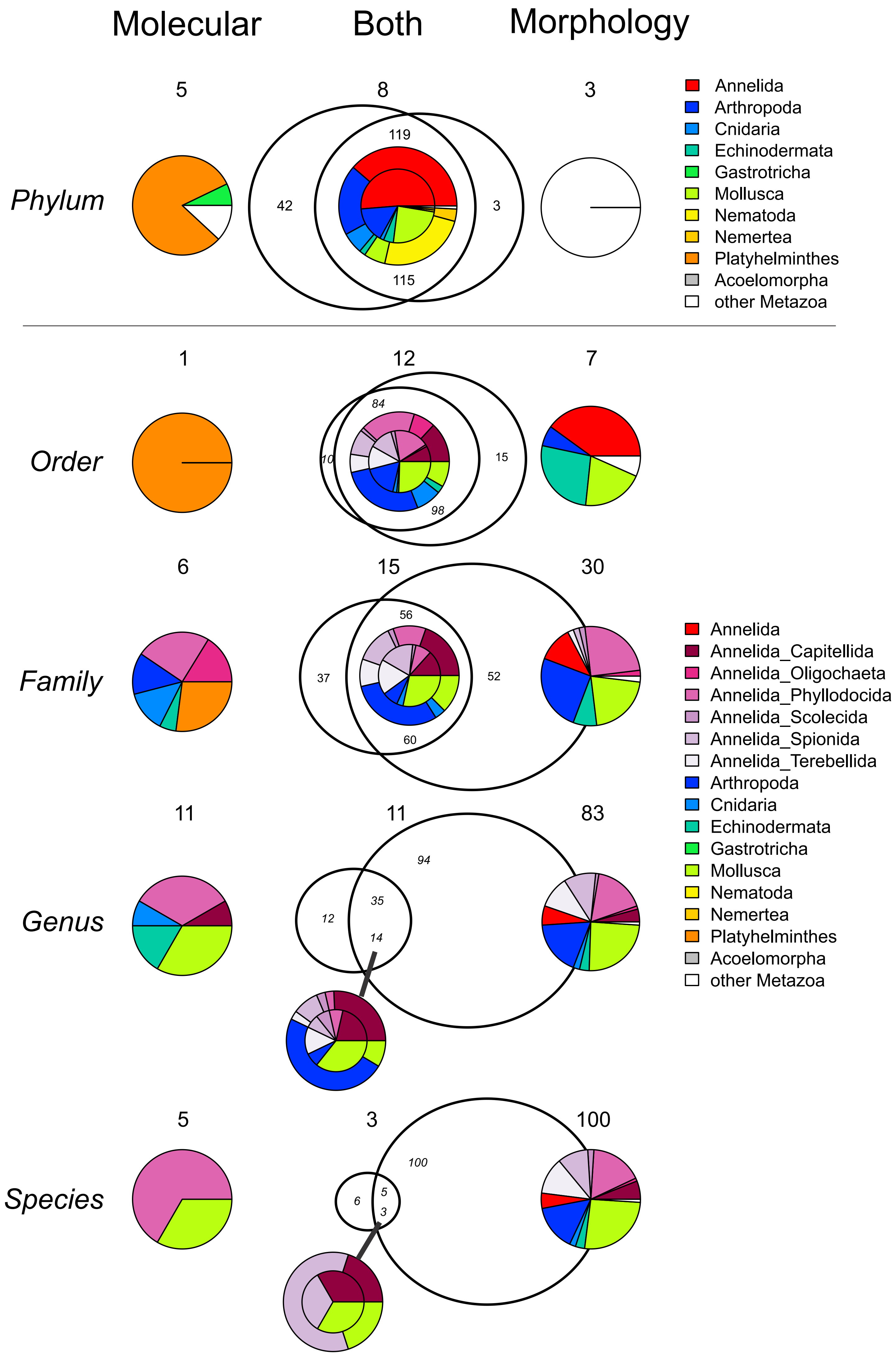

Molecular
Both
Morphology
Phylum
Order
Family
Genus
Species
5
8
3
119
42
3
115
1
12
7
84
10
15
98
6
15
30
56
37
52
60
11
11
83
94
12
35
14
5
3
100
100
6
5
3
Annelida
Arthropoda
Cnidaria
Echinodermata
Gastrotricha
Mollusca
Nematoda
Nemertea
Platyhelminthes
Acoelomorpha
other Metazoa
Annelida
Annelida_Capitellida
Annelida_Oligochaeta
Annelida_Phyllodocida
Annelida_Scolecida
Annelida_Spionida
Annelida_Terebellida
Arthropoda
Cnidaria
Echinodermata
Gastrotricha
Mollusca
Nematoda
Nemertea
Platyhelminthes
Acoelomorpha
other Metazoa

Supplementary Figure 3: Non-metric multidimensional scaling plots. The plots are presented with all the biological replicate samples (a, d), only the DNA replicates of the impacted stations (b, e) or only the RNA replicates of the impacted stations (c, f). All sample distances are computed using the Jaccard dissimilary index and based either on OTU presence/absence data (upper panels) or normalized OTU sequence abundance data (lower panels).

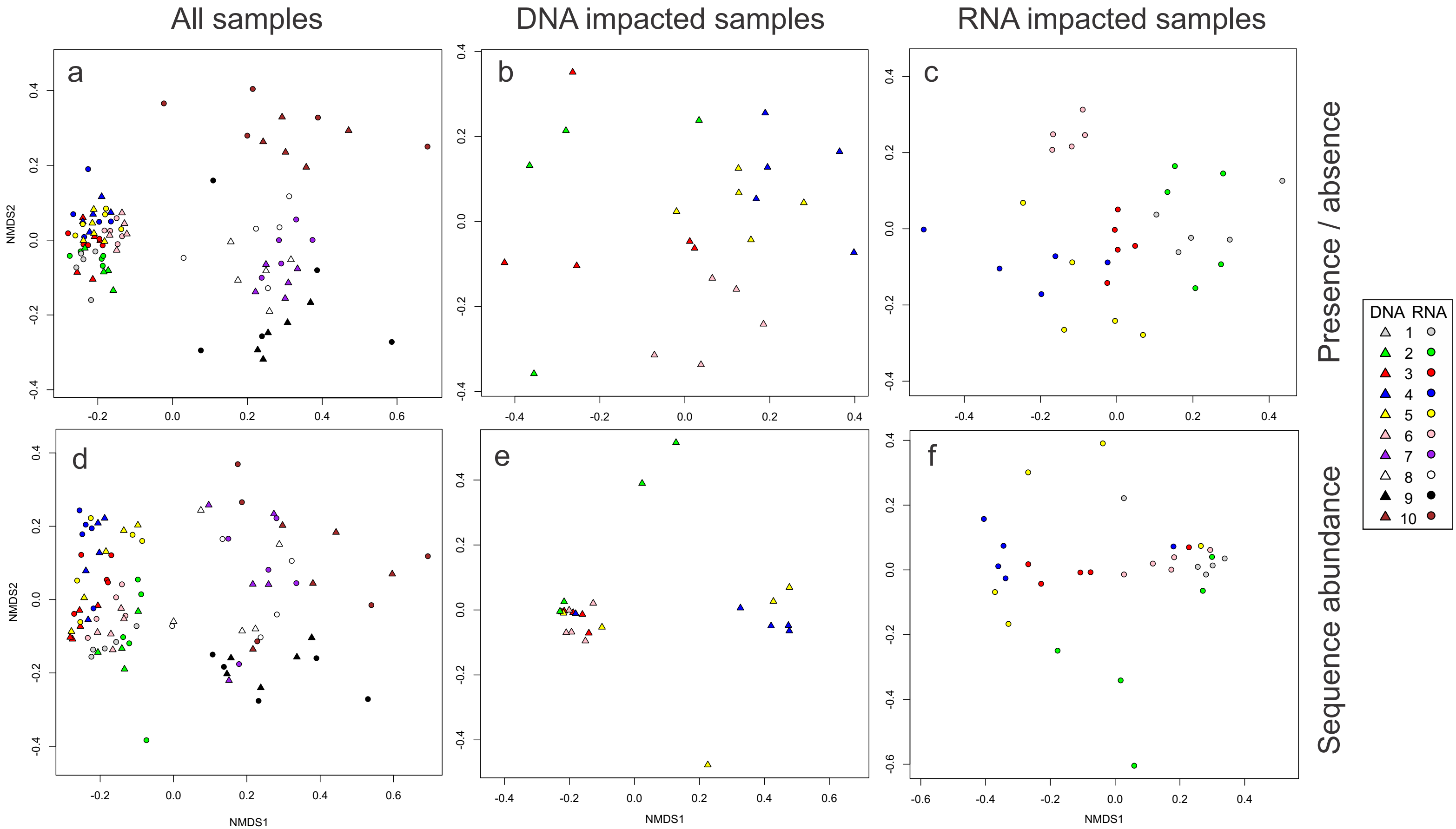

All samples
DNA impacted samples
RNA impacted samples
Presence / absence
Sequence abundance
a
b
c
d
e
f
NMDS1
NMDS2
DNA RNA
1
2
3
4
5
6
7
8
9
10

Supplementary Figure 4: Proportions of taxa associated with the different ecological groups used for the computation of the biotic indices. The taxa assigned using HTS (“Molecular”, left chart) and assigned in the morphotaxonomic inventory (“Morphology”, right chart) are associated with the total number of species (below numbers).

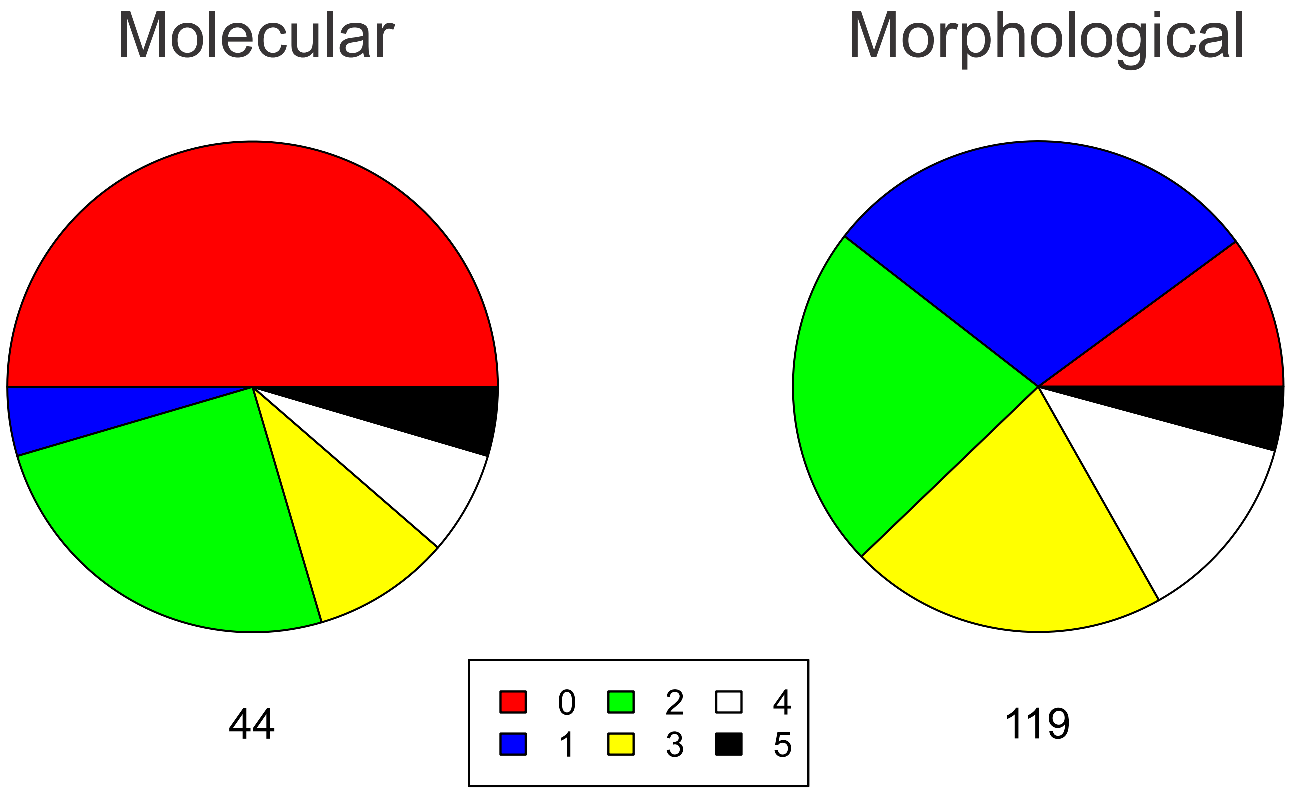

Molecular
Morphological
44
119
0
1
2
3
4
5

Supplementary Table 1. Sampling sites and metadata

Station	Date	LatDeg	LatMin	LonDeg	LonMin	Depth	Redox	Distance (m)
1	5/25/13	56	30.095	5	30.008	29.1	83	0
2	5/25/13	56	30.091	5	30.016	27.9	153	11
3	5/25/13	56	30.088	5	30.03	26.7	183	26
4	5/25/13	56	30.082	5	30.036	26.9	44	40
5	5/25/13	56	30.079	5	30.037	26.5	-2	50
6	5/25/13	56	30.077	5	30.055	26.1	-5	60
7	5/25/13	56	29.982	5	30.171	27	92	270
8	5/25/13	56	29.95	5	30.206	28.9	69	340
9	5/25/13	56	29.992	5	30.236	29.2	107	400
10	5/25/13	56	30.06	5	29.969	33.4	87	76

Supplementary Table 2. Tag-to-sample information and tagged primer sequences.

Molecule	Sample	Replicate	Forward	Reverse	Molecule	Sample	Replicate	Forward	Reverse
RNA	1	1	V4F-K	V4R-H	DNA	1	1	V4F-X	V4R-O
		2	V4F-K	V4R-T			2	V4F-X	V4R-U
		3	V4F-K	V4R-X			3	V4F-Y	V4R-H
		4	V4F-L	V4R-A			4	V4F-Y	V4R-L
		5	V4F-L	V4R-E			5	V4F-Y	V4R-N
	2	1	V4F-I	V4R-V		2	1	V4F-W	V4R-J
		2	V4F-J	V4R-A			2	V4F-W	V4R-N
		3	V4F-J	V4R-H			3	V4F-W	V4R-U
		4	V4F-J	V4R-W			4	V4F-X	V4R-G
		5	V4F-K	V4R-B			5	V4F-X	V4R-K
	3	1	V4F-A	V4R-J		3	1	V4F-N	V4R-A
		2	V4F-A	V4R-L			2	V4F-N	V4R-F
		3	V4F-A	V4R-N			3	V4F-N	V4R-W
		4	V4F-A	V4R-S			4	V4F-N	V4R-Y
		5	V4F-B	V4R-G			5	V4F-O	V4R-A
	4	1	V4F-B	V4R-K		4	1	V4F-O	V4R-B
		2	V4F-B	V4R-N			2	V4F-O	V4R-T
		3	V4F-B	V4R-O			3	V4F-O	V4R-X
		4	V4F-C	V4R-B			4	V4F-P	V4R-C
		5	V4F-C	V4R-G			5	V4F-P	V4R-I
	5	1	V4F-C	V4R-P		5	1	V4F-P	V4R-T
		2	V4F-C	V4R-V			2	V4F-Q	V4R-D
		3	V4F-D	V4R-I			3	V4F-Q	V4R-V
		4	V4F-D	V4R-M			4	V4F-Q	V4R-Z
		5	V4F-D	V4R-Q			5	V4F-R	V4R-B
	6	1	V4F-D	V4R-W		6	1	V4F-R	V4R-E
		2	V4F-E	V4R-I			2	V4F-R	V4R-V
		3	V4F-E	V4R-L			3	V4F-R	V4R-Y
		4	V4F-E	V4R-O			4	V4F-S	V4R-F

	5	V4F-E	V4R-R		5	V4F-S	V4R-M
7	1	V4F-F	V4R-C	7	1	V4F-S	V4R-P
	2	V4F-F	V4R-D		2	V4F-S	V4R-Q
	3	V4F-F	V4R-S		3	V4F-T	V4R-G
	4	V4F-F	V4R-Z		4	V4F-T	V4R-K
	5	V4F-G	V4R-F		5	V4F-T	V4R-S
8	1	V4F-G	V4R-M	8	1	V4F-T	V4R-Z
	2	V4F-G	V4R-T		2	V4F-U	V4R-H
	3	V4F-G	V4R-X		3	V4F-U	V4R-L
	4	V4F-H	V4R-J		4	V4F-U	V4R-W
	5	V4F-H	V4R-K		5	V4F-U	V4R-X
9	1	V4F-H	V4R-U	9	1	V4F-V	V4R-C
	2	V4F-H	V4R-Y		2	V4F-V	V4R-I
	3	V4F-I	V4R-D		3	V4F-V	V4R-Q
	4	V4F-I	V4R-E		4	V4F-V	V4R-R
	5	V4F-I	V4R-P		5	V4F-W	V4R-D
10	1	V4F-L	V4R-U	10	1	V4F-Y	V4R-R
	2	V4F-L	V4R-Y		2	V4F-Z	V4R-E
	3	V4F-M	V4R-C		3	V4F-Z	V4R-M
	4	V4F-M	V4R-F		4	V4F-Z	V4R-P
	5	V4F-M	V4R-Z		5	V4F-Z	V4R-S

Forward primer sequences (5'-3')	
V4F-A	ACACACACCCAGCASCYGCGGTAATTCC
V4F-B	ACGACTCTCCAGCASCYGCGGTAATTCC
V4F-C	ACGCTAGTCCAGCASCYGCGGTAATTCC
V4F-D	ACTATCATCCAGCASCYGCGGTAATTCC
V4F-E	ACTGCTGACCAGCASCYGCGGTAATTCC
V4F-F	AGACATCTCCAGCASCYGCGGTAATTCC
V4F-G	AGTCTACACCAGCASCYGCGGTAATTCC
V4F-H	CAGATCACCCAGCASCYGCGGTAATTCC
V4F-I	CATACTGCCCAGCASCYGCGGTAATTCC
V4F-J	CATATACTCCAGCASCYGCGGTAATTCC
V4F-K	CATCATATCCAGCASCYGCGGTAATTCC
V4F-L	CGACTCATCCAGCASCYGCGGTAATTCC
V4F-M	CGAGACGCCCAGCASCYGCGGTAATTCC
V4F-N	CGAGCACACCAGCASCYGCGGTAATTCC
V4F-O	CGTATCGACCAGCASCYGCGGTAATTCC
V4F-P	TAGACAGTCCAGCASCYGCGGTAATTCC
V4F-Q	TAGCACGACCAGCASCYGCGGTAATTCC
V4F-R	TATGACACCCAGCASCYGCGGTAATTCC
V4F-S	TATGTAGACCAGCASCYGCGGTAATTCC
V4F-T	TCAGCTACCCAGCASCYGCGGTAATTCC
V4F-U	TCAGTCGTCCAGCASCYGCGGTAATTCC
V4F-V	TCGATACACCAGCASCYGCGGTAATTCC
V4F-W	TCTCATCACCAGCASCYGCGGTAATTCC
V4F-X	TCTGCACTCCAGCASCYGCGGTAATTCC
V4F-Y	TGACTAGCCCAGCASCYGCGGTAATTCC
V4F-Z	TGTGATGTCCAGCASCYGCGGTAATTCC

Reverse primer sequences (5'-3')	
V4R-A	CAGAGACGACTTTCGTTCTTGATYRA
V4R-B	CAGATGACACTTTCGTTCTTGATYRA
V4R-C	CAGTATGCACTTTCGTTCTTGATYRA
V4R-D	CATAGTATACTTTCGTTCTTGATYRA
V4R-E	CATGTGCTACTTTCGTTCTTGATYRA
V4R-F	CGAGAGACACTTTCGTTCTTGATYRA
V4R-G	CGAGTACGACTTTCGTTCTTGATYRA
V4R-H	CGATGTAGACTTTCGTTCTTGATYRA
V4R-I	CGTATAGCACTTTCGTTCTTGATYRA
V4R-J	CGTGATGTACTTTCGTTCTTGATYRA
V4R-K	GAGATAGTACTTTCGTTCTTGATYRA
V4R-L	GAGTGTCTACTTTCGTTCTTGATYRA
V4R-M	GCATATACACTTTCGTTCTTGATYRA
V4R-N	GCATGACGACTTTCGTTCTTGATYRA
V4R-O	GCGAGTAGACTTTCGTTCTTGATYRA
V4R-P	GCTATGATACTTTCGTTCTTGATYRA
V4R-Q	GCTGATCGACTTTCGTTCTTGATYRA
V4R-R	TAGTAGAGACTTTCGTTCTTGATYRA
V4R-S	TATGATACACTTTCGTTCTTGATYRA
V4R-T	TCAGTAGTACTTTCGTTCTTGATYRA
V4R-U	TCATAGCTACTTTCGTTCTTGATYRA
V4R-V	TCGATGCGACTTTCGTTCTTGATYRA
V4R-W	TCTAGTGCACTTTCGTTCTTGATYRA
V4R-X	TGATGAGCACTTTCGTTCTTGATYRA
V4R-Y	TGTAGACTACTTTCGTTCTTGATYRA
V4R-Z	TGTGTGAGACTTTCGTTCTTGATYRA

Supplementary Table 3. Taxa-to-samples information. For each morphological sample (columns) are indicarted the number of specimens of each species sorted and identified and its ITI value.

Taxon	st.1	st.2	st.3	st.4	st.5	st.6	st.7	st.8	st.9	st.10
Metazoa	0	0	0	0	0	0	1	1	0	0
Metazoa|Annelida|Capitellida|Capitellidae|Capitella|Capitella+sp	5904	3395	1573	1409	1489	214	1	2	0	3
Metazoa|Annelida|Capitellida|Capitellidae|Mediomastus|Mediomastus+fragilis	0	0	9	2	0	0	1	0	0	0
Metazoa|Annelida|Capitellida|Capitellidae|Notomastus|Notomastus+sp	0	1	3	0	0	0	4	8	12	9
Metazoa|Annelida|Capitellida|Maldanidae|Praxillella|Praxillella+sp	0	0	0	0	0	0	0	1	0	0
Metazoa|Annelida|Capitellida|Maldanidae|Rhodine|Rhodine+loveni	0	0	0	0	0	0	1	1	0	2
Metazoa|Annelida|Capitellida|Scalibregmatidae	0	0	0	0	0	0	1	0	0	0
Metazoa|Annelida|Capitellida|Scalibregmatidae|Polyphysia|Polyphysia+crassa	0	1	0	0	0	0	0	1	1	0
Metazoa|Annelida|Capitellida|Scalibregmatidae|Scalibregma|Scalibregma+inflatum	0	0	3	1	2	2	0	1	0	0
Metazoa|Annelida|Eunicida|Dorvilleidae|Ophryotrocha	0	0	1	1	0	0	0	0	0	0
Metazoa|Annelida|Eunicida|Lumbrineridae|Abyssoninoe|Abyssoninoe+hibernica	0	0	0	0	0	0	0	1	1	0
Metazoa|Annelida|Eunicida|Lumbrineridae|Lumbrineris|Lumbrineris+aniara	0	0	0	0	0	0	4	3	2	0
Metazoa|Annelida|Eunicida|Onuphidae|Aponuphis|Aponuphis+bilineata	0	0	0	0	0	0	1	0	1	0
Metazoa|Annelida|Flabelligerida|Flabelligeridae|Diplocirrus|Diplocirrus+glaucus	0	0	0	0	0	0	3	2	2	1
Metazoa|Annelida|Oligochaeta|Naididae|Tubificoides|Tubificoides+benedii	42	60	134	23	3	2	0	0	0	0
Metazoa|Annelida|Phyllodocida|Aphroditidae|Aphrodita|Aphrodita+aculeata	0	0	0	0	0	0	1	0	0	0
Metazoa|Annelida|Phyllodocida|Glyceridae|Glycera|Glycera+alba	1	7	7	11	3	3	1	1	0	2
Metazoa|Annelida|Phyllodocida|Hesionidae|Oxydromus|Oxydromus+flexuosus	0	1	0	1	0	0	0	2	1	2
Metazoa|Annelida|Phyllodocida|Nephtyidae|Nephtys|Nephtys+incisa	0	0	0	0	0	0	1	3	1	6
Metazoa|Annelida|Phyllodocida|Nephtyidae|Nephtys|Nephtys+kersivalensis	0	0	1	0	0	0	0	0	0	0
Metazoa|Annelida|Phyllodocida|Nereididae	0	0	0	0	0	0	1	0	0	0
Metazoa|Annelida|Phyllodocida|Pholoidae|Pholoe|Pholoe+baltica	0	0	2	3	3	0	31	20	21	10
Metazoa|Annelida|Phyllodocida|Pholoidae|Pholoe|Pholoe+inornata	0	0	0	0	0	0	0	0	2	0
Metazoa|Annelida|Phyllodocida|Phyllodocidae|Eumida|Eumida+bahusiensis	0	1	0	0	0	0	0	4	3	5
Metazoa|Annelida|Phyllodocida|Phyllodocidae|Eumida|Eumida+sanguinea	0	0	3	2	0	0	0	0	2	0

Metazoa|Annelida|Phyllodocida|Phyllodocidae|Paranaitis|Paranaitis+kosteriensis	0	0	0	0	0	0	1	4	0	1
Metazoa|Annelida|Phyllodocida|Phyllodocidae|Phyllodoce|Phyllodoce+mucosa	0	0	1	1	0	0	0	0	0	0
Metazoa|Annelida|Phyllodocida|Phyllodocidae|Sige|Sige+fusigera	0	0	0	0	0	0	3	2	0	0
Metazoa|Annelida|Phyllodocida|Pilargidae|Ancistrosyllis|Ancistrosyllis+groenlandica	0	0	0	0	0	0	0	1	2	1
Metazoa|Annelida|Phyllodocida|Pilargidae|Litocorsa|Litocorsa+stremma	0	0	0	0	0	0	1	1	0	1
Metazoa|Annelida|Phyllodocida|Polynoidae|Malmgrenia|Malmgrenia+andreapolis	0	0	0	0	0	0	1	2	2	0
Metazoa|Annelida|Phyllodocida|Polynoidae|Malmgrenia|Malmgrenia+arenicolae	0	0	0	0	0	0	0	0	0	2
Metazoa|Annelida|Phyllodocida|Sphaerodoridae|Sphaerodorum|Sphaerodorum+gracilis	0	0	0	0	0	0	2	0	0	0
Metazoa|Annelida|Scolecida|Opheliidae|Ophelina|Ophelina+acuminata	0	0	0	0	0	0	0	0	1	0
Metazoa|Annelida|Scolecida|Paraonidae|Levinsenia|Levinsenia+gracilis	0	0	0	0	0	0	4	1	1	0
Metazoa|Annelida|Sipuncula|Golfingiidae|Thysanocardia|Thysanocardia+procera	0	0	0	0	0	0	4	2	3	0
Metazoa|Annelida|Spionida|Cirratulidae	0	0	0	0	0	0	1	0	0	0
Metazoa|Annelida|Spionida|Cirratulidae|Aphelochaeta|Aphelochaeta+sp	0	0	0	0	0	0	0	0	0	1
Metazoa|Annelida|Spionida|Cirratulidae|Caulleriella|Caulleriella+alata	0	0	7	6	0	0	0	0	0	0
Metazoa|Annelida|Spionida|Cirratulidae|Chaetozone|Chaetozone+zetlandica	0	1	0	0	0	1	0	0	1	0
Metazoa|Annelida|Spionida|Cirratulidae|Cirratulus|Cirratulus+sp	0	0	0	0	0	0	2	1	0	0
Metazoa|Annelida|Spionida|Cirratulidae|Cirriformia|Cirriformia+tentaculata	1	17	24	4	1	1	0	0	0	0
Metazoa|Annelida|Spionida|Cirratulidae|Protocirrineris|Protocirrineris+chrysoderma	0	0	0	0	0	0	0	0	1	0
Metazoa|Annelida|Spionida|Magelonidae|Magelona|Magelona+minuta	0	0	0	0	0	0	2	0	0	0
Metazoa|Annelida|Spionida|Spionidae|Malacoceros|Malacoceros+fuliginosus	227	343	62	36	34	1	0	0	0	1
Metazoa|Annelida|Spionida|Spionidae|Paraspio|Paraspio+decorata	0	0	0	1	0	0	0	0	0	0
Metazoa|Annelida|Spionida|Spionidae|Prionospio|Prionospio+fallax	0	0	0	0	0	0	1	2	0	1
Metazoa|Annelida|Spionida|Spionidae|Spiophanes|Spiophanes+kroeyeri	0	0	0	0	0	0	4	3	6	3
Metazoa|Annelida|Terebellida|Ampharetidae|Ampharete|Ampharete+lindstroemi	0	0	0	0	0	0	3	2	2	0
Metazoa|Annelida|Terebellida|Ampharetidae|Amphicteis|Amphicteis+gunneri	0	0	0	0	0	0	0	0	1	0
Metazoa|Annelida|Terebellida|Ampharetidae|Melinna|Melinna+palmata	0	0	0	0	0	0	28	9	1	0
Metazoa|Annelida|Terebellida|Amphitrite|Amphitrite|Amphitrite+cirrata	0	0	0	0	0	0	0	1	0	0
Metazoa|Annelida|Terebellida|Pectinariidae|Pectinaria|Pectinaria+belgica	0	0	0	0	0	0	0	0	1	0
Metazoa|Annelida|Terebellida|Pectinariidae|Pectinaria|Pectinaria+koreni	0	1	0	1	0	0	0	0	0	0

Metazoa|Annelida|Terebellida|Terebellidae|Amaeana|Amaeana+trilobata	0	0	0	0	0	0	1	2	4	2
Metazoa|Annelida|Terebellida|Terebellidae|Amphitritinae|Amphitritinae+sp	0	0	0	0	0	0	0	0	1	0
Metazoa|Annelida|Terebellida|Terebellidae|Axionice|Axionice+maculata	0	0	0	0	0	0	1	0	0	0
Metazoa|Annelida|Terebellida|Terebellidae|Polycirrus|Polycirrus+plumosus	0	0	0	0	0	0	17	17	24	13
Metazoa|Annelida|Terebellida|Terebellidae|Polycirrus|Polycirrus+sp	0	0	0	0	0	0	3	1	0	0
Metazoa|Annelida|Terebellida|Terebellidae|Terebellides|Terebellides+stroemii	0	0	0	0	0	0	20	2	1	0
Metazoa|Arthropoda|Chelicerata|Pycnogonida|Anoplodactylus|Anoplodactylus+petiolatus	0	0	0	0	0	0	0	0	0	1
Metazoa|Arthropoda|Crustacea|Malacostraca|Ampelisca|Ampelisca+diadema	0	0	0	0	0	0	1	1	0	0
Metazoa|Arthropoda|Crustacea|Malacostraca|Ampelisca|Ampelisca+tenuicornis	0	0	0	0	0	0	0	0	1	0
Metazoa|Arthropoda|Crustacea|Malacostraca|Autonoe|Autonoe+longipes	0	0	0	0	0	0	1	0	0	0
Metazoa|Arthropoda|Crustacea|Malacostraca|Decapoda	0	0	0	0	0	0	0	0	0	1
Metazoa|Arthropoda|Crustacea|Malacostraca|Decapoda|Decapoda_X	0	0	0	0	0	0	0	0	0	1
Metazoa|Arthropoda|Crustacea|Malacostraca|Eudorella|Eudorella+emarginata	0	0	0	0	0	0	1	0	0	0
Metazoa|Arthropoda|Crustacea|Malacostraca|Jassa|Jassa+sp	0	0	0	0	0	0	0	0	0	1
Metazoa|Arthropoda|Crustacea|Malacostraca|Leucothoe|Leucothoe+lilljeborgi	0	0	0	0	0	0	0	0	1	0
Metazoa|Arthropoda|Crustacea|Malacostraca|Microdeutopus|Microdeutopus+anomalus	0	0	2	1	0	2	0	0	0	0
Metazoa|Arthropoda|Crustacea|Malacostraca|Nebalia|Nebalia+herbstii	0	1	0	0	0	0	0	0	0	0
Metazoa|Arthropoda|Crustacea|Malacostraca|Pariambus|Pariambus+typicus	0	1	0	0	0	1	0	0	0	0
Metazoa|Arthropoda|Crustacea|Maxillopoda|Balanus|Balanus+sp	0	0	0	0	0	1	0	0	0	0
Metazoa|Arthropoda|Crustacea|Maxillopoda|Calanus|Calanus+finmarchicus	0	0	0	0	0	0	0	0	0	1
Metazoa|Arthropoda|Crustacea|Maxillopoda|Calanus|Calanus+helgolandicus	0	1	0	0	0	0	0	0	0	0
Metazoa|Arthropoda|Crustacea|Maxillopoda|Copepoda	0	0	0	0	0	1	0	0	0	0
Metazoa|Arthropoda|Crustacea|Maxillopoda|Verruca|Verruca+stroemia	0	0	0	0	0	0	0	0	2	0
Metazoa|Arthropoda|Hexapoda|Insecta|Chironomidae	0	0	1	0	0	0	0	0	0	0
Metazoa|Bryozoa	1	0	0	0	0	0	0	0	0	0
Metazoa|Cnidaria|Cnidaria_X|Anthozoa|Edwardsia|Edwardsia+claparedii	0	0	0	0	0	0	1	0	1	0
Metazoa|Cnidaria|Cnidaria_X|Anthozoa|Pennatula|Pennatula+phosphorea	0	0	0	0	0	0	0	0	1	0
Metazoa|Echinodermata|Echinodermata_X|Holothuroidea|Labidoplax|Labidoplax+sp	0	0	0	0	0	0	1	0	0	0
Metazoa|Echinodermata|Ophiuroidea	0	0	2	0	0	0	0	0	0	0

Metazoa|Echinodermata|Ophiuroidea|Amphiuridae	0	0	0	0	0	0	11	7	0	1
Metazoa|Echinodermata|Ophiuroidea|Amphiuridae|Amphiura|Amphiura+chiajei	0	0	0	0	0	0	12	10	7	11
Metazoa|Echinodermata|Ophiuroidea|Amphiuridae|Amphiura|Amphiura+filiformis	0	0	0	0	0	0	1	7	25	2
Metazoa|Entoprocta	0	1	0	0	0	0	0	0	5	0
Metazoa|Mollusca|Aplacophora|Aplacophora_X|Chaetoderma|Chaetoderma+nitidulum	0	0	0	0	0	0	0	0	1	0
Metazoa|Mollusca|Aplacophora|Aplacophora_X|Falcidens|Falcidens+crossotus	0	0	0	0	0	0	1	1	5	0
Metazoa|Mollusca|Aplacophora|Aplacophora_X|Scutopus|Scutopus+ventrolineatus	0	0	0	0	0	0	0	0	0	2
Metazoa|Mollusca|Bivalvia|Anomalodesmata|Thracia|Thracia+sp	0	0	2	4	5	6	0	0	0	0
Metazoa|Mollusca|Bivalvia|Heteroconchia|Abra|Abra+alba	0	0	12	1	0	0	1	0	1	0
Metazoa|Mollusca|Bivalvia|Heteroconchia|Abra|Abra+nitida	0	2	2	2	0	0	1	0	16	0
Metazoa|Mollusca|Bivalvia|Heteroconchia|Angulus|Angulus+fabula	0	0	1	0	0	0	0	0	0	0
Metazoa|Mollusca|Bivalvia|Heteroconchia|Angulus|Angulus+pygmaeus	0	0	1	0	0	0	0	0	0	0
Metazoa|Mollusca|Bivalvia|Heteroconchia|Chamelea|Chamelea+striatula	0	0	0	0	0	0	0	1	0	0
Metazoa|Mollusca|Bivalvia|Heteroconchia|Myrtea|Myrtea+spinifera	0	0	0	0	0	0	5	7	2	0
Metazoa|Mollusca|Bivalvia|Heteroconchia|Parvicardium|Parvicardium+scabrum	0	0	0	0	0	0	0	1	0	0
Metazoa|Mollusca|Bivalvia|Heteroconchia|Venerupis|Venerupis+corrugata	0	0	0	1	0	0	0	0	0	0
Metazoa|Mollusca|Bivalvia|Heterodonta|Corbula|Corbula+gibba	0	0	0	0	0	0	24	19	1	8
Metazoa|Mollusca|Bivalvia|Heterodonta|Kurtiella|Kurtiella+bidentata	1	0	0	0	0	0	14	22	17	15
Metazoa|Mollusca|Bivalvia|Lucinoida|Thyasira|Thyasira+flexuosa	0	4	0	1	0	3	3	2	2	3
Metazoa|Mollusca|Bivalvia|Mytiloida|Modiolus|Modiolus+modiolus	0	0	1	0	0	0	0	0	0	0
Metazoa|Mollusca|Bivalvia|Mytiloida|Mytilus|Mytilus+edulis	0	0	1	0	1	0	0	0	0	0
Metazoa|Mollusca|Bivalvia|Protobranchia|Nucula|Nucula+nitidosa	0	0	0	0	0	0	3	0	3	0
Metazoa|Mollusca|Bivalvia|Protobranchia|Nucula|Nucula+sulcata	0	0	0	0	0	1	0	0	2	1
Metazoa|Mollusca|Gastropoda|Caenogastropoda|Turritella|Turritella+communis	0	0	0	0	0	0	5	3	3	0
Metazoa|Mollusca|Gastropoda|Heterobranchia|Alvania|Alvania+testae	0	0	0	0	0	0	0	0	1	0
Metazoa|Mollusca|Gastropoda|Heterobranchia|Bela|Bela+brachystoma	0	0	0	0	0	0	0	2	0	0
Metazoa|Mollusca|Gastropoda|Heterobranchia|Crisilla|Crisilla+semistriata	0	0	0	0	0	3	0	0	0	0
Metazoa|Mollusca|Gastropoda|Heterobranchia|Cylichna|Cylichna+cylindracea	0	0	0	0	0	0	2	3	0	2
Metazoa|Mollusca|Gastropoda|Heterobranchia|Hyala|Hyala+vitrea	24	41	0	0	0	0	51	25	16	10

Metazoa|Mollusca|Gastropoda|Heterobranchia|Odostomia|Odostomia+sp	0	0	0	2	0	0	1	0	0	0
Metazoa|Mollusca|Gastropoda|Heterobranchia|Opisthobranchia	0	1	0	0	0	0	0	0	0	0
Metazoa|Nematoda	0	0	0	0	0	0	0	1	0	0
Metazoa|Nemertea	883	506	415	166	154	124	2	0	0	5
Metazoa|Priapulida|Priapulida_X|Priapulida_XX|Priapulus|Priapulus+caudatus	0	0	0	0	0	0	11	3	4	0

Supplementary Table 4. Sequencing statistics for raw data filtering and metazoa sequence selection

		DNA			RNA		
Sample	Replicate	Total	Filtered	Metazoa	Total	Filtered	Metazoa
1	1	63	0	0	64701	41540	5722
	2	277	0	0	74863	50935	4984
	3	360	2	2	101137	72790	7684
	4	186	0	0	75131	49745	21914
	5	32927	23950	14364	81610	53277	8673
2	1	63467	45859	19807	56900	33003	8047
	2	46872	34143	16822	89899	57407	13884
	3	47392	36746	26435	78981	51026	18216
	4	41341	25101	15108	89462	60725	33207
	5	101	0	0	69936	42371	6460
3	1	54005	40193	30808	51313	32803	2705
	2	54667	37664	21788	27349	16034	719
	3	95313	70045	58621	47045	27471	11867
	4	57515	35555	24463	47743	29308	4281
	5	75916	56167	52189	48787	27251	6154
4	1	57239	40770	33092	33301	18305	8908
	2	40740	26360	11194	31227	16649	3923
	3	45131	30293	14546	14876	7816	3761
	4	53333	34802	17818	48072	27111	5496
	5	44681	27890	13242	54618	29191	13436
5	1	35220	24260	7450	34641	19318	2630
	2	38470	25747	7560	45227	24889	3471
	3	34080	25702	24721	56538	38415	25037
	4	45308	30633	14736	24479	13913	4752

	5	40271	26089	7658	18723	9634	2736
6	1	42069	31596	12402	46909	32947	4962
	2	35947	26029	18188	45782	31084	1708
	3	51948	38820	10639	22990	13858	1711
	4	59477	43298	15456	18149	9467	1338
	5	26835	18071	6474	37655	23826	2885
7	1	24552	13211	2353	75799	45633	6542
	2	17914	9176	9030	29187	16430	3064
	3	24058	12130	4844	33002	19273	568
	4	20908	12893	2292	34272	22315	5420
	5	30718	15147	3220	7261	3812	331
8	1	44719	29208	1911	16850	10080	134
	2	13278	7455	2067	28189	18872	437
	3	19570	12833	1317	36023	24073	1831
	4	61004	43801	27826	37888	24264	3144
	5	66132	42238	8915	21188	13057	690
9	1	55438	38000	840	92087	64950	954
	2	45706	27590	1829	81749	58978	859
	3	17046	8120	827	51098	28706	185
	4	29596	19431	1899	74333	46053	1408
	5	49640	37093	1028	43557	27191	117
10	1	33266	20322	10985	75296	53879	14649
	2	53124	40025	1887	96476	67556	387
	3	30109	20822	198	77214	50313	83
	4	25442	15863	1786	57201	36535	6569
	5	40795	26600	2937	60905	40262	7084

Supplementary table 5. Pairwise Wilcoxon Mann-Whitney tests (False Discovery Rate correction)

Pairwise comparisons of the number of specimens among morphology samples

	3	4	5	6	7	8	9	2	1
3	0.709	-	-	-	-	-	-	-	-
4	0.015**	0.035*	-	-	-	-	-	-	-
5	0.139	0.268	0.308	-	-	-	-	-	-
6	0.001***	0***	0***	0***	-	-	-	-	-
7	0.005***	0.001***	0***	0***	0.594	-	-	-	-
8	0.004***	0.001***	0***	0***	0.594	0.960	-	-	-
9	0.437	0.659	0.090	0.537	0***	0***	0***	-	-
2	0.008***	0.019**	0.838	0.210	0***	0***	0***	0.057	-
1	0.253	0.112	0***	0.006***	0.021**	0.088	0.088	0.04*	0***

Pairwise comparisons of the number of sequence reads among DNA samples

	3	4	5	6	7	8	9	2	1
3	0.119	-	-	-	-	-	-	-	-
4	0.079	0.455	-	-	-	-	-	-	-
5	0.036*	0.498	0.901	-	-	-	-	-	-
6	0.036*	0.036*	0.119	0.051	-	-	-	-	-
7	0.079	0.186	0.498	0.37	0.616	-	-	-	-
8	0.036*	0.036*	0.036*	0.036*	0.036*	0.079	-	-	-
9	0.13	0.498	0.2	0.2	0.051	0.33	0.051	-	-
2	0.455	1	1	1	0.455	0.732	0.455	0.498	-
1	0.036	0.036*	0.119	0.079	0.455	0.616	0.498	0.051	0.455

Pairwise comparisons of the number of sequence reads among RNA samples

	3	4	5	6	7	8	9	2	1
3	0.77	-	-	-	-	-	-	-	-
4	1	0.6	-	-	-	-	-	-	-
5	0.676	0.13	0.526	-	-	-	-	-	-
6	0.77	0.399	0.818	0.923	-	-	-	-	-
7	0.19	0.089	0.13	0.6	0.676	-	-	-	-
8	0.19	0.089	0.089	0.089	0.526	0.818	-	-	-
9	0.13	0.19	0.312	0.089	0.089	0.089	0.089	-	-
2	0.818	1	0.818	0.6	0.6	0.6	0.6	0.923	-
1	1	0.923	1	0.818	0.77	0.77	0.676	0.19	0.818

Supplementary table 6. Pairwise Wilcoxon Mann-Whitney tests among DNA or among RNA samples (False Discovery Rate correction)

Pairwise comparisons of the number of OTUs among DNA samples

	3	4	5	6	7	8	9	2	1
3	0.342	-	-	-	-	-	-	-	-
4	0.655	0.655	-	-	-	-	-	-	-
5	0.977	0.280	0.531	-	-	-	-	-	-
6	0.001	0.002	0.001	0.000	-	-	-	-	-
7	0.021	0.031	0.016	0.004	0.330	-	-	-	-
8	0.074	0.251	0.159	0.031	0.003	0.087	-	-	-
9	0.251	0.019	0.087	0.211	0.000	0.003	0.003	-	-
2	0.004	0.002	0.003	0.003	0.002	0.003	0.002	0.008	-
1	0.000	0.000	0.000	0.000	0.000	0.000	0.000	0.003	0.061

Pairwise comparisons of the number of OTUs among RNA samples

	3	4	5	6	7	8	9	2	1
3	0.742	-	-	-	-	-	-	-	-
4	0.728	0.930	-	-	-	-	-	-	-
5	0.413	0.779	0.724	-	-	-	-	-	-
6	0.877	0.723	0.651	0.325	-	-	-	-	-
7	0.355	0.553	0.533	0.799	0.330	-	-	-	-
8	0.000	0.012	0.002	0.002	0.001	0.036	-	-	-
9	0.877	0.877	0.880	0.557	0.724	0.413	0.001	-	-
2	0.006	0.079	0.036	0.072	0.012	0.238	0.413	0.017	-
1	0.000	0.002	0.001	0.001	0.001	0.004	0.352	0.001	0.091

Supplementary Table 7. OTUs-to-samples information. For each molecular sample (columns) are indicated the normalized number of sequences of each OTU and its taxonomic assignment. The samples names encode the molecule type (DNA or RNA), the station name (from 1 to 10) and the number if the PCR replicate within the station (from 1 to 5).

OTU	assign	DNA_1_1	DNA_2_1	DNA_2_2	DNA_2_3	DNA_2_4	DNA_3_1	DNA_3_2	DNA_3_3	DNA_3_4	DNA_3_5
OTU1	Metazoa|Annelida|Capitellida|Capitellidae|Capitella	3722.54	1264.01	3208.26	3816.66	588.22	3751.88	3641.11	3838.88	2314.34	3183.09
OTU2	Metazoa|Annelida|Oligochaeta|Tubificidae	35.08	245.9	0	5.36	168.93	218.82	393.83	52.72	628.32	870.89
OTU4	Metazoa|Annelida|Phyllodocida|Phyllodocidae|Phyllodoce|Phyllodoce+maculata	29.88	1354.27	160.2	26.85	126.35	15.04	8.51	73.98	43.34	2.82
OTU5	Metazoa|Annelida|Spionida|Spionidae|Malacoceros|Malacoceros+fuliginosus	91.71	748.37	100.31	0	3018.35	0	0	0	0	0
OTU6	Metazoa|Annelida|Spionida|Cirratulidae	0	0	0	0	0	0	0	92.75	147.04	1.34
OTU7	Metazoa|Platyhelminthes	221.85	403.5	524.53	138.1	170.58	0	29.75	9.6	34.29	22.44
OTU8	Metazoa|Annelida|Phyllodocida|Syllidae|Salvatoria	0	0	0	0	0	0	5.85	0	894	0
OTU9	Metazoa|Annelida	0	0	2.69	5.06	14.16	0	0	0	0	0
OTU10	Metazoa|Nematoda	0	10.35	0	0	0	0	0	0	0	0
OTU11	Metazoa|Nematoda	0	1.86	0	3.82	0	0	1.05	1.88	0	0
OTU12	Metazoa|Nematoda	0	6.37	0	0	1.38	6.2	3.73	0	3.51	0
OTU13	Metazoa|Cnidaria|Cnidaria_X|Anthozoa	0	0	0	104.16	1.11	94.19	0	21.32	0	0
OTU14	Metazoa|Nemertea	0	0	0	0	0	0	0	0	0	0
OTU15	Metazoa|Mollusca|Bivalvia|Heteroconchia|Abra	0	0	0	0	0	0	0	0	0	1.02
OTU16	Metazoa|Platyhelminthes	0	0	0	0	0	0	0	0	0	0
OTU17	Metazoa|Cnidaria|Cnidaria_X|Hydrozoa	0	0	0	0	0	0	0	0	0	0
OTU18	Metazoa|Platyhelminthes	0	0	52.67	0	0	1.64	3.17	0	0	1.26
OTU19	Metazoa|Arthropoda|Crustacea|Ostracoda	0	0	0	0	0	0	0	0	0	0
OTU20	Metazoa|Nematoda	0	1.74	0	0	0	0	1.2	0	7.81	0
OTU21	Metazoa|Annelida|Terebellida|Ampharetidae	0	0	0	0	0	0	0	0	0	0
OTU22	Metazoa|Annelida|Phyllodocida|Syllidae|Exogone|Exogone+naidina	0	0	44.6	0	0	0	0	0	0	0
OTU23	Metazoa|Platyhelminthes	0	0	0	0	0	0	0	0	0	0
OTU24	Metazoa|Platyhelminthes	0	33.95	0	0	0	0	0	0	0	0

OTU25	Metazoa|Mollusca|Bivalvia|Heteroconchia|Corbula	0	0	0	0	0	0	0	0	0	0
OTU26	Metazoa|Annelida|Capitellida|Capitellidae|Heteromastus	0	0	0	0	0	2.82	2.54	0	4.25	0
OTU27	Metazoa|Arthropoda|Crustacea|Maxillopoda|Copepoda	0	0	0	0	0	0	0	0	0	0
OTU28	Metazoa|Nematoda	0	12.15	1.53	0	0	4.99	6.05	0	10.15	0
OTU29	Metazoa|Cnidaria|Cnidaria_X|Hydrozoa	0	0	0	0	0	0	0	0	0	0
OTU30	Metazoa|Mollusca|Gastropoda|Heterobranchia|Bulla	0	0	0	0	0	0	0	0	0	0
OTU31	Metazoa|Platyhelminthes	0	0	0	0	0	0	0	0	0	0
OTU32	Metazoa|Arthropoda|Crustacea|Ostracoda	0	0	0	0	10.28	0	0	0	0	0
OTU33	Metazoa|Nemertea	0	0	0	0	0	0	0	0	0	0
OTU35	Metazoa|Nemertea	0	0	0	0	0	0	0	0	0	0
OTU36	Metazoa|Arthropoda|Chelicerata	0	0	0	0	0	0	0	0	0	0
OTU37	Metazoa|Annelida|Phyllodocida|Syllidae|Erinaceusyllis|Erinaceusyllis+cryptica	0	0	3.26	0	0	0	0	0	0	0
OTU38	Metazoa|Platyhelminthes	0	0	0	0	0	0	0	0	0	0
OTU39	Metazoa|Arthropoda|Crustacea|Maxillopoda|Copepoda	0	6.47	1.05	1.51	0	0	0	0	0	0
OTU40	Metazoa|Annelida|Capitellida|Maldanidae|Praxillella	0	0	0	0	0	0	0	0	0	0
OTU41	Metazoa|Arthropoda|Crustacea|Maxillopoda|Copepoda	0	0	0	0	0	0	0	0	0	0
OTU42	Metazoa|Annelida|Phyllodocida|Syllidae|Exogone|Exogone+naidina	0	0	0	0	0	0	0	2.6	0	0
OTU43	Metazoa|Hemichordata	0	0	0	0	0	0	0	0	0	0
OTU44	Metazoa|Cnidaria|Cnidaria_X|Hydrozoa	0	7.14	0	0	0	0	0	0	0	0
OTU45	Metazoa|Mollusca|Bivalvia|Protobranchia|Nucula	0	0	0	0	0	0	0	0	0	0
OTU46	Metazoa|Annelida|Capitellida|Capitellidae|Capitella	0	0	0	0	0	0	0	0	0	0
OTU47	Metazoa|Platyhelminthes	0	0	0	0	0	0	0	0	0	0
OTU48	Metazoa|Platyhelminthes	0	0	0	0	0	0	0	0	0	0
OTU49	Metazoa|Platyhelminthes	0	0	0	0	0	0	0	0	0	0
OTU51	Metazoa|Nematoda	0	0	0	0	0	0	0	0	0	0
OTU52	Metazoa|Annelida|Capitellida|Maldanidae	0	0	0	0	0	0	0	0	0	0
OTU53	Metazoa|Nematoda	0	1.46	0	0	0	0	0	0	0	0
OTU54	Metazoa|Annelida|Oligochaeta|Tubificidae	0	0	0	0	0	2.54	0	0	0	11.28
OTU55	Metazoa|Platyhelminthes	0	0	0	0	0	0	0	0	0	0

OTU56	Metazoa|Platyhelminthes	0	0	0	0	0	0	0	0	0	0
OTU58	Metazoa|Nematoda	0	0	0	0	0	0	0	0	0	0
OTU59	Metazoa|Echinodermata|Echinodermata_X|Echinodermata_XX|Amphiura	0	0	0	0	0	0	0	0	0	0
OTU60	Metazoa|Platyhelminthes	0	0	0	0	0	0	0	0	0	0
OTU61	Metazoa|Cnidaria|Cnidaria_X|Hydrozoa	0	0	0	0	0	0	0	0	0	0
OTU62	Metazoa|Platyhelminthes	0	0	0	0	0	0	0	0	0	0
OTU64	Metazoa|Arthropoda|Crustacea|Maxillopoda|Copepoda	0	0	0	0	0	0	0	0	0	0
OTU65	Metazoa|Nematoda	0	0	0	0	0	0	0	0	0	0
OTU66	Metazoa|Arthropoda|Crustacea|Maxillopoda|Copepoda	0	0	0	0	0	0	0	0	0	0
OTU68	Metazoa|Platyhelminthes	0	0	0	0	0	0	0	0	0	0
OTU69	Metazoa|Platyhelminthes	0	0	0	0	0	0	0	0	0	0
OTU70	Metazoa|Arthropoda|Crustacea|Maxillopoda|Copepoda	0	0	0	0	0	0	0	0	0	0
OTU71	Metazoa|Platyhelminthes	0	0	0	0	0	0	0	0	0	0
OTU72	Metazoa|Nematoda	0	0	0	0	0	0	1.36	0	2.63	0
OTU73	Metazoa|Nematoda	0	0	0	0	0	0	0	0	0	0
OTU75	Metazoa|Gastrotricha	0	0	0	0	0	0	0	0	0	0
OTU76	Metazoa|Arthropoda|Crustacea|Maxillopoda|Copepoda	0	0	0	0	0	0	0	0	0	0
OTU78	Metazoa|Mollusca|Gastropoda|Heterobranchia|Calliopaea|Calliopaea+bellula	0	0	0	0	0	0	0	0	0	0
OTU79	Metazoa|Annelida|Phyllodocida|Syllidae	0	0	0	0	0	0	0	0	9.31	0
OTU80	Metazoa|Echinodermata|Echinodermata_X|Echinodermata_XX|Leptosynapta	0	0	0	0	0	0	0	0	0	0
OTU82	Metazoa|Annelida|Capitellida|Capitellidae|Capitella	0	0	0	0	0	1.06	0	0	1.08	4.05
OTU84	Metazoa|Annelida|Capitellida|Capitellidae|Capitella	0	0	0	0	0	0	0	0	0	0
OTU85	Metazoa|Platyhelminthes	0	0	0	0	0	0	0	0	0	0
OTU86	Metazoa|Platyhelminthes	0	0	0	0	0	0	0	4.44	0	0
OTU87	Metazoa|Annelida|Terebellida|Terebellidae	0	0	0	0	0	0	0	0	0	0
OTU88	Metazoa|Platyhelminthes	0	0	1.59	0	0	0	1.73	0	0	2.29
OTU89	Metazoa|Annelida|Oligochaeta|Tubificidae	0	0	0	0	0	0	0	0	0	0
OTU90	Metazoa|Cnidaria|Cnidaria_X|Anthozoa|Ceriantheopsis	0	0	0	0	0	0	0	0	0	0
OTU91	Metazoa|Annelida|Capitellida|Scalibregmatidae|Scalibregma|Scalibregma+inflatum	0	0	0	0	0	0	0	0	0	0

OTU92	Metazoa|Annelida|Scolecida|Opheliidae|Ophelina	0	0	0	0	0	0	0	0	0	0
OTU93	Metazoa|Gastrotricha	0	0	0	0	0	0	0	0	0	0
OTU94	Metazoa|Platyhelminthes	0	0	0	0	0	0	0	0	0	0
OTU95	Metazoa|Arthropoda|Crustacea|Ostracoda	0	0	0	0	0	0	0	0	0	0
OTU96	Metazoa|Platyhelminthes	0	0	0	0	0	0	0	0	0	0
OTU97	Metazoa|Platyhelminthes	0	0	0	0	0	0	0	0	0	0
OTU98	Metazoa|Platyhelminthes	0	0	0	0	0	0	0	0	0	0
OTU99	Metazoa|Platyhelminthes	0	0	0	0	0	0	0	0	0	0
OTU100	Metazoa|Arthropoda|Crustacea|Ostracoda	0	0	0	0	0	0	0	0	0	0
OTU101	Metazoa|Annelida|Phyllodocida|Phyllodocidae	0	0	0	0	0	0	0	0	0	0
OTU102	Metazoa|Arthropoda|Crustacea|Maxillopoda|Copepoda	0	0	0	0	0	0	0	0	0	0
OTU103	Metazoa|Nemertea	0	0	0	0	0	0	0	0	0	0
OTU104	Metazoa|Annelida|Phyllodocida|Syllidae	0	0	0	0	0	0	0	0	0	0
OTU107	Metazoa|Arthropoda|Crustacea|Ostracoda	0	0	0	0	0	0	0	0	0	0
OTU108	Metazoa|Annelida|Spionida|Spionidae|Malacoceros|Malacoceros+fuliginosus	0	3.04	0	0	0	0	0	0	0	0
OTU109	Metazoa|Arthropoda|Crustacea|Maxillopoda|Copepoda	0	0	0	0	0	0	0	0	0	0
OTU110	Metazoa|Arthropoda|Crustacea|Maxillopoda|Copepoda	0	0	0	0	0	0	0	0	0	0
OTU111	Metazoa|Kinorhyncha	0	0	0	0	0	0	0	0	0	0
OTU112	Metazoa|Platyhelminthes	0	0	0	0	0	0	0	0	0	0
OTU113	Metazoa|Nematoda	0	0	0	0	0	0	0	0	0	0
OTU114	Metazoa|Nematoda	0	0	0	0	0	0	0	0	0	0
OTU115	Metazoa	0	0	0	0	0	0	0	0	0	0
OTU116	Metazoa|Platyhelminthes	0	0	0	0	0	0	0	0	0	0
OTU117	Metazoa|Nematoda	0	0	0	0	0	0	0	0	0	0
OTU118	Metazoa|Arthropoda|Crustacea|Maxillopoda|Copepoda	0	0	0	0	1.25	0	0	0	0	0
OTU119	Metazoa|Nematoda	0	0	0	0	0	0	0	0	0	0
OTU120	Metazoa|Platyhelminthes	0	0	0	0	0	0	0	0	0	0
OTU121	Metazoa|Nematoda	0	0	0	0	0	0	0	0	0	0
OTU122	Metazoa|Annelida|Oligochaeta|Tubificidae	0	0	0	0	0	0	0	0	0	0

OTU123	Metazoa|Mollusca|Bivalvia|Heteroconchia|Myrtea|Myrtea+spinifera	0	0	0	0	0	0	0	0	0	0
OTU124	Metazoa|Annelida|Terebellida|Pectinariidae|Pectinaria	0	0	0	0	0	0	0	0	0	0
OTU125	Metazoa|Platyhelminthes	0	0	0	0	0	0	0	0	0	0
OTU126	Metazoa|Nematoda	0	0	0	0	0	0	0	0	0	0
OTU128	Metazoa|Kinorhyncha	0	0	0	0	0	0	0	0	0	0
OTU130	Metazoa|Annelida|Phyllodocida|Phyllodocidae	0	0	0	0	0	0	0	0	0	0
OTU131	Metazoa|Annelida|Oligochaeta|Tubificidae	0	0	0	0	0	0	0	0	0	0
OTU132	Metazoa|Nematoda	0	0	0	0	0	0	0	0	0	0
OTU134	Metazoa|Annelida|Phyllodocida|Syllidae	0	0	0	0	0	0	0	0	0	0
OTU135	Metazoa|Annelida|Capitellida|Capitellidae|Capitella	0	0	0	0	0	0	0	0	0	0
OTU136	Metazoa|Nematoda	0	0	0	0	0	0	0	0	0	0
OTU137	Metazoa|Hemichordata	0	0	0	0	0	0	0	0	0	0
OTU138	Metazoa	0	0	0	0	0	0	0	0	0	0
OTU141	Metazoa|Nematoda	0	0	0	0	0	0	0	0	0	0
OTU143	Metazoa|Annelida|Capitellida|Capitellidae|Capitella	0	0	0	0	0	0	0	0	0	0
OTU144	Metazoa|Platyhelminthes	0	0	0	0	0	0	0	0	0	0
OTU145	Metazoa|Annelida|Spionida|Spionidae|Malacoceros|Malacoceros+fuliginosus	0	0	0	0	0	0	0	0	0	0
OTU146	Metazoa|Arthropoda|Crustacea|Maxillopoda|Copepoda	0	0	0	0	0	0	0	0	0	0
OTU147	Metazoa|Porifera	0	0	0	0	0	0	0	0	0	0
OTU149	Metazoa|Mollusca|Bivalvia|Heteroconchia|Macoma|Macoma+nasuta	0	0	0	0	0	0	0	0	0	0
OTU151	Metazoa|Nematoda	0	0	0	0	0	0	0	0	0	0
OTU152	Metazoa|Platyhelminthes	0	0	0	0	0	0	0	0	0	0
OTU153	Metazoa|Annelida|Phyllodocida|Phyllodocidae	0	0	0	0	0	0	0	0	0	0
OTU154	Metazoa|Annelida|Spionida|Cirratulidae	0	0	0	0	0	0	0	0	0	0
OTU155	Metazoa|Annelida|Spionida|Cirratulidae	0	0	0	0	0	0	0	0	0	0
OTU156	Metazoa|Arthropoda|Crustacea|Maxillopoda|Copepoda	0	0	0	0	0	0	0	0	0	0
OTU157	Metazoa|Annelida|Phyllodocida|Phyllodocidae	0	0	0	0	0	0	0	0	0	0
OTU158	Metazoa|Annelida|Phyllodocida|Phyllodocidae	0	0	0	0	0	0	0	0	0	0
OTU159	Metazoa|Rotifera	0	0	0	0	0	0	0	0	0	0

OTU160	Metazoa|Arthropoda|Crustacea|Maxillopoda|Copepoda	0	0	0	0	0	0	0	0	0	0
OTU161	Metazoa|Platyhelminthes	0	0	0	0	0	0	0	0	0	0
OTU162	Metazoa|Annelida|Capitellida|Capitellidae|Capitella	0	0	0	0	0	0	0	0	0	0
OTU163	Metazoa|Nematoda	0	0	0	0	0	0	0	0	0	0
OTU164	Metazoa|Nematoda	0	0	0	0	0	0	0	0	0	0
OTU165	Metazoa|Nematoda	0	0	0	0	0	0	0	0	0	0
OTU167	Metazoa|Nematoda	0	0	0	0	0	0	0	0	0	0
OTU168	Metazoa|Cnidaria|Cnidaria_X|Hydrozoa	0	0	0	0	0	0	0	0	0	0
OTU169	Metazoa|Arthropoda|Crustacea|Maxillopoda|Copepoda	0	0	0	0	0	0	0	0	0	0
OTU170	Metazoa|Gastrotricha	0	0	0	0	0	0	0	0	0	0
OTU171	Metazoa|Annelida|Phyllodocida|Syllidae	0	0	0	0	0	0	0	0	0	0
OTU173	Metazoa|Nematoda	0	0	0	0	0	0	0	0	0	0
OTU174	Metazoa|Arthropoda|Crustacea|Maxillopoda|Copepoda	0	0	0	0	0	0	0	0	0	0
OTU175	Metazoa|Arthropoda|Crustacea|Maxillopoda|Copepoda	0	0	0	0	0	0	0	0	0	0
OTU176	Metazoa|Platyhelminthes	0	0	0	0	0	0	0	0	0	0
OTU178	Metazoa|Nematoda	0	0	0	0	0	0	0	0	0	0
OTU180	Metazoa|Nematoda	0	0	0	0	0	0	0	0	0	0
OTU183	Metazoa|Annelida|Oligochaeta|Tubificidae	0	0	0	0	0	0	0	0	0	0
OTU184	Metazoa|Nematoda	0	0	0	0	0	0	0	0	0	0
OTU186	Metazoa|Platyhelminthes	0	0	0	0	0	0	0	0	0	0
OTU188	Metazoa|Annelida|Terebellida|Ampharetidae	0	0	0	0	0	0	0	0	0	0
OTU190	Metazoa|Annelida|Terebellida|Terebellidae	0	0	0	0	0	0	0	0	0	0
OTU191	Metazoa|Annelida|Phyllodocida|Syllidae	0	0	0	0	0	0	0	0	0	0
OTU192	Metazoa|Annelida|Spionida|Cirratulidae	0	0	0	0	0	0	0	0	0	0

Supplementary Table 7. continued

OTU	assign	DNA_4_1	DNA_4_2	DNA_4_3	DNA_4_4	DNA_4_5	DNA_5_1	DNA_5_2	DNA_5_3	DNA_5_4	DNA_5_5
OTU1	Metazoa|Annelida|Capitellida|Capitellidae|Capitella	3359.67	345.91	1218.95	0	0	2170.69	1.23	3770.13	433.46	5.35
OTU2	Metazoa|Annelida|Oligochaeta|Tubificidae	259.49	1942.06	2285.42	2745.99	2317.17	2.13	379.21	50.59	2941.38	2151.51
OTU4	Metazoa|Annelida|Phyllodocida|Phyllodocidae|Phyllodoce|Phyllodoce+maculata	15.5	67.09	82.86	27.73	0	100.93	2.14	41.87	392.74	1587.72
OTU5	Metazoa|Annelida|Spionida|Spionidae|Malacoceros|Malacoceros+fuliginosus	0	0	0	0	0	0	0	0	0	0
OTU6	Metazoa|Annelida|Spionida|Cirratulidae	321.84	1456.94	403.71	1107.08	1208.68	1358.18	922.58	211.8	199.64	0
OTU7	Metazoa|Platyhelminthes	14.55	61.42	0	42.15	18.19	0	44.05	0	51.99	90.83
OTU8	Metazoa|Annelida|Phyllodocida|Syllidae|Salvatoria	0	0	0	0	0	0	2365.25	11.41	0	0
OTU9	Metazoa|Annelida	0	0	0	0	0	0	65.09	0	0	0
OTU10	Metazoa|Nematoda	0	0	0	0	91.13	0	0	0	0	0
OTU11	Metazoa|Nematoda	4.17	3.78	10.99	0	0	192.14	126.24	4.1	15.15	96.52
OTU12	Metazoa|Nematoda	23.47	122.38	37.99	133.24	119.75	38.7	15.92	1.71	5.42	25.91
OTU13	Metazoa|Cnidaria|Cnidaria_X|Anthozoa	0	0	0	0	0	0	0	0	0	0
OTU14	Metazoa|Nemertea	0	0	0	0	0	0	0	0	0	0
OTU15	Metazoa|Mollusca|Bivalvia|Heteroconchia|Abra	0	0	0	0	0	0	0	0	0	0
OTU16	Metazoa|Platyhelminthes	0	0	0	0	0	0	0	0	0	0
OTU17	Metazoa|Cnidaria|Cnidaria_X|Hydrozoa	2.64	0	0	0	0	0	0	0	0	0
OTU18	Metazoa|Platyhelminthes	0	19.88	0	0	23.84	1.68	56.99	0	15.83	0
OTU19	Metazoa|Arthropoda|Crustacea|Ostracoda	0	0	0	0	0	0	0	0	0	0
OTU20	Metazoa|Nematoda	0	15.18	20.48	11.89	10.1	44.44	53.9	5.96	8.47	47.12
OTU21	Metazoa|Annelida|Terebellida|Ampharetidae	0	0	0	0	0	0	0	0	0	0
OTU22	Metazoa|Annelida|Phyllodocida|Syllidae|Exogone|Exogone+naidina	0	0	0	0	0	0	0	0	0	0
OTU23	Metazoa|Platyhelminthes	0	0	0	0	0	0	0	0	0	0
OTU24	Metazoa|Platyhelminthes	0	0	0	0	0	0	0	0	0	0
OTU25	Metazoa|Mollusca|Bivalvia|Heteroconchia|Corbula	0	0	0	0	0	0	0	0	0	0
OTU26	Metazoa|Annelida|Capitellida|Capitellidae|Heteromastus	3.64	0	12.68	0	34.37	65.18	0	2.94	0	0

OTU27	Metazoa|Arthropoda|Crustacea|Maxillopoda|Copepoda	0	0	0	0	0	0	0	0	0	0
OTU28	Metazoa|Nematoda	1.48	14.4	5.22	3.77	8.93	40.31	18.46	0	11.67	30.49
OTU29	Metazoa|Cnidaria|Cnidaria_X|Hydrozoa	0	0	0	0	0	0	0	0	0	0
OTU30	Metazoa|Mollusca|Gastropoda|Heterobranchia|Bulla	0	0	0	0	207.45	0	0	0	0	0
OTU31	Metazoa|Platyhelminthes	0	0	0	0	0	0	0	0	0	0
OTU32	Metazoa|Arthropoda|Crustacea|Ostracoda	0	0	0	11.48	0	42.24	6.36	0	0	0
OTU33	Metazoa|Nemertea	0	0	0	0	0	0	0	0	0	0
OTU35	Metazoa|Nemertea	0	0	0	0	0	0	0	0	0	0
OTU36	Metazoa|Arthropoda|Chelicerata	4.76	0	0	5.45	0	3.44	0	0	0	0
OTU37	Metazoa|Annelida|Phyllodocida|Syllidae|Erinaceusyllis|Erinaceusyllis+cryptica	0	0	0	0	0	0	0	0	0	0
OTU38	Metazoa|Platyhelminthes	0	0	0	0	0	0	0	0	0	0
OTU39	Metazoa|Arthropoda|Crustacea|Maxillopoda|Copepoda	0	24.99	2.8	0	29.36	15.04	20.94	0	12.22	12.33
OTU40	Metazoa|Annelida|Capitellida|Maldanidae|Praxillella	0	0	0	0	0	0	0	0	0	0
OTU41	Metazoa|Arthropoda|Crustacea|Maxillopoda|Copepoda	59.87	0	0	0	0	0	0	0	0	0
OTU42	Metazoa|Annelida|Phyllodocida|Syllidae|Exogone|Exogone+naidina	0	0	0	0	0	0	0	0	0	0
OTU43	Metazoa|Hemichordata	0	0	0	0	0	0	0	0	0	0
OTU44	Metazoa|Cnidaria|Cnidaria_X|Hydrozoa	0	0	0	0	0	0	0	0	3.01	0
OTU45	Metazoa|Mollusca|Bivalvia|Protobranchia|Nucula	0	0	0	3.18	0	0	0	0	0	0
OTU46	Metazoa|Annelida|Capitellida|Capitellidae|Capitella	0	0	0	0	0	0	0	0	0	0
OTU47	Metazoa|Platyhelminthes	0	0	0	0	0	0	0	0	0	0
OTU48	Metazoa|Platyhelminthes	0	0	0	0	0	0	0	0	0	0
OTU49	Metazoa|Platyhelminthes	0	0	0	0	0	0	0	0	0	0
OTU51	Metazoa|Nematoda	4.03	6.79	0	6.55	9.08	19.62	13.16	0	6.08	37.77
OTU52	Metazoa|Annelida|Capitellida|Maldanidae	0	0	0	0	0	0	0	0	0	0
OTU53	Metazoa|Nematoda	8.32	10.91	0	0	0	2.69	0	0	1.77	4.31
OTU54	Metazoa|Annelida|Oligochaeta|Tubificidae	0	0	0	0	0	0	0	0	0	0
OTU55	Metazoa|Platyhelminthes	0	0	0	0	0	0	0	0	0	0
OTU56	Metazoa|Platyhelminthes	0	0	0	0	0	0	0	0	0	0
OTU58	Metazoa|Nematoda	0	6.3	3.04	1.6	19.55	2.21	0	0	0	2.01

OTU59	Metazoa|Echinodermata|Echinodermata_X|Echinodermata_XX|Amphiura	0	0	0	0	0	0	0	0	0	0
OTU60	Metazoa|Platyhelminthes	0	0	0	0	0	0	0	0	0	0
OTU61	Metazoa|Cnidaria|Cnidaria_X|Hydrozoa	0	0	0	0	0	0	0	0	0	0
OTU62	Metazoa|Platyhelminthes	0	0	0	0	0	0	0	0	0	0
OTU64	Metazoa|Arthropoda|Crustacea|Maxillopoda|Copepoda	9.1	0	12.92	0	0	0	0	0	0	0
OTU65	Metazoa|Nematoda	0	0	0	0	0	0	0	0	0	0
OTU66	Metazoa|Arthropoda|Crustacea|Maxillopoda|Copepoda	0	0	0	0	0	0	0	0	0	0
OTU68	Metazoa|Platyhelminthes	0	0	0	0	0	0	0	0	0	0
OTU69	Metazoa|Platyhelminthes	0	0	0	0	0	0	0	0	0	0
OTU70	Metazoa|Arthropoda|Crustacea|Maxillopoda|Copepoda	0	0	0	0	0	0	0	0	0	0
OTU71	Metazoa|Platyhelminthes	0	0	0	0	0	0	0	0	0	0
OTU72	Metazoa|Nematoda	0	2.92	2.42	0	0	0	0	0	2.43	6.76
OTU73	Metazoa|Nematoda	0	0	0	0	2.41	0	0	0	0	0
OTU75	Metazoa|Gastrotricha	0	0	0	0	0	0	0	0	0	0
OTU76	Metazoa|Arthropoda|Crustacea|Maxillopoda|Copepoda	0	0	0	0	0	0	0	0	0	0
OTU78	Metazoa|Mollusca|Gastropoda|Heterobranchia|Calliopaea|Calliopaea+bellula	5.84	0	0	0	0	0	0	0	0	0
OTU79	Metazoa|Annelida|Phyllodocida|Syllidae	0	0	0	0	0	0	0	0	0	0
OTU80	Metazoa|Echinodermata|Echinodermata_X|Echinodermata_XX|Leptosynapta	0	0	0	0	0	0	0	0	0	0
OTU82	Metazoa|Annelida|Capitellida|Capitellidae|Capitella	0	0	0	0	0	0	0	0	0	0
OTU84	Metazoa|Annelida|Capitellida|Capitellidae|Capitella	0	0	0	0	0	0	0	0	0	0
OTU85	Metazoa|Platyhelminthes	0	0	0	0	0	0	0	0	0	0
OTU86	Metazoa|Platyhelminthes	0	0	0	0	0	0	0	0	0	0
OTU87	Metazoa|Annelida|Terebellida|Terebellidae	0	0	0	0	0	0	0	0	0	0
OTU88	Metazoa|Platyhelminthes	0	0	0	0	0	0	0	0	0	1.67
OTU89	Metazoa|Annelida|Oligochaeta|Tubificidae	0	0	0	0	0	1.19	0	0	0	0
OTU90	Metazoa|Cnidaria|Cnidaria_X|Anthozoa|Ceriantheopsis	0	0	0	0	0	0	0	0	0	0
OTU91	Metazoa|Annelida|Capitellida|Scalibregmatidae|Scalibregma|Scalibregma+inflatum	0	0	0	0	0	0	0	0	0	0
OTU92	Metazoa|Annelida|Scolecida|Opheliidae|Ophelina	0	0	0	0	0	0	0	0	0	0
OTU93	Metazoa|Gastrotricha	0	0	0	0	0	0	0	0	0	0

OTU94	Metazoa|Platyhelminthes	0	0	0	0	0	0	0	0	0	0
OTU95	Metazoa|Arthropoda|Crustacea|Ostracoda	0	0	0	0	0	0	0	0	0	0
OTU96	Metazoa|Platyhelminthes	0	0	0	0	0	0	0	0	0	0
OTU97	Metazoa|Platyhelminthes	0	0	0	0	0	0	0	0	0	0
OTU98	Metazoa|Platyhelminthes	0	0	0	0	0	0	0	0	0	0
OTU99	Metazoa|Platyhelminthes	0	0	0	0	0	0	0	0	0	0
OTU100	Metazoa|Arthropoda|Crustacea|Ostracoda	0	0	0	0	0	0	0	0	0	0
OTU101	Metazoa|Annelida|Phyllodocida|Phyllodocidae	0	0	0	0	0	0	0	0	0	0
OTU102	Metazoa|Arthropoda|Crustacea|Maxillopoda|Copepoda	0	0	0	0	0	0	0	0	0	0
OTU103	Metazoa|Nemertea	0	0	0	0	0	0	0	0	0	0
OTU104	Metazoa|Annelida|Phyllodocida|Syllidae	0	0	0	0	0	0	0	0	0	0
OTU107	Metazoa|Arthropoda|Crustacea|Ostracoda	0	0	0	0	0	0	0	0	0	0
OTU108	Metazoa|Annelida|Spionida|Spionidae|Malacoceros|Malacoceros+fuliginosus	0	0	0	0	0	0	0	0	0	0
OTU109	Metazoa|Arthropoda|Crustacea|Maxillopoda|Copepoda	0	0	0	0	0	0	0	0	0	0
OTU110	Metazoa|Arthropoda|Crustacea|Maxillopoda|Copepoda	0	0	0	0	0	0	0	0	0	0
OTU111	Metazoa|Kinorhyncha	0	0	0	0	0	0	0	0	0	0
OTU112	Metazoa|Platyhelminthes	0	0	0	0	0	0	0	0	0	0
OTU113	Metazoa|Nematoda	0	0	0	0	0	0	0	0	0	0
OTU114	Metazoa|Nematoda	0	0	0	0	0	0	0	0	0	0
OTU115	Metazoa	0	0	0	0	0	0	0	0	0	0
OTU116	Metazoa|Platyhelminthes	0	0	0	0	0	0	0	0	0	0
OTU117	Metazoa|Nematoda	0	0	0	0	0	0	0	0	0	0
OTU118	Metazoa|Arthropoda|Crustacea|Maxillopoda|Copepoda	0	0	0	0	0	0	0	0	0	0
OTU119	Metazoa|Nematoda	0	1.05	0	0	0	0	5.85	0	0	0
OTU120	Metazoa|Platyhelminthes	0	0	0	0	0	0	0	0	0	0
OTU121	Metazoa|Nematoda	0	0	0	0	0	0	0	0	0	0
OTU122	Metazoa|Annelida|Oligochaeta|Tubificidae	0	0	0	0	0	0	0	0	0	0
OTU123	Metazoa|Mollusca|Bivalvia|Heteroconchia|Myrtea|Myrtea+spinifera	0	0	0	0	0	0	0	0	0	0
OTU124	Metazoa|Annelida|Terebellida|Pectinariidae|Pectinaria	0	0	0	0	0	0	0	0	0	0

OTU125	Metazoa|Platyhelminthes	0	0	0	0	0	0	0	0	0	0
OTU126	Metazoa|Nematoda	0	0	0	0	0	0	0	0	0	0
OTU128	Metazoa|Kinorhyncha	0	0	0	0	0	0	0	0	0	0
OTU130	Metazoa|Annelida|Phyllodocida|Phyllodocidae	0	0	0	0	0	0	0	0	0	0
OTU131	Metazoa|Annelida|Oligochaeta|Tubificidae	0	0	0	0	0	0	0	0	0	0
OTU132	Metazoa|Nematoda	0	0	0	0	0	0	0	0	0	0
OTU134	Metazoa|Annelida|Phyllodocida|Syllidae	0	0	0	0	0	0	0	0	0	0
OTU135	Metazoa|Annelida|Capitellida|Capitellidae|Capitella	0	0	0	0	0	0	0	0	0	0
OTU136	Metazoa|Nematoda	0	0	0	0	0	0	0	0	0	0
OTU137	Metazoa|Hemichordata	0	0	0	0	0	0	0	0	0	0
OTU138	Metazoa	0	0	0	0	0	0	0	0	0	0
OTU141	Metazoa|Nematoda	0	0	0	0	0	0	0	0	0	0
OTU143	Metazoa|Annelida|Capitellida|Capitellidae|Capitella	0	0	0	0	0	0	0	0	0	0
OTU144	Metazoa|Platyhelminthes	0	0	0	0	0	0	0	0	0	0
OTU145	Metazoa|Annelida|Spionida|Spionidae|Malacoceros|Malacoceros+fuliginosus	0	0	0	0	0	0	0	0	0	0
OTU146	Metazoa|Arthropoda|Crustacea|Maxillopoda|Copepoda	0	0	0	0	0	0	0	0	0	0
OTU147	Metazoa|Porifera	0	0	0	0	0	0	0	0	0	0
OTU149	Metazoa|Mollusca|Bivalvia|Heteroconchia|Macoma|Macoma+nasuta	0	0	0	0	0	0	0	0	0	0
OTU151	Metazoa|Nematoda	0	0	0	0	0	0	0	0	0	0
OTU152	Metazoa|Platyhelminthes	0	0	0	0	0	0	0	0	0	0
OTU153	Metazoa|Annelida|Phyllodocida|Phyllodocidae	0	0	0	0	0	0	0	0	0	0
OTU154	Metazoa|Annelida|Spionida|Cirratulidae	0	0	0	0	0	1.19	0	0	0	0
OTU155	Metazoa|Annelida|Spionida|Cirratulidae	0	0	0	0	0	0	0	0	0	0
OTU156	Metazoa|Arthropoda|Crustacea|Maxillopoda|Copepoda	0	0	0	0	0	0	0	0	0	0
OTU157	Metazoa|Annelida|Phyllodocida|Phyllodocidae	0	0	0	0	0	0	0	0	0	0
OTU158	Metazoa|Annelida|Phyllodocida|Phyllodocidae	0	0	0	0	0	0	0	0	0	0
OTU159	Metazoa|Rotifera	0	0	0	0	0	0	0	0	0	0
OTU160	Metazoa|Arthropoda|Crustacea|Maxillopoda|Copepoda	0	0	0	0	0	0	0	0	0	0
OTU161	Metazoa|Platyhelminthes	0	0	0	0	0	0	0	0	0	0

OTU162	Metazoa|Annelida|Capitellida|Capitellidae|Capitella	0	0	0	0	0	0	0	0	0	0
OTU163	Metazoa|Nematoda	0	0	0	0	0	0	0	0	0	0
OTU164	Metazoa|Nematoda	0	0	0	0	0	0	0	0	0	0
OTU165	Metazoa|Nematoda	0	0	0	0	0	0	0	0	0	0
OTU167	Metazoa|Nematoda	0	0	0	0	0	0	1.52	0	0	0
OTU168	Metazoa|Cnidaria|Cnidaria_X|Hydrozoa	0	0	0	0	0	0	0	0	0	0
OTU169	Metazoa|Arthropoda|Crustacea|Maxillopoda|Copepoda	0	0	0	0	0	0	0	0	0	0
OTU170	Metazoa|Gastrotricha	0	0	0	0	0	0	0	0	0	0
OTU171	Metazoa|Annelida|Phyllodocida|Syllidae	0	0	0	0	0	0	2.13	0	0	0
OTU173	Metazoa|Nematoda	0	0	0	0	0	0	0	0	0	0
OTU174	Metazoa|Arthropoda|Crustacea|Maxillopoda|Copepoda	0	0	0	0	0	0	0	0	0	0
OTU175	Metazoa|Arthropoda|Crustacea|Maxillopoda|Copepoda	0	0	0	0	0	0	0	0	0	0
OTU176	Metazoa|Platyhelminthes	0	0	0	0	0	0	0	0	0	0
OTU178	Metazoa|Nematoda	0	0	0	0	0	0	0	0	0	0
OTU180	Metazoa|Nematoda	0	0	0	0	0	0	0	0	0	1.7
OTU183	Metazoa|Annelida|Oligochaeta|Tubificidae	0	0	0	0	0	0	0	0	0	0
OTU184	Metazoa|Nematoda	0	0	0	0	0	0	0	0	0	0
OTU186	Metazoa|Platyhelminthes	0	0	0	0	0	0	0	0	0	0
OTU188	Metazoa|Annelida|Terebellida|Ampharetidae	0	0	0	0	0	0	0	0	0	0
OTU190	Metazoa|Annelida|Terebellida|Terebellidae	0	0	0	0	0	0	0	0	0	0
OTU191	Metazoa|Annelida|Phyllodocida|Syllidae	0	0	0	0	0	0	0	0	0	0
OTU192	Metazoa|Annelida|Spionida|Cirratulidae	0	0	0	0	0	0	0	0	0	0

Supplementary Table 7. continued

OTU	assign	DNA_6_1	DNA_6_2	DNA_6_3	DNA_6_4	DNA_6_5	DNA_7_1	DNA_7_2	DNA_7_3	DNA_7_4	DNA_7_5
OTU1	Metazoa|Annelida|Capitellida|Capitellidae|Capitella	2807.39	2780.39	3299.36	2096.31	2163.16	0	0	0	0	0
OTU2	Metazoa|Annelida|Oligochaeta|Tubificidae	238.28	13.48	131.97	378.68	510.81	0	0	0	0	0
OTU4	Metazoa|Annelida|Phyllodocida|Phyllodocidae|Phyllodoce|Phyllodoce+maculata	0	7.67	0	148.7	114.44	0	3569.67	0	0	0
OTU5	Metazoa|Annelida|Spionida|Spionidae|Malacoceros|Malacoceros+fuliginosus	0	0	0	0	0	0	0	0	0	0
OTU6	Metazoa|Annelida|Spionida|Cirratulidae	0	0	0	0	0	0	0	0	0	0
OTU7	Metazoa|Platyhelminthes	23.64	216.41	182.76	31.08	601.8	46.55	16.72	3270.95	0	77.34
OTU8	Metazoa|Annelida|Phyllodocida|Syllidae|Salvatoria	942.58	961.39	3.83	1281.81	57.04	0	0	0	4.11	498.7
OTU9	Metazoa|Annelida	1.01	1.17	55.64	69.29	0	486.21	404.19	0	1238.57	871.19
OTU10	Metazoa|Nematoda	0	0	0	0	0	0	0	0	0	0
OTU11	Metazoa|Nematoda	0	0	0	2.39	0	0	0	0	0	0
OTU12	Metazoa|Nematoda	19.69	25.65	137.13	47.14	94.97	0	0	0	0	4.17
OTU13	Metazoa|Cnidaria|Cnidaria_X|Anthozoa	0	0	0	0	0	0	0	0	0	0
OTU14	Metazoa|Nemertea	0	0	0	0	0	0	28.72	0	0	0
OTU15	Metazoa|Mollusca|Bivalvia|Heteroconchia|Abra	0	0	0	0	0	0	0	0	6.3	38.99
OTU16	Metazoa|Platyhelminthes	0	0	0	0	0	470.94	7.02	26.64	43.7	139.46
OTU17	Metazoa|Cnidaria|Cnidaria_X|Hydrozoa	0	0	0	1.44	0	0	0	14.47	14.02	218.58
OTU18	Metazoa|Platyhelminthes	5.55	4.29	90.35	20.5	53.18	141.97	0	9.14	0	0
OTU19	Metazoa|Arthropoda|Crustacea|Ostracoda	0	0	0	0	0	104.68	0	88.11	22.35	15.6
OTU20	Metazoa|Nematoda	4.89	2.52	42.03	5.16	29.78	0	0	0	0	0
OTU21	Metazoa|Annelida|Terebellida|Ampharetidae	0	0	0	0	0	0	0	0	2390.04	0
OTU22	Metazoa|Annelida|Phyllodocida|Syllidae|Exogone|Exogone+naidina	0	0	0	0	0	0	0	0	0	0
OTU23	Metazoa|Platyhelminthes	0	0	0	0	0	443.59	8.12	238.09	25.4	292.63
OTU24	Metazoa|Platyhelminthes	0	0	0	0	0	0	0	0	0	0
OTU25	Metazoa|Mollusca|Bivalvia|Heteroconchia|Corbula	0	0	0	0	0	0	0	0	0	0
OTU26	Metazoa|Annelida|Capitellida|Capitellidae|Heteromastus	2.83	0	4.17	5.21	17.22	0	0	0	8.11	0

OTU27	Metazoa|Arthropoda|Crustacea|Maxillopoda|Copepoda	0	0	0	0	0	12.54	1.54	8.67	5.84	38.96
OTU28	Metazoa|Nematoda	0	0	0	0	3.69	0	0	0	0	0
OTU29	Metazoa|Cnidaria|Cnidaria_X|Hydrozoa	0	0	0	0	0	967.11	0	0	0	0
OTU30	Metazoa|Mollusca|Gastropoda|Heterobranchia|Bulla	0	0	0	0	0	544.66	0	0	0	0
OTU31	Metazoa|Platyhelminthes	0	0	0	0	0	116.67	2.69	32.21	21.49	0
OTU32	Metazoa|Arthropoda|Crustacea|Ostracoda	0	0	26.54	0	0	0	0	0	0	0
OTU33	Metazoa|Nemertea	0	0	0	0	0	0	0	0	0	0
OTU35	Metazoa|Nemertea	0	0	0	0	0	0	0	39.37	0	0
OTU36	Metazoa|Arthropoda|Chelicerata	0	0	0	0	0	0	0	0	51.14	529.5
OTU37	Metazoa|Annelida|Phyllodocida|Syllidae|Erinaceusyllis|Erinaceusyllis+cryptica	0	2.22	0	0	420.16	0	0	0	0	7.79
OTU38	Metazoa|Platyhelminthes	49.78	0	0	0	0	0	0	0	0	0
OTU39	Metazoa|Arthropoda|Crustacea|Maxillopoda|Copepoda	0	0	1.06	1.11	0	0	0	0	0	272.28
OTU40	Metazoa|Annelida|Capitellida|Maldanidae|Praxillella	0	0	0	0	0	0	0	0	0	0
OTU41	Metazoa|Arthropoda|Crustacea|Maxillopoda|Copepoda	0	0	0	0	0	0	0	0	0	0
OTU42	Metazoa|Annelida|Phyllodocida|Syllidae|Exogone|Exogone+naidina	0	0	108.45	0	27.92	0	0	0	0	0
OTU43	Metazoa|Hemichordata	0	0	0	0	0	0	34.71	240.66	0	0
OTU44	Metazoa|Cnidaria|Cnidaria_X|Hydrozoa	0	0	0	0	1.38	0	0	0	0	0
OTU45	Metazoa|Mollusca|Bivalvia|Protobranchia|Nucula	0	0	0	0	0	26.03	0	0	0	0
OTU46	Metazoa|Annelida|Capitellida|Capitellidae|Capitella	0	80.84	0	0	0	0	0	0	0	0
OTU47	Metazoa|Platyhelminthes	0	0	0	0	0	84.53	0	0	0	355.14
OTU48	Metazoa|Platyhelminthes	0	0	0	0	0	29.32	0	0	0	22.1
OTU49	Metazoa|Platyhelminthes	0	0	0	0	0	0	0	0	0	0
OTU51	Metazoa|Nematoda	0	0	3.13	2.15	0	0	0	0	0	0
OTU52	Metazoa|Annelida|Capitellida|Maldanidae	0	0	0	0	0	0	0	0	0	145.3
OTU53	Metazoa|Nematoda	0	0	13.29	0	5.16	0	0	0	0	0
OTU54	Metazoa|Annelida|Oligochaeta|Tubificidae	0	0	0	0	0	0	0	0	0	0
OTU55	Metazoa|Platyhelminthes	0	0	0	0	0	165.04	0	19.76	8.37	21.08
OTU56	Metazoa|Platyhelminthes	0	0	0	0	0	27.17	0	17.95	0	155.79
OTU58	Metazoa|Nematoda	0	0	0	0	1.29	0	0	0	0	21.52

OTU59	Metazoa|Echinodermata|Echinodermata_X|Echinodermata_XX|Amphiura	0	0	0	0	0	0	0	0	177.01	0
OTU60	Metazoa|Platyhelminthes	0	0	0	0	0	0	0	0	0	0
OTU61	Metazoa|Cnidaria|Cnidaria_X|Hydrozoa	0	0	0	0	0	0	0	0	0	0
OTU62	Metazoa|Platyhelminthes	0	0	0	0	0	0	0	0	0	0
OTU64	Metazoa|Arthropoda|Crustacea|Maxillopoda|Copepoda	0	0	0	0	0	0	0	0	0	0
OTU65	Metazoa|Nematoda	0	0	0	0	0	0	0	0	0	174.08
OTU66	Metazoa|Arthropoda|Crustacea|Maxillopoda|Copepoda	0	0	0	0	0	14.6	1.2	1.54	4.25	0
OTU68	Metazoa|Platyhelminthes	0	0	0	0	0	0	0	0	0	0
OTU69	Metazoa|Platyhelminthes	0	0	0	0	0	0	0	0	0	0
OTU70	Metazoa|Arthropoda|Crustacea|Maxillopoda|Copepoda	0	0	0	0	0	0	0	0	0	0
OTU71	Metazoa|Platyhelminthes	0	0	0	0	0	0	0	0	0	0
OTU72	Metazoa|Nematoda	0	0	0	0	0	0	0	0	0	0
OTU73	Metazoa|Nematoda	0	0	0	0	0	0	0	0	0	0
OTU75	Metazoa|Gastrotricha	0	0	0	0	0	5.88	0	0	0	11.78
OTU76	Metazoa|Arthropoda|Crustacea|Maxillopoda|Copepoda	0	0	0	0	0	5.98	0	0	0	25.72
OTU78	Metazoa|Mollusca|Gastropoda|Heterobranchia|Calliopaea|Calliopaea+bellula	0	0	1.52	0	0	0	0	0	0	0
OTU79	Metazoa|Annelida|Phyllodocida|Syllidae	4.86	0	0	2.03	0	0	0	0	0	0
OTU80	Metazoa|Echinodermata|Echinodermata_X|Echinodermata_XX|Leptosynapta	0	0	0	0	0	0	0	71.1	0	0
OTU82	Metazoa|Annelida|Capitellida|Capitellidae|Capitella	0	0	0	0	0	0	0	0	0	0
OTU84	Metazoa|Annelida|Capitellida|Capitellidae|Capitella	0	0	0	0	0	0	0	9.32	0	0
OTU85	Metazoa|Platyhelminthes	0	0	0	0	0	0	0	0	0	0
OTU86	Metazoa|Platyhelminthes	0	0	0	0	0	0	0	0	0	0
OTU87	Metazoa|Annelida|Terebellida|Terebellidae	0	0	0	0	0	0	0	0	0	0
OTU88	Metazoa|Platyhelminthes	0	0	0	0	0	0	0	0	0	0
OTU89	Metazoa|Annelida|Oligochaeta|Tubificidae	0	0	0	0	0	0	0	0	0	0
OTU90	Metazoa|Cnidaria|Cnidaria_X|Anthozoa|Ceriantheopsis	0	0	0	0	0	0	0	0	0	0
OTU91	Metazoa|Annelida|Capitellida|Scalibregmatidae|Scalibregma|Scalibregma+inflatum	0	0	0	0	0	0	0	0	0	0
OTU92	Metazoa|Annelida|Scolecida|Opheliidae|Ophelina	0	0	0	0	0	0	0	0	0	0
OTU93	Metazoa|Gastrotricha	0	0	0	0	0	21.23	0	0	0	24.52

OTU94	Metazoa|Platyhelminthes	0	0	0	0	0	0	0	0	0	0
OTU95	Metazoa|Arthropoda|Crustacea|Ostracoda	0	0	0	0	0	0	0	0	0	0
OTU96	Metazoa|Platyhelminthes	0	0	0	0	0	51.51	0	0	28.87	0
OTU97	Metazoa|Platyhelminthes	0	0	0	0	0	0	0	0	0	0
OTU98	Metazoa|Platyhelminthes	0	0	0	0	0	93.93	0	0	0	0
OTU99	Metazoa|Platyhelminthes	0	0	0	0	0	0	0	0	0	0
OTU100	Metazoa|Arthropoda|Crustacea|Ostracoda	0	0	0	0	0	69.39	0	0	0	0
OTU101	Metazoa|Annelida|Phyllodocida|Phyllodocidae	0	0	0	0	0	0	16.49	0	0	0
OTU102	Metazoa|Arthropoda|Crustacea|Maxillopoda|Copepoda	0	0	0	0	0	0	0	0	0	0
OTU103	Metazoa|Nemertea	0	0	0	0	0	0	5.79	0	0	0
OTU104	Metazoa|Annelida|Phyllodocida|Syllidae	0	0	0	0	0	0	0	0	4.07	0
OTU107	Metazoa|Arthropoda|Crustacea|Ostracoda	0	0	0	0	0	0	0	0	0	0
OTU108	Metazoa|Annelida|Spionida|Spionidae|Malacoceros|Malacoceros+fuliginosus	0	0	0	0	0	0	0	0	0	0
OTU109	Metazoa|Arthropoda|Crustacea|Maxillopoda|Copepoda	0	0	0	0	0	0	0	0	0	60.81
OTU110	Metazoa|Arthropoda|Crustacea|Maxillopoda|Copepoda	0	0	0	6.72	0	0	0	0	0	0
OTU111	Metazoa|Kinorhyncha	0	0	0	0	0	0	0	0	0	0
OTU112	Metazoa|Platyhelminthes	0	0	0	0	0	0	0	0	4.02	0
OTU113	Metazoa|Nematoda	0	0	0	0	0	15.12	0	0	0	15.24
OTU114	Metazoa|Nematoda	0	0	0	0	0	0	0	0	14.42	0
OTU115	Metazoa	0	0	0	0	0	43.15	0	1.64	0	0
OTU116	Metazoa|Platyhelminthes	0	0	0	0	0	0	0	0	0	0
OTU117	Metazoa|Nematoda	0	0	0	0	0	0	0	0	0	0
OTU118	Metazoa|Arthropoda|Crustacea|Maxillopoda|Copepoda	0	0	0	0	0	23.39	0	1.7	0	13.75
OTU119	Metazoa|Nematoda	0	0	0	0	0	0	0	0	0	0
OTU120	Metazoa|Platyhelminthes	0	0	0	0	0	0	0	0	0	0
OTU121	Metazoa|Nematoda	0	0	0	0	0	0	0	0	0	0
OTU122	Metazoa|Annelida|Oligochaeta|Tubificidae	0	0	0	0	0	0	0	0	0	0
OTU123	Metazoa|Mollusca|Bivalvia|Heteroconchia|Myrtea|Myrtea+spinifera	0	0	0	0	0	0	0	0	0	0
OTU124	Metazoa|Annelida|Terebellida|Pectinariidae|Pectinaria	0	0	0	0	0	0	0	0	0	0

OTU125	Metazoa|Platyhelminthes	0	0	0	0	0	0	0	0	0	0
OTU126	Metazoa|Nematoda	0	2.73	0	0	0	0	0	0	0	0
OTU128	Metazoa|Kinorhyncha	0	0	0	0	0	0	0	0	0	29.68
OTU130	Metazoa|Annelida|Phyllodocida|Phyllodocidae	0	0	0	0	0	0	1.01	0	0	0
OTU131	Metazoa|Annelida|Oligochaeta|Tubificidae	0	0	0	0	0	0	0	0	0	0
OTU132	Metazoa|Nematoda	0	0	0	0	0	0	0	0	0	0
OTU134	Metazoa|Annelida|Phyllodocida|Syllidae	0	2.32	0	0	0	0	0	0	0	0
OTU135	Metazoa|Annelida|Capitellida|Capitellidae|Capitella	0	0	0	0	0	0	0	0	0	0
OTU136	Metazoa|Nematoda	0	0	0	0	0	0	0	0	0	0
OTU137	Metazoa|Hemichordata	0	0	0	0	0	0	0	0	0	0
OTU138	Metazoa	0	0	0	0	0	39.08	0	0	0	0
OTU141	Metazoa|Nematoda	0	0	0	0	0	0	0	0	0	0
OTU143	Metazoa|Annelida|Capitellida|Capitellidae|Capitella	0	0	0	0	0	0	0	0	0	0
OTU144	Metazoa|Platyhelminthes	0	0	0	0	0	0	0	4.18	0	0
OTU145	Metazoa|Annelida|Spionida|Spionidae|Malacoceros|Malacoceros+fuliginosus	0	0	0	0	0	0	0	0	0	0
OTU146	Metazoa|Arthropoda|Crustacea|Maxillopoda|Copepoda	0	0	0	0	0	0	0	0	0	0
OTU147	Metazoa|Porifera	0	0	0	0	0	27.24	0	0	0	0
OTU149	Metazoa|Mollusca|Bivalvia|Heteroconchia|Macoma|Macoma+nasuta	0	0	0	0	0	0	0	0	0	0
OTU151	Metazoa|Nematoda	0	0	0	0	0	0	0	0	0	0
OTU152	Metazoa|Platyhelminthes	0	0	0	0	0	0	0	0	0	0
OTU153	Metazoa|Annelida|Phyllodocida|Phyllodocidae	0	0	0	0	0	0	0	0	0	0
OTU154	Metazoa|Annelida|Spionida|Cirratulidae	0	0	0	0	0	0	0	0	0	0
OTU155	Metazoa|Annelida|Spionida|Cirratulidae	0	0	0	0	0	0	0	0	17.24	0
OTU156	Metazoa|Arthropoda|Crustacea|Maxillopoda|Copepoda	0	0	0	0	0	0	0	0	0	0
OTU157	Metazoa|Annelida|Phyllodocida|Phyllodocidae	0	0	0	0	0	0	0	0	0	0
OTU158	Metazoa|Annelida|Phyllodocida|Phyllodocidae	0	0	0	0	0	0	3.26	0	0	0
OTU159	Metazoa|Rotifera	0	0	0	0	0	0	0	0	0	0
OTU160	Metazoa|Arthropoda|Crustacea|Maxillopoda|Copepoda	0	0	0	0	0	0	0	0	0	0
OTU161	Metazoa|Platyhelminthes	0	0	0	0	0	0	0	3.26	0	0

OTU162	Metazoa|Annelida|Capitellida|Capitellidae|Capitella	0	0	0	0	0	0	0	0	0	0
OTU163	Metazoa|Nematoda	0	0	0	0	0	0	0	0	0	9.99
OTU164	Metazoa|Nematoda	0	0	0	0	0	0	0	0	0	0
OTU165	Metazoa|Nematoda	0	0	0	0	0	0	0	0	0	10.31
OTU167	Metazoa|Nematoda	0	0	0	0	0	0	0	0	0	0
OTU168	Metazoa|Cnidaria|Cnidaria_X|Hydrozoa	0	0	0	0	0	0	0	0	0	0
OTU169	Metazoa|Arthropoda|Crustacea|Maxillopoda|Copepoda	0	0	0	0	0	5.79	0	0	0	0
OTU170	Metazoa|Gastrotricha	0	0	0	0	0	0	0	3.24	0	0
OTU171	Metazoa|Annelida|Phyllodocida|Syllidae	0	0	0	0	0	0	0	0	0	0
OTU173	Metazoa|Nematoda	0	0	0	0	0	0	0	0	0	0
OTU174	Metazoa|Arthropoda|Crustacea|Maxillopoda|Copepoda	0	0	0	0	0	12.86	0	0	0	0
OTU175	Metazoa|Arthropoda|Crustacea|Maxillopoda|Copepoda	0	0	0	0	0	0	0	0	0	0
OTU176	Metazoa|Platyhelminthes	0	0	0	0	0	0	0	0	8.43	0
OTU178	Metazoa|Nematoda	0	0	0	0	0	0	0	0	0	0
OTU180	Metazoa|Nematoda	0	0	0	0	0	0	0	0	0	0
OTU183	Metazoa|Annelida|Oligochaeta|Tubificidae	0	0	0	0	0	0	0	0	0	0
OTU184	Metazoa|Nematoda	0	0	0	0	0	0	0	0	0	0
OTU186	Metazoa|Platyhelminthes	0	0	0	0	0	5.84	0	0	0	0
OTU188	Metazoa|Annelida|Terebellida|Ampharetidae	0	0	0	0	0	0	0	0	4.25	0
OTU190	Metazoa|Annelida|Terebellida|Terebellidae	0	0	0	0	0	0	0	0	0	0
OTU191	Metazoa|Annelida|Phyllodocida|Syllidae	0	0	0	0	0	0	0	0	0	0
OTU192	Metazoa|Annelida|Spionida|Cirratulidae	0	0	0	0	0	0	0	0	0	0

Supplementary Table 7. continued

OTU	assign	DNA_8_1	DNA_8_2	DNA_8_3	DNA_8_4	DNA_8_5	DNA_9_1	DNA_9_2	DNA_9_3	DNA_9_4	DNA_9_5
OTU1	Metazoa|Annelida|Capitellida|Capitellidae|Capitella	0	3.8	0	0	760.88	0	0	0	0	0
OTU2	Metazoa|Annelida|Oligochaeta|Tubificidae	0	0	0	0	0	0	0	0	0	0
OTU4	Metazoa|Annelida|Phyllodocida|Phyllodocidae|Phyllodoce|Phyllodoce+maculata	0	12.39	0	3635.72	1696.01	0	0	0	0	0
OTU5	Metazoa|Annelida|Spionida|Spionidae|Malacoceros|Malacoceros+fuliginosus	0	0	0	0	0	0	0	0	0	0
OTU6	Metazoa|Annelida|Spionida|Cirratulidae	0	0	0	0	0	0	0	0	0	0
OTU7	Metazoa|Platyhelminthes	1190.77	0	1166.86	63.83	1324.94	0	0	1463.03	0	2540.07
OTU8	Metazoa|Annelida|Phyllodocida|Syllidae|Salvatoria	0	0	16.29	0	36.66	609.39	22.83	178.81	0	188.6
OTU9	Metazoa|Annelida	2.22	3121.52	0	0	0	0	0	0	0	0
OTU10	Metazoa|Nematoda	0	0	0	0	0	0	0	0	0	0
OTU11	Metazoa|Nematoda	0	0	0	0	0	0	0	0	0	0
OTU12	Metazoa|Nematoda	0	0	0	0	0	0	0	0	0	0
OTU13	Metazoa|Cnidaria|Cnidaria_X|Anthozoa	0	0	0	0	0	0	0	0	0	0
OTU14	Metazoa|Nemertea	0	0	0	227.91	0	0	0	0	0	0
OTU15	Metazoa|Mollusca|Bivalvia|Heteroconchia|Abra	0	0	0	0	0	0	0	209.74	0	0
OTU16	Metazoa|Platyhelminthes	520.47	375.23	72.49	20.73	6.25	0	0	0	112.56	0
OTU17	Metazoa|Cnidaria|Cnidaria_X|Hydrozoa	105	0	145.45	0	65.27	102.68	781.14	0	0	0
OTU18	Metazoa|Platyhelminthes	0	0	48.86	0	0	160.18	0	0	16.17	0
OTU19	Metazoa|Arthropoda|Crustacea|Ostracoda	274.29	125.64	0	14.79	75.79	0	768.2	0	189.52	682.25
OTU20	Metazoa|Nematoda	0	0	0	0	0	0	0	0	0	0
OTU21	Metazoa|Annelida|Terebellida|Ampharetidae	0	0	0	0	0	0	0	0	0	0
OTU22	Metazoa|Annelida|Phyllodocida|Syllidae|Exogone|Exogone+naidina	0	0	0	0	0	0	0	0	0	0
OTU23	Metazoa|Platyhelminthes	462.73	0	229.87	5.14	5.01	0	522.9	897.08	373.18	114.57
OTU24	Metazoa|Platyhelminthes	0	0	0	0	0	0	0	0	0	0
OTU25	Metazoa|Mollusca|Bivalvia|Heteroconchia|Corbula	0	0	0	0	0	0	0	0	0	0
OTU26	Metazoa|Annelida|Capitellida|Capitellidae|Heteromastus	0	0	198.03	0	17.27	0	0	0	0	0
OTU27	Metazoa|Arthropoda|Crustacea|Maxillopoda|Copepoda	287.7	0	115.16	16.02	11.99	101.96	53.07	134.63	218.12	78.5

OTU28	Metazoa|Nematoda	0	0	0	0	0	0	0	0	0	0
OTU29	Metazoa|Cnidaria|Cnidaria_X|Hydrozoa	0	0	821.31	0	0	0	0	0	0	0
OTU30	Metazoa|Mollusca|Gastropoda|Heterobranchia|Bulla	0	0	65.3	0	0	0	0	0	0	0
OTU31	Metazoa|Platyhelminthes	0	342.34	252.95	36.72	9.23	108.44	8.97	326.43	111.47	0
OTU32	Metazoa|Arthropoda|Crustacea|Ostracoda	0	0	0	0	0	0	1467.79	0	0	0
OTU33	Metazoa|Nemertea	0	0	0	0	0	0	0	0	1844.64	0
OTU35	Metazoa|Nemertea	0	0	0	3.75	0	0	0	0	0	0
OTU36	Metazoa|Arthropoda|Chelicerata	218.6	0	0	0	10.46	42.55	108.35	0	0	160.11
OTU37	Metazoa|Annelida|Phyllodocida|Syllidae|Erinaceusyllis|Erinaceusyllis+cryptica	0	0	24.98	0	0	0	0	0	0	0
OTU38	Metazoa|Platyhelminthes	0	0	0	0	0	0	0	0	0	0
OTU39	Metazoa|Arthropoda|Crustacea|Maxillopoda|Copepoda	30.3	0	0	3.24	1.17	12.05	33.57	34.97	0	0
OTU40	Metazoa|Annelida|Capitellida|Maldanidae|Praxillella	0	0	0	0	0	1023.75	0	0	0	0
OTU41	Metazoa|Arthropoda|Crustacea|Maxillopoda|Copepoda	0	0	0	0	0	0	0	0	0	0
OTU42	Metazoa|Annelida|Phyllodocida|Syllidae|Exogone|Exogone+naidina	0	0	0	0	0	0	0	0	0	0
OTU43	Metazoa|Hemichordata	0	0	0	0	0	0	11.51	30.65	16.16	0
OTU44	Metazoa|Cnidaria|Cnidaria_X|Hydrozoa	0	0	0	0	0	0	15.75	0	0	0
OTU45	Metazoa|Mollusca|Bivalvia|Protobranchia|Nucula	0	0	0	33.33	2.34	0	0	45.28	0	0
OTU46	Metazoa|Annelida|Capitellida|Capitellidae|Capitella	0	0	0	0	0	0	0	0	0	0
OTU47	Metazoa|Platyhelminthes	216.91	0	0	0	0	83.65	0	0	0	0
OTU48	Metazoa|Platyhelminthes	32.78	47.37	435.58	12.72	35.39	0	49.78	0	85.29	0
OTU49	Metazoa|Platyhelminthes	0	0	0	0	0	0	0	0	0	0
OTU51	Metazoa|Nematoda	0	0	0	0	0	0	0	0	0	0
OTU52	Metazoa|Annelida|Capitellida|Maldanidae	0	0	0	0	0	343.3	0	0	0	0
OTU53	Metazoa|Nematoda	0	0	0	0	0	0	0	0	0	0
OTU54	Metazoa|Annelida|Oligochaeta|Tubificidae	0	0	0	0	0	0	0	0	0	0
OTU55	Metazoa|Platyhelminthes	0	3.83	49.72	3.86	10.18	0	4.6	102.77	0	68.69
OTU56	Metazoa|Platyhelminthes	0	0	0	0	0	0	0	0	452.18	0
OTU58	Metazoa|Nematoda	8.57	0	0	0	0	0	0	0	0	0
OTU59	Metazoa|Echinodermata|Echinodermata_X|Echinodermata_XX|Amphiura	0	0	0	0	0	0	0	0	0	0

OTU60	Metazoa|Platyhelminthes	291.57	0	0	0	0	0	0	0	0	0
OTU61	Metazoa|Cnidaria|Cnidaria_X|Hydrozoa	183.55	0	0	0	0	0	0	0	0	0
OTU62	Metazoa|Platyhelminthes	0	0	0	0	0	0	0	0	0	0
OTU64	Metazoa|Arthropoda|Crustacea|Maxillopoda|Copepoda	0	0	0	0	0	0	0	0	0	0
OTU65	Metazoa|Nematoda	0	0	0	0	0	0	0	0	0	0
OTU66	Metazoa|Arthropoda|Crustacea|Maxillopoda|Copepoda	56.45	0	48.66	2.1	1.5	106.53	55.93	0	0	0
OTU68	Metazoa|Platyhelminthes	0	0	24.55	3.02	0	0	0	0	0	0
OTU69	Metazoa|Platyhelminthes	0	0	0	0	0	0	0	0	0	0
OTU70	Metazoa|Arthropoda|Crustacea|Maxillopoda|Copepoda	0	0	0	2.58	0	11.83	98.55	40.42	80.09	66.62
OTU71	Metazoa|Platyhelminthes	0	0	0	0	0	585.11	0	0	0	0
OTU72	Metazoa|Nematoda	0	0	0	0	0	0	0	0	0	0
OTU73	Metazoa|Nematoda	0	0	0	0	0	0	0	0	0	0
OTU75	Metazoa|Gastrotricha	11	0	0	0	0	54.54	0	19.36	15.98	11.73
OTU76	Metazoa|Arthropoda|Crustacea|Maxillopoda|Copepoda	55.02	0	0	0	0	90.01	25.06	0	47.99	0
OTU78	Metazoa|Mollusca|Gastropoda|Heterobranchia|Calliopaea|Calliopaea+bellula	0	0	0	0	0	0	0	0	0	0
OTU79	Metazoa|Annelida|Phyllodocida|Syllidae	0	0	0	0	0	0	0	0	0	0
OTU80	Metazoa|Echinodermata|Echinodermata_X|Echinodermata_XX|Leptosynapta	0	0	0	0	0	0	0	0	0	0
OTU82	Metazoa|Annelida|Capitellida|Capitellidae|Capitella	0	0	0	0	0	0	0	0	0	0
OTU84	Metazoa|Annelida|Capitellida|Capitellidae|Capitella	47.21	69.88	0	0	0	0	0	0	0	0
OTU85	Metazoa|Platyhelminthes	0	0	0	3.59	19.94	0	0	0	0	0
OTU86	Metazoa|Platyhelminthes	0	0	0	0	0	0	0	0	0	0
OTU87	Metazoa|Annelida|Terebellida|Terebellidae	0	0	0	0	0	0	0	0	0	0
OTU88	Metazoa|Platyhelminthes	0	0	0	0	0	0	0	0	0	0
OTU89	Metazoa|Annelida|Oligochaeta|Tubificidae	0	0	0	0	0	0	0	0	0	0
OTU90	Metazoa|Cnidaria|Cnidaria_X|Anthozoa|Ceriantheopsis	0	0	0	0	0	0	0	0	273.12	0
OTU91	Metazoa|Annelida|Capitellida|Scalibregmatidae|Scalibregma|Scalibregma+inflatum	0	0	0	0	0	0	12.89	0	0	0
OTU92	Metazoa|Annelida|Scolecida|Opheliidae|Ophelina	0	0	0	0	0	296.29	0	0	0	0
OTU93	Metazoa|Gastrotricha	29.27	0	0	0	0	0	0	0	0	7.61
OTU94	Metazoa|Platyhelminthes	0	0	320.31	0	0	0	0	0	0	0

OTU95	Metazoa|Arthropoda|Crustacea|Ostracoda	0	0	0	0	0	18.19	0	173.72	0	0
OTU96	Metazoa|Platyhelminthes	0	0	0	0	0	42.81	0	0	0	0
OTU97	Metazoa|Platyhelminthes	0	0	0	0	0	0	0	208.57	0	0
OTU98	Metazoa|Platyhelminthes	0	0	0	0	0	0	0	0	0	0
OTU99	Metazoa|Platyhelminthes	0	0	0	0	0	0	0	0	148.64	0
OTU100	Metazoa|Arthropoda|Crustacea|Ostracoda	0	0	0	2.29	0	0	0	0	0	0
OTU101	Metazoa|Annelida|Phyllodocida|Phyllodocidae	0	0	0	0	0	0	0	0	0	0
OTU102	Metazoa|Arthropoda|Crustacea|Maxillopoda|Copepoda	0	0	0	1.9	0	41.4	18.03	19.27	0	20.36
OTU103	Metazoa|Nemertea	0	0	0	2.51	0	0	0	0	0	0
OTU104	Metazoa|Annelida|Phyllodocida|Syllidae	0	0	0	0	0	48.19	0	0	0	0
OTU107	Metazoa|Arthropoda|Crustacea|Ostracoda	0	0	16.35	0	0	30.55	26.83	0	69.41	0
OTU108	Metazoa|Annelida|Spionida|Spionidae|Malacoceros|Malacoceros+fuliginosus	0	0	0	0	0	0	0	0	0	0
OTU109	Metazoa|Arthropoda|Crustacea|Maxillopoda|Copepoda	0	0	0	0	0	0	0	0	0	0
OTU110	Metazoa|Arthropoda|Crustacea|Maxillopoda|Copepoda	0	0	0	0	0	0	0	0	21.09	0
OTU111	Metazoa|Kinorhyncha	28.92	0	0	0	0	11.97	0	62.85	10.58	0
OTU112	Metazoa|Platyhelminthes	0	0	49.28	0	0	0	0	89.49	0	0
OTU113	Metazoa|Nematoda	8.67	0	0	0	0	0	0	0	0	0
OTU114	Metazoa|Nematoda	0	0	0	0	0	0	0	0	0	0
OTU115	Metazoa	0	0	0	0	0	0	0	0	0	0
OTU116	Metazoa|Platyhelminthes	0	0	0	0	0	140.3	0	0	0	0
OTU117	Metazoa|Nematoda	0	0	0	0	0	0	9.59	0	0	0
OTU118	Metazoa|Arthropoda|Crustacea|Maxillopoda|Copepoda	0	0	0	0	0	0	0	0	0	0
OTU119	Metazoa|Nematoda	0	0	0	0	0	0	0	0	0	0
OTU120	Metazoa|Platyhelminthes	0	0	0	0	0	0	0	0	0	47.5
OTU121	Metazoa|Nematoda	0	0	0	1.02	0	0	0	0	0	0
OTU122	Metazoa|Annelida|Oligochaeta|Tubificidae	0	0	0	0	0	0	0	0	0	0
OTU123	Metazoa|Mollusca|Bivalvia|Heteroconchia|Myrtea|Myrtea+spinifera	0	0	0	2.75	0	0	0	0	0	0
OTU124	Metazoa|Annelida|Terebellida|Pectinariidae|Pectinaria	0	0	0	0	0	0	0	0	0	55.44
OTU125	Metazoa|Platyhelminthes	0	0	0	0	1.52	0	0	0	0	59.95

OTU126	Metazoa|Nematoda	0	0	0	0	0	0	0	0	0	0
OTU128	Metazoa|Kinorhyncha	0	0	0	0	0	0	0	0	0	0
OTU130	Metazoa|Annelida|Phyllodocida|Phyllodocidae	0	0	0	0	2.44	0	0	0	0	0
OTU131	Metazoa|Annelida|Oligochaeta|Tubificidae	0	0	0	0	0	0	0	0	0	0
OTU132	Metazoa|Nematoda	6.75	0	0	0	0	0	0	0	0	0
OTU134	Metazoa|Annelida|Phyllodocida|Syllidae	0	0	0	0	0	0	0	0	0	0
OTU135	Metazoa|Annelida|Capitellida|Capitellidae|Capitella	0	0	0	0	0	0	0	0	0	0
OTU136	Metazoa|Nematoda	3.96	0	0	0	0	0	0	0	15.81	0
OTU137	Metazoa|Hemichordata	0	0	0	0	0	0	0	64.93	0	0
OTU138	Metazoa	0	0	0	0	0	0	0	0	0	0
OTU141	Metazoa|Nematoda	0	0	0	0	0	0	0	0	0	0
OTU143	Metazoa|Annelida|Capitellida|Capitellidae|Capitella	0	0	0	0	0	0	0	0	0	0
OTU144	Metazoa|Platyhelminthes	0	0	0	0	1.1	0	0	0	0	0
OTU145	Metazoa|Annelida|Spionida|Spionidae|Malacoceros|Malacoceros+fuliginosus	0	0	0	0	0	0	0	0	0	0
OTU146	Metazoa|Arthropoda|Crustacea|Maxillopoda|Copepoda	21.14	0	0	0	0	0	0	0	0	0
OTU147	Metazoa|Porifera	0	0	0	0	0	0	0	0	0	0
OTU149	Metazoa|Mollusca|Bivalvia|Heteroconchia|Macoma|Macoma+nasuta	0	0	0	0	0	0	0	0	0	0
OTU151	Metazoa|Nematoda	0	0	0	0	1.02	0	0	0	0	0
OTU152	Metazoa|Platyhelminthes	0	0	0	0	0	0	0	0	0	0
OTU153	Metazoa|Annelida|Phyllodocida|Phyllodocidae	0	0	0	0	0	0	0	0	0	0
OTU154	Metazoa|Annelida|Spionida|Cirratulidae	0	0	0	0	0	0	0	0	0	0
OTU155	Metazoa|Annelida|Spionida|Cirratulidae	0	0	0	0	0	0	0	0	0	0
OTU156	Metazoa|Arthropoda|Crustacea|Maxillopoda|Copepoda	0	0	0	0	0	0	0	0	0	0
OTU157	Metazoa|Annelida|Phyllodocida|Phyllodocidae	0	0	0	0	3.27	0	0	0	0	0
OTU158	Metazoa|Annelida|Phyllodocida|Phyllodocidae	0	0	0	0	0	0	0	0	0	0
OTU159	Metazoa|Rotifera	0	0	0	0	0	0	0	0	0	0
OTU160	Metazoa|Arthropoda|Crustacea|Maxillopoda|Copepoda	0	0	0	0	0	36.33	0	0	0	0
OTU161	Metazoa|Platyhelminthes	0	0	0	0	0	0	0	0	0	0
OTU162	Metazoa|Annelida|Capitellida|Capitellidae|Capitella	0	0	0	0	0	0	0	0	0	0

OTU163	Metazoa|Nematoda	0	0	0	0	0	0	0	0	0	0
OTU164	Metazoa|Nematoda	0	0	0	0	0	0	0	0	0	0
OTU165	Metazoa|Nematoda	0	0	0	0	0	0	0	0	0	0
OTU167	Metazoa|Nematoda	0	0	0	0	0	0	0	0	0	0
OTU168	Metazoa|Cnidaria|Cnidaria_X|Hydrozoa	0	0	0	0	2.37	0	0	0	0	0
OTU169	Metazoa|Arthropoda|Crustacea|Maxillopoda|Copepoda	0	0	0	0	0	0	6.66	0	0	0
OTU170	Metazoa|Gastrotricha	0	0	0	0	0	0	0	0	0	0
OTU171	Metazoa|Annelida|Phyllodocida|Syllidae	0	0	0	0	0	0	0	0	0	0
OTU173	Metazoa|Nematoda	8.15	0	0	0	0	0	0	0	0	0
OTU174	Metazoa|Arthropoda|Crustacea|Maxillopoda|Copepoda	0	0	0	0	0	0	0	0	0	0
OTU175	Metazoa|Arthropoda|Crustacea|Maxillopoda|Copepoda	0	0	0	0	0	0	0	0	0	0
OTU176	Metazoa|Platyhelminthes	0	0	0	0	0	0	0	0	0	0
OTU178	Metazoa|Nematoda	0	0	0	0	0	0	0	0	0	0
OTU180	Metazoa|Nematoda	0	0	0	0	0	0	0	0	0	0
OTU183	Metazoa|Annelida|Oligochaeta|Tubificidae	0	0	0	0	0	0	0	0	0	0
OTU184	Metazoa|Nematoda	0	0	0	0	0	0	0	0	0	0
OTU186	Metazoa|Platyhelminthes	0	0	0	0	0	0	0	0	0	0
OTU188	Metazoa|Annelida|Terebellida|Ampharetidae	0	0	0	0	0	0	0	0	0	0
OTU190	Metazoa|Annelida|Terebellida|Terebellidae	0	0	0	0	0	0	0	0	0	0
OTU191	Metazoa|Annelida|Phyllodocida|Syllidae	0	0	0	0	0	0	0	0	0	0
OTU192	Metazoa|Annelida|Spionida|Cirratulidae	0	0	0	0	0	0	0	0	0	0

Supplementary Table 7. continued

OTU	assign	DNA_10_1	DNA_10_2	DNA_10_3	DNA_10_4	DNA_10_5	DNA_1_1	DNA_1_2	DNA_1_3	DNA_1_4	DNA_1_5
OTU1	Metazoa|Annelida|Capitellida|Capitellidae|Capitella	0	0	0	0	0	1042.3	2906.41	3826.15	3505.37	3020.62
OTU2	Metazoa|Annelida|Oligochaeta|Tubificidae	0	0	0	0	0	603.95	133.81	0	59.72	40.21
OTU4	Metazoa|Annelida|Phyllodocida|Phyllodocidae|Phyllodoce|Phyllodoce+maculata	0	0	0	0	0	29.22	20.79	7.81	19.04	28.77
OTU5	Metazoa|Annelida|Spionida|Spionidae|Malacoceros|Malacoceros+fuliginosus	0	0	0	0	0	70.64	125.44	129.24	194.7	368.3
OTU6	Metazoa|Annelida|Spionida|Cirratulidae	0	0	0	0	0	0	0	0	0	0
OTU7	Metazoa|Platyhelminthes	0	876.48	0	0	0	899.86	603.38	130.31	242.99	323.43
OTU8	Metazoa|Annelida|Phyllodocida|Syllidae|Salvatoria	0	0	0	0	0	0	0	0	0	0
OTU9	Metazoa|Annelida	2763.11	0	0	294.24	0	0	0	0	0	0
OTU10	Metazoa|Nematoda	0	0	0	0	0	21.34	0	0	0	196.54
OTU11	Metazoa|Nematoda	0	0	0	0	0	133.73	217.12	0	22.26	109.64
OTU12	Metazoa|Nematoda	0	0	0	0	0	12	10.08	0	2.44	0
OTU13	Metazoa|Cnidaria|Cnidaria_X|Anthozoa	0	0	0	0	0	0	0	0	0	0
OTU14	Metazoa|Nemertea	0	0	0	0	0	0	0	0	0	0
OTU15	Metazoa|Mollusca|Bivalvia|Heteroconchia|Abra	0	0	0	0	0	1255.87	0	0	0	0
OTU16	Metazoa|Platyhelminthes	106.33	546.99	1886.34	335.55	230.61	0	0	0	0	0
OTU17	Metazoa|Cnidaria|Cnidaria_X|Hydrozoa	0	979.45	0	2792.76	83.18	0	0	0	0	0
OTU18	Metazoa|Platyhelminthes	0	0	0	0	0	0	74.37	0	0	0
OTU19	Metazoa|Arthropoda|Crustacea|Ostracoda	0	0	0	0	0	0	0	0	0	0
OTU20	Metazoa|Nematoda	0	0	0	0	0	2.2	0	0	0	0
OTU21	Metazoa|Annelida|Terebellida|Ampharetidae	0	0	0	0	0	0	0	0	0	0
OTU22	Metazoa|Annelida|Phyllodocida|Syllidae|Exogone|Exogone+naidina	0	0	0	0	0	0	0	0	0	0
OTU23	Metazoa|Platyhelminthes	0	0	0	0	0	0	0	0	0	0
OTU24	Metazoa|Platyhelminthes	0	0	0	0	0	0	0	0	42.35	0
OTU25	Metazoa|Mollusca|Bivalvia|Heteroconchia|Corbula	0	0	0	0	3513.4	0	0	0	0	0
OTU26	Metazoa|Annelida|Capitellida|Capitellidae|Heteromastus	688.99	0	0	0	0	0	0	0	0	0

OTU27	Metazoa|Arthropoda|Crustacea|Maxillopoda|Copepoda	0	0	0	0	0	0	0	0	0	0
OTU28	Metazoa|Nematoda	0	0	0	0	0	6.57	10.6	3.45	2.68	11.55
OTU29	Metazoa|Cnidaria|Cnidaria_X|Hydrozoa	0	6.55	0	0	0	0	0	0	0	0
OTU30	Metazoa|Mollusca|Gastropoda|Heterobranchia|Bulla	0	0	0	0	0	0	0	0	0	0
OTU31	Metazoa|Platyhelminthes	135.6	0	0	151.6	0	0	0	0	0	0
OTU32	Metazoa|Arthropoda|Crustacea|Ostracoda	0	0	0	0	0	0	0	0	0	0
OTU33	Metazoa|Nemertea	0	0	0	0	0	0	0	0	0	0
OTU35	Metazoa|Nemertea	0	1263.05	0	0	0	0	0	0	0	0
OTU36	Metazoa|Arthropoda|Chelicerata	17.84	0	1721.08	356.68	0	0	0	0	0	0
OTU37	Metazoa|Annelida|Phyllodocida|Syllidae|Erinaceusyllis|Erinaceusyllis+cryptica	0	0	0	0	0	0	0	0	0	0
OTU38	Metazoa|Platyhelminthes	0	0	0	0	0	0	0	0	0	0
OTU39	Metazoa|Arthropoda|Crustacea|Maxillopoda|Copepoda	0	0	143.86	0	0	0	0	0	0	0
OTU40	Metazoa|Annelida|Capitellida|Maldanidae|Praxillella	0	0	0	0	0	0	0	0	0	0
OTU41	Metazoa|Arthropoda|Crustacea|Maxillopoda|Copepoda	0	0	0	0	0	0	0	0	0	0
OTU42	Metazoa|Annelida|Phyllodocida|Syllidae|Exogone|Exogone+naidina	0	0	0	0	0	0	0	0	0	0
OTU43	Metazoa|Hemichordata	0	0	0	0	23.49	0	0	0	0	0
OTU44	Metazoa|Cnidaria|Cnidaria_X|Hydrozoa	137.24	0	204.43	0	0	0	0	0	0	0
OTU45	Metazoa|Mollusca|Bivalvia|Protobranchia|Nucula	0	0	0	0	0	0	0	5.04	0	0
OTU46	Metazoa|Annelida|Capitellida|Capitellidae|Capitella	0	0	0	0	0	0	0	0	0	0
OTU47	Metazoa|Platyhelminthes	0	0	0	0	0	0	0	0	0	0
OTU48	Metazoa|Platyhelminthes	0	0	0	0	0	0	0	0	0	0
OTU49	Metazoa|Platyhelminthes	215.44	0	0	0	0	0	0	0	0	0
OTU51	Metazoa|Nematoda	0	0	0	0	0	0	0	0	0	0
OTU52	Metazoa|Annelida|Capitellida|Maldanidae	0	219.64	0	0	0	0	0	0	0	0
OTU53	Metazoa|Nematoda	0	0	0	0	0	0	0	0	0	0
OTU54	Metazoa|Annelida|Oligochaeta|Tubificidae	0	0	0	0	0	0	0	0	0	0
OTU55	Metazoa|Platyhelminthes	0	0	0	0	0	0	0	0	0	0
OTU56	Metazoa|Platyhelminthes	0	0	0	0	0	0	0	0	0	0
OTU58	Metazoa|Nematoda	0	0	0	0	0	0	0	0	0	0

OTU59	Metazoa|Echinodermata|Echinodermata_X|Echinodermata_XX|Amphiura	0	0	0	0	0	0	0	0	0	0
OTU60	Metazoa|Platyhelminthes	0	0	0	0	0	0	0	0	0	0
OTU61	Metazoa|Cnidaria|Cnidaria_X|Hydrozoa	0	0	0	0	123.38	0	0	0	0	0
OTU62	Metazoa|Platyhelminthes	0	0	0	0	0	21.3	0	0	9.29	0
OTU64	Metazoa|Arthropoda|Crustacea|Maxillopoda|Copepoda	0	0	0	0	0	0	0	0	0	0
OTU65	Metazoa|Nematoda	0	0	0	0	115.91	0	0	0	0	0
OTU66	Metazoa|Arthropoda|Crustacea|Maxillopoda|Copepoda	0	0	0	0	0	0	0	0	0	0
OTU68	Metazoa|Platyhelminthes	0	126.26	0	0	0	0	0	0	0	0
OTU69	Metazoa|Platyhelminthes	0	0	0	0	0	0	0	0	0	0
OTU70	Metazoa|Arthropoda|Crustacea|Maxillopoda|Copepoda	0	0	0	0	0	0	0	0	0	0
OTU71	Metazoa|Platyhelminthes	0	0	0	0	0	0	0	0	0	0
OTU72	Metazoa|Nematoda	0	0	0	0	0	0	0	0	0	0
OTU73	Metazoa|Nematoda	0	0	0	0	0	0	0	0	0	0
OTU75	Metazoa|Gastrotricha	19.28	4.15	62.25	30.31	12.03	0	0	0	0	0
OTU76	Metazoa|Arthropoda|Crustacea|Maxillopoda|Copepoda	0	0	0	0	0	0	0	0	0	0
OTU78	Metazoa|Mollusca|Gastropoda|Heterobranchia|Calliopaea|Calliopaea+bellula	0	0	0	0	0	0	0	0	0	0
OTU79	Metazoa|Annelida|Phyllodocida|Syllidae	0	0	0	0	0	0	0	0	0	0
OTU80	Metazoa|Echinodermata|Echinodermata_X|Echinodermata_XX|Leptosynapta	0	0	0	0	0	0	0	0	0	0
OTU82	Metazoa|Annelida|Capitellida|Capitellidae|Capitella	0	0	0	0	0	0	0	0	0	0
OTU84	Metazoa|Annelida|Capitellida|Capitellidae|Capitella	0	0	0	0	0	0	0	0	0	0
OTU85	Metazoa|Platyhelminthes	0	0	0	0	0	0	0	0	0	0
OTU86	Metazoa|Platyhelminthes	0	0	0	0	0	0	0	0	0	0
OTU87	Metazoa|Annelida|Terebellida|Terebellidae	0	0	0	0	0	0	0	0	0	0
OTU88	Metazoa|Platyhelminthes	0	0	0	0	0	0	0	0	0	0
OTU89	Metazoa|Annelida|Oligochaeta|Tubificidae	0	0	0	0	0	0	0	0	0	0
OTU90	Metazoa|Cnidaria|Cnidaria_X|Anthozoa|Ceriantheopsis	0	0	0	0	0	0	0	0	0	0
OTU91	Metazoa|Annelida|Capitellida|Scalibregmatidae|Scalibregma|Scalibregma+inflatum	0	68.74	0	0	0	0	0	0	0	0
OTU92	Metazoa|Annelida|Scolecida|Opheliidae|Ophelina	0	0	0	0	0	0	0	0	0	0
OTU93	Metazoa|Gastrotricha	0	10.69	84.04	0	0	0	0	0	0	0

OTU94	Metazoa|Platyhelminthes	0	0	0	0	0	0	0	0	0	0
OTU95	Metazoa|Arthropoda|Crustacea|Ostracoda	0	0	0	0	0	0	0	0	0	0
OTU96	Metazoa|Platyhelminthes	0	0	0	0	0	0	0	0	0	0
OTU97	Metazoa|Platyhelminthes	0	0	0	0	0	0	0	0	0	0
OTU98	Metazoa|Platyhelminthes	0	0	0	0	0	0	0	0	0	0
OTU99	Metazoa|Platyhelminthes	0	0	0	0	0	0	0	0	0	0
OTU100	Metazoa|Arthropoda|Crustacea|Ostracoda	0	0	0	0	0	0	0	0	0	0
OTU101	Metazoa|Annelida|Phyllodocida|Phyllodocidae	0	0	0	0	0	0	0	0	0	0
OTU102	Metazoa|Arthropoda|Crustacea|Maxillopoda|Copepoda	0	0	0	0	0	0	0	0	0	0
OTU103	Metazoa|Nemertea	0	0	0	0	0	0	0	0	0	0
OTU104	Metazoa|Annelida|Phyllodocida|Syllidae	0	0	0	0	0	0	0	0	0	0
OTU107	Metazoa|Arthropoda|Crustacea|Ostracoda	0	0	0	0	0	0	0	0	0	0
OTU108	Metazoa|Annelida|Spionida|Spionidae|Malacoceros|Malacoceros+fuliginosus	0	0	0	0	0	0	0	0	0	0
OTU109	Metazoa|Arthropoda|Crustacea|Maxillopoda|Copepoda	0	0	0	0	0	0	0	0	0	0
OTU110	Metazoa|Arthropoda|Crustacea|Maxillopoda|Copepoda	0	0	0	0	0	0	0	0	0	0
OTU111	Metazoa|Kinorhyncha	0	0	0	0	0	0	0	0	0	0
OTU112	Metazoa|Platyhelminthes	0	0	0	0	0	0	0	0	0	0
OTU113	Metazoa|Nematoda	0	0	0	0	0	0	0	0	0	0
OTU114	Metazoa|Nematoda	0	0	0	0	0	0	0	0	0	0
OTU115	Metazoa	0	0	0	50.88	0	0	0	0	0	0
OTU116	Metazoa|Platyhelminthes	0	0	0	0	0	0	0	0	0	0
OTU117	Metazoa|Nematoda	0	0	0	0	0	0	0	0	0	0
OTU118	Metazoa|Arthropoda|Crustacea|Maxillopoda|Copepoda	0	0	0	0	0	0	0	0	0	0
OTU119	Metazoa|Nematoda	0	0	0	0	0	0	0	0	0	0
OTU120	Metazoa|Platyhelminthes	0	0	0	0	0	0	0	0	0	0
OTU121	Metazoa|Nematoda	0	0	0	0	0	0	0	0	0	0
OTU122	Metazoa|Annelida|Oligochaeta|Tubificidae	0	0	0	0	0	0	0	0	0	0
OTU123	Metazoa|Mollusca|Bivalvia|Heteroconchia|Myrtea|Myrtea+spinifera	0	0	0	0	0	0	0	0	0	0
OTU124	Metazoa|Annelida|Terebellida|Pectinariidae|Pectinaria	0	0	0	0	0	0	0	0	0	0

OTU125	Metazoa|Platyhelminthes	0	0	0	0	0	0	0	0	0	0
OTU126	Metazoa|Nematoda	0	0	0	50.09	0	0	0	0	0	0
OTU128	Metazoa|Kinorhyncha	0	0	0	0	0	0	0	0	0	0
OTU130	Metazoa|Annelida|Phyllodocida|Phyllodocidae	0	0	0	0	0	0	0	0	0	0
OTU131	Metazoa|Annelida|Oligochaeta|Tubificidae	0	0	0	0	0	0	0	0	0	0
OTU132	Metazoa|Nematoda	18.17	0	0	0	0	0	0	0	0	0
OTU134	Metazoa|Annelida|Phyllodocida|Syllidae	0	0	0	0	0	0	0	0	0	0
OTU135	Metazoa|Annelida|Capitellida|Capitellidae|Capitella	0	0	0	0	0	0	0	0	0	0
OTU136	Metazoa|Nematoda	0	0	0	0	0	0	0	0	0	0
OTU137	Metazoa|Hemichordata	0	0	0	0	0	0	0	0	0	0
OTU138	Metazoa	0	0	0	0	0	0	0	0	0	0
OTU141	Metazoa|Nematoda	0	0	0	0	0	0	0	0	0	0
OTU143	Metazoa|Annelida|Capitellida|Capitellidae|Capitella	0	0	0	0	0	0	0	0	0	0
OTU144	Metazoa|Platyhelminthes	0	0	0	0	0	0	0	0	0	0
OTU145	Metazoa|Annelida|Spionida|Spionidae|Malacoceros|Malacoceros+fuliginosus	0	0	0	0	0	0	0	0	0	2.94
OTU146	Metazoa|Arthropoda|Crustacea|Maxillopoda|Copepoda	0	0	0	0	0	0	0	0	0	0
OTU147	Metazoa|Porifera	0	0	0	0	0	0	0	0	0	0
OTU149	Metazoa|Mollusca|Bivalvia|Heteroconchia|Macoma|Macoma+nasuta	0	0	0	0	0	3.02	0	0	0	0
OTU151	Metazoa|Nematoda	0	0	0	0	0	0	0	0	0	0
OTU152	Metazoa|Platyhelminthes	0	0	0	0	0	0	0	0	0	0
OTU153	Metazoa|Annelida|Phyllodocida|Phyllodocidae	0	0	0	0	0	0	0	0	0	0
OTU154	Metazoa|Annelida|Spionida|Cirratulidae	0	0	0	0	0	0	0	0	0	0
OTU155	Metazoa|Annelida|Spionida|Cirratulidae	0	0	0	0	0	0	0	0	0	0
OTU156	Metazoa|Arthropoda|Crustacea|Maxillopoda|Copepoda	0	0	0	0	0	0	0	0	0	0
OTU157	Metazoa|Annelida|Phyllodocida|Phyllodocidae	0	0	0	0	0	0	0	0	0	0
OTU158	Metazoa|Annelida|Phyllodocida|Phyllodocidae	0	0	0	0	0	0	0	0	0	0
OTU159	Metazoa|Rotifera	0	0	0	0	0	0	0	0	0	0
OTU160	Metazoa|Arthropoda|Crustacea|Maxillopoda|Copepoda	0	0	0	0	0	0	0	0	0	0
OTU161	Metazoa|Platyhelminthes	0	0	0	0	0	0	0	0	0	0

OTU162	Metazoa|Annelida|Capitellida|Capitellidae|Capitella	0	0	0	0	0	0	0	0	0	0
OTU163	Metazoa|Nematoda	0	0	0	0	0	0	0	0	0	0
OTU164	Metazoa|Nematoda	0	0	0	0	0	0	0	0	0	0
OTU165	Metazoa|Nematoda	0	0	0	0	0	0	0	0	0	0
OTU167	Metazoa|Nematoda	0	0	0	0	0	0	0	0	0	0
OTU168	Metazoa|Cnidaria|Cnidaria_X|Hydrozoa	0	0	0	0	0	0	0	0	0	0
OTU169	Metazoa|Arthropoda|Crustacea|Maxillopoda|Copepoda	0	0	0	0	0	0	0	0	0	0
OTU170	Metazoa|Gastrotricha	0	0	0	0	0	0	0	0	0	0
OTU171	Metazoa|Annelida|Phyllodocida|Syllidae	0	0	0	0	0	0	0	0	0	0
OTU173	Metazoa|Nematoda	0	0	0	0	0	0	0	0	0	0
OTU174	Metazoa|Arthropoda|Crustacea|Maxillopoda|Copepoda	0	0	0	0	0	0	0	0	0	0
OTU175	Metazoa|Arthropoda|Crustacea|Maxillopoda|Copepoda	0	0	0	39.89	0	0	0	0	0	0
OTU176	Metazoa|Platyhelminthes	0	0	0	0	0	0	0	0	0	0
OTU178	Metazoa|Nematoda	0	0	0	0	0	0	0	0	0	0
OTU180	Metazoa|Nematoda	0	0	0	0	0	0	0	0	0	0
OTU183	Metazoa|Annelida|Oligochaeta|Tubificidae	0	0	0	0	0	0	0	0	0	0
OTU184	Metazoa|Nematoda	0	0	0	0	0	0	0	0	0	0
OTU186	Metazoa|Platyhelminthes	0	0	0	0	0	0	0	0	0	0
OTU188	Metazoa|Annelida|Terebellida|Ampharetidae	0	0	0	0	0	0	0	0	0	0
OTU190	Metazoa|Annelida|Terebellida|Terebellidae	0	0	0	0	0	0	0	0	0	0
OTU191	Metazoa|Annelida|Phyllodocida|Syllidae	0	0	0	0	0	0	0	0	0	0
OTU192	Metazoa|Annelida|Spionida|Cirratulidae	0	0	0	0	0	0	0	0	0	0

Supplementary Table 7. continued

OTU	assign	DNA_2_1	DNA_2_2	DNA_2_3	DNA_2_4	DNA_2_5	DNA_3_1	DNA_3_2	DNA_3_3	DNA_3_4	DNA_3_5
OTU1	Metazoa|Annelida|Capitellida|Capitellidae|Capitella	495.55	2481.66	3402.09	13.12	225.38	520.1	1145.55	3276.99	297.58	1451.73
OTU2	Metazoa|Annelida|Oligochaeta|Tubificidae	428.87	1.09	47.74	18.3	1484.44	2669.49	1945.84	197.89	2296.95	2205.65
OTU4	Metazoa|Annelida|Phyllodocida|Phyllodocidae|Phyllodoce|Phyllodoce+maculata	752.49	138.57	54.94	12.97	0	264.1	171.32	213.1	242.6	135.97
OTU5	Metazoa|Annelida|Spionida|Spionidae|Malacoceros|Malacoceros+fuliginosus	1938.62	458.31	0	4002.04	1003.96	0	0	11.7	2.65	6.25
OTU6	Metazoa|Annelida|Spionida|Cirratulidae	0	0	0	0	0	0	0	348.69	752.02	33.03
OTU7	Metazoa|Platyhelminthes	269.62	290.07	264.22	17.31	547.29	0	244.18	9.72	98.95	177.83
OTU8	Metazoa|Annelida|Phyllodocida|Syllidae|Salvatoria	0	0	0	0	73.5	0	0	0	169.28	0
OTU9	Metazoa|Annelida	0	38.25	220.21	35.56	0	0	0	0	0	13.97
OTU10	Metazoa|Nematoda	18.69	200.46	0	0	238.38	0	0	0	0	0
OTU11	Metazoa|Nematoda	0	3.06	0	0	10.33	16.24	63.42	26.64	9.77	9.32
OTU12	Metazoa|Nematoda	5.49	0	1.46	1.46	10.26	209.86	226.51	1.83	53.69	4
OTU13	Metazoa|Cnidaria|Cnidaria_X|Anthozoa	0	0	101.47	0	0	111.85	0	2.5	0	0
OTU14	Metazoa|Nemertea	0	0	0	0	0	0	0	0	0	0
OTU15	Metazoa|Mollusca|Bivalvia|Heteroconchia|Abra	0	0	0	0	0	0	0	0	0	42.85
OTU16	Metazoa|Platyhelminthes	0	0	0	0	0	0	0	0	0	0
OTU17	Metazoa|Cnidaria|Cnidaria_X|Hydrozoa	0	0	0	0	0	0	0	0	0	0
OTU18	Metazoa|Platyhelminthes	0	64.2	0	0	0	0	11.43	0	0	3.44
OTU19	Metazoa|Arthropoda|Crustacea|Ostracoda	0	0	0	0	0	0	0	0	0	0
OTU20	Metazoa|Nematoda	2.01	0	0	0	2.59	55.56	86.34	2.4	70.03	2.53
OTU21	Metazoa|Annelida|Terebellida|Ampharetidae	0	0	0	0	0	0	0	0	0	0
OTU22	Metazoa|Annelida|Phyllodocida|Syllidae|Exogone|Exogone+naidina	0	409.99	0	0	0	0	0	0	0	0
OTU23	Metazoa|Platyhelminthes	0	0	0	0	0	0	0	0	0	0
OTU24	Metazoa|Platyhelminthes	158.87	0	0	0	469.99	0	0	0	0	0
OTU25	Metazoa|Mollusca|Bivalvia|Heteroconchia|Corbula	0	1.57	0	0	0	0	0	0	0	0
OTU26	Metazoa|Annelida|Capitellida|Capitellidae|Heteromastus	0	0	0	0	0	0	17.37	0	0	0

OTU27	Metazoa|Arthropoda|Crustacea|Maxillopoda|Copepoda	0	0	0	0	0	0	0	0	0	0
OTU28	Metazoa|Nematoda	6.39	9.29	3.12	1.24	29.15	239.39	190.04	5.34	92.68	7.73
OTU29	Metazoa|Cnidaria|Cnidaria_X|Hydrozoa	0	0	0	0	0	0	0	0	0	0
OTU30	Metazoa|Mollusca|Gastropoda|Heterobranchia|Bulla	0	0	0	0	0	0	0	0	0	0
OTU31	Metazoa|Platyhelminthes	0	0	0	0	0	0	0	0	0	0
OTU32	Metazoa|Arthropoda|Crustacea|Ostracoda	0	0	0	0	0	0	0	0	0	0
OTU33	Metazoa|Nemertea	0	0	0	0	0	0	0	0	0	0
OTU35	Metazoa|Nemertea	0	0	0	0	0	0	0	0	0	0
OTU36	Metazoa|Arthropoda|Chelicerata	0	0	0	0	0	0	0	0	0	1.18
OTU37	Metazoa|Annelida|Phyllodocida|Syllidae|Erinaceusyllis|Erinaceusyllis+cryptica	0	0	0	0	0	0	0	0	0	0
OTU38	Metazoa|Platyhelminthes	0	0	0	0	0	0	0	0	0	0
OTU39	Metazoa|Arthropoda|Crustacea|Maxillopoda|Copepoda	0	0	0	0	0	0	0	0	0	0
OTU40	Metazoa|Annelida|Capitellida|Maldanidae|Praxillella	0	0	0	0	0	0	0	0	0	0
OTU41	Metazoa|Arthropoda|Crustacea|Maxillopoda|Copepoda	0	0	0	0	0	0	0	0	0	0
OTU42	Metazoa|Annelida|Phyllodocida|Syllidae|Exogone|Exogone+naidina	0	0	0	0	0	0	0	3.64	0	0
OTU43	Metazoa|Hemichordata	0	0	0	0	0	0	0	0	0	0
OTU44	Metazoa|Cnidaria|Cnidaria_X|Hydrozoa	0	0	0	0	1.18	0	0	0	0	0
OTU45	Metazoa|Mollusca|Bivalvia|Protobranchia|Nucula	0	0	0	0	0	0	0	0	0	0
OTU46	Metazoa|Annelida|Capitellida|Capitellidae|Capitella	0	0	0	0	0	0	0	0	0	0
OTU47	Metazoa|Platyhelminthes	0	0	0	0	0	0	0	0	0	0
OTU48	Metazoa|Platyhelminthes	0	0	0	0	0	0	0	0	0	0
OTU49	Metazoa|Platyhelminthes	0	0	0	0	0	0	0	0	0	0
OTU51	Metazoa|Nematoda	0	0	0	0	0	0	0	0	0	0
OTU52	Metazoa|Annelida|Capitellida|Maldanidae	0	0	0	0	0	0	0	0	0	0
OTU53	Metazoa|Nematoda	0	3.27	0	0	3.57	0	0	0	0	0
OTU54	Metazoa|Annelida|Oligochaeta|Tubificidae	0	0	0	0	0	0	0	0	0	3.15
OTU55	Metazoa|Platyhelminthes	0	0	0	0	0	0	0	0	0	0
OTU56	Metazoa|Platyhelminthes	0	0	0	0	0	0	0	0	0	0
OTU58	Metazoa|Nematoda	0	0	0	0	0	0	0	0	0	0

OTU59	Metazoa|Echinodermata|Echinodermata_X|Echinodermata_XX|Amphiura	0	0	0	0	0	0	0	0	0	0
OTU60	Metazoa|Platyhelminthes	0	0	0	0	0	0	0	0	0	0
OTU61	Metazoa|Cnidaria|Cnidaria_X|Hydrozoa	0	0	0	0	0	0	0	0	0	0
OTU62	Metazoa|Platyhelminthes	10.78	1.6	6.75	0	0	0	0	0	0	0
OTU64	Metazoa|Arthropoda|Crustacea|Maxillopoda|Copepoda	0	0	0	0	0	0	0	0	0	0
OTU65	Metazoa|Nematoda	0	0	0	0	0	0	0	0	0	0
OTU66	Metazoa|Arthropoda|Crustacea|Maxillopoda|Copepoda	0	0	0	0	0	0	0	0	0	0
OTU68	Metazoa|Platyhelminthes	0	0	0	0	0	0	0	0	0	0
OTU69	Metazoa|Platyhelminthes	0	0	0	0	0	0	0	0	0	0
OTU70	Metazoa|Arthropoda|Crustacea|Maxillopoda|Copepoda	0	0	0	0	0	0	0	0	0	0
OTU71	Metazoa|Platyhelminthes	0	0	0	0	0	0	0	0	0	0
OTU72	Metazoa|Nematoda	0	0	0	0	0	12.19	0	0	7.42	0
OTU73	Metazoa|Nematoda	0	0	0	0	0	0	0	0	0	0
OTU75	Metazoa|Gastrotricha	0	0	0	0	0	0	0	0	0	0
OTU76	Metazoa|Arthropoda|Crustacea|Maxillopoda|Copepoda	0	0	0	0	0	0	0	0	0	0
OTU78	Metazoa|Mollusca|Gastropoda|Heterobranchia|Calliopaea|Calliopaea+bellula	0	0	0	0	0	0	0	0	0	0
OTU79	Metazoa|Annelida|Phyllodocida|Syllidae	0	0	0	0	0	0	0	0	0	0
OTU80	Metazoa|Echinodermata|Echinodermata_X|Echinodermata_XX|Leptosynapta	0	0	0	0	0	0	0	0	0	0
OTU82	Metazoa|Annelida|Capitellida|Capitellidae|Capitella	0	0	0	0	0	0	0	0	0	0
OTU84	Metazoa|Annelida|Capitellida|Capitellidae|Capitella	0	0	0	0	0	0	0	0	0	0
OTU85	Metazoa|Platyhelminthes	0	0	0	0	0	0	0	0	0	0
OTU86	Metazoa|Platyhelminthes	0	0	0	0	0	0	0	0	0	0
OTU87	Metazoa|Annelida|Terebellida|Terebellidae	0	0	0	0	0	0	0	0	0	0
OTU88	Metazoa|Platyhelminthes	0	0	0	0	1.98	0	0	0	0	0
OTU89	Metazoa|Annelida|Oligochaeta|Tubificidae	0	0	0	0	0	3.22	0	0	4.56	0
OTU90	Metazoa|Cnidaria|Cnidaria_X|Anthozoa|Ceriantheopsis	0	0	0	0	0	0	0	0	0	0
OTU91	Metazoa|Annelida|Capitellida|Scalibregmatidae|Scalibregma|Scalibregma+inflatum	0	0	0	0	0	0	0	0	0	0
OTU92	Metazoa|Annelida|Scolecida|Opheliidae|Ophelina	0	0	0	0	0	0	0	0	0	0
OTU93	Metazoa|Gastrotricha	0	0	0	0	0	0	0	0	0	0

OTU94	Metazoa|Platyhelminthes	0	0	0	0	0	0	0	0	0	0
OTU95	Metazoa|Arthropoda|Crustacea|Ostracoda	0	0	0	0	0	0	0	0	0	0
OTU96	Metazoa|Platyhelminthes	0	0	0	0	0	0	0	0	0	0
OTU97	Metazoa|Platyhelminthes	0	0	0	0	0	0	0	0	0	0
OTU98	Metazoa|Platyhelminthes	0	0	0	0	0	0	0	0	0	0
OTU99	Metazoa|Platyhelminthes	0	0	0	0	0	0	0	0	0	0
OTU100	Metazoa|Arthropoda|Crustacea|Ostracoda	0	0	0	0	0	0	0	0	0	0
OTU101	Metazoa|Annelida|Phyllodocida|Phyllodocidae	0	0	0	0	0	0	0	0	0	0
OTU102	Metazoa|Arthropoda|Crustacea|Maxillopoda|Copepoda	0	0	0	0	0	0	0	0	0	0
OTU103	Metazoa|Nemertea	0	0	0	0	0	0	0	0	0	0
OTU104	Metazoa|Annelida|Phyllodocida|Syllidae	0	0	0	0	0	0	0	0	0	0
OTU107	Metazoa|Arthropoda|Crustacea|Ostracoda	0	0	0	0	0	0	0	0	0	0
OTU108	Metazoa|Annelida|Spionida|Spionidae|Malacoceros|Malacoceros+fuliginosus	6.87	0	0	0	0	0	0	0	0	0
OTU109	Metazoa|Arthropoda|Crustacea|Maxillopoda|Copepoda	0	0	0	0	0	0	0	0	0	0
OTU110	Metazoa|Arthropoda|Crustacea|Maxillopoda|Copepoda	0	0	0	0	0	0	0	0	0	0
OTU111	Metazoa|Kinorhyncha	0	0	0	0	0	0	0	0	0	0
OTU112	Metazoa|Platyhelminthes	0	0	0	0	0	0	0	0	0	0
OTU113	Metazoa|Nematoda	0	0	0	0	0	0	0	0	0	0
OTU114	Metazoa|Nematoda	0	0	0	0	0	0	0	0	0	0
OTU115	Metazoa	0	0	0	0	0	0	0	0	0	0
OTU116	Metazoa|Platyhelminthes	0	0	0	0	0	0	0	0	0	0
OTU117	Metazoa|Nematoda	0	0	0	0	0	0	0	0	0	0
OTU118	Metazoa|Arthropoda|Crustacea|Maxillopoda|Copepoda	0	0	0	0	0	0	0	0	0	0
OTU119	Metazoa|Nematoda	0	0	0	0	0	0	0	0	0	0
OTU120	Metazoa|Platyhelminthes	0	0	0	0	0	0	0	0	0	0
OTU121	Metazoa|Nematoda	0	0	0	0	0	0	0	0	0	0
OTU122	Metazoa|Annelida|Oligochaeta|Tubificidae	0	0	0	0	0	0	0	0	0	0
OTU123	Metazoa|Mollusca|Bivalvia|Heteroconchia|Myrtea|Myrtea+spinifera	0	0	0	0	0	0	0	0	0	0
OTU124	Metazoa|Annelida|Terebellida|Pectinariidae|Pectinaria	0	0	0	0	0	0	0	0	0	0

OTU125	Metazoa|Platyhelminthes	0	0	0	0	0	0	0	0	0	0
OTU126	Metazoa|Nematoda	0	0	0	0	0	0	0	0	0	0
OTU128	Metazoa|Kinorhyncha	0	0	0	0	0	0	0	0	0	0
OTU130	Metazoa|Annelida|Phyllodocida|Phyllodocidae	0	0	0	0	0	0	0	0	0	0
OTU131	Metazoa|Annelida|Oligochaeta|Tubificidae	0	0	0	0	0	0	0	0	0	0
OTU132	Metazoa|Nematoda	0	0	0	0	0	0	0	0	0	0
OTU134	Metazoa|Annelida|Phyllodocida|Syllidae	0	0	0	0	0	0	0	0	0	0
OTU135	Metazoa|Annelida|Capitellida|Capitellidae|Capitella	0	0	0	0	0	0	0	0	0	0
OTU136	Metazoa|Nematoda	0	0	0	0	0	0	0	0	0	0
OTU137	Metazoa|Hemichordata	0	0	0	0	0	0	0	0	0	0
OTU138	Metazoa	0	0	0	0	0	0	0	0	0	0
OTU141	Metazoa|Nematoda	0	0	0	0	0	0	0	0	0	0
OTU143	Metazoa|Annelida|Capitellida|Capitellidae|Capitella	3.61	0	0	0	0	0	0	0	0	0
OTU144	Metazoa|Platyhelminthes	0	0	0	0	0	0	0	0	0	0
OTU145	Metazoa|Annelida|Spionida|Spionidae|Malacoceros|Malacoceros+fuliginosus	1.01	0	0	0	0	0	0	0	0	0
OTU146	Metazoa|Arthropoda|Crustacea|Maxillopoda|Copepoda	0	0	0	0	0	0	0	0	0	0
OTU147	Metazoa|Porifera	0	0	0	0	0	0	0	0	0	0
OTU149	Metazoa|Mollusca|Bivalvia|Heteroconchia|Macoma|Macoma+nasuta	0	0	0	0	0	0	0	0	0	3.37
OTU151	Metazoa|Nematoda	0	0	0	0	0	0	0	0	0	0
OTU152	Metazoa|Platyhelminthes	0	0	0	0	0	0	0	0	0	0
OTU153	Metazoa|Annelida|Phyllodocida|Phyllodocidae	3.13	0	0	0	0	0	0	0	0	0
OTU154	Metazoa|Annelida|Spionida|Cirratulidae	0	0	0	0	0	0	0	0	0	0
OTU155	Metazoa|Annelida|Spionida|Cirratulidae	0	0	0	0	0	0	0	0	0	0
OTU156	Metazoa|Arthropoda|Crustacea|Maxillopoda|Copepoda	0	0	0	0	0	0	0	0	0	0
OTU157	Metazoa|Annelida|Phyllodocida|Phyllodocidae	0	0	0	0	0	0	0	0	0	0
OTU158	Metazoa|Annelida|Phyllodocida|Phyllodocidae	0	0	0	0	0	0	0	0	0	0
OTU159	Metazoa|Rotifera	0	0	0	0	0	0	0	0	3.82	0
OTU160	Metazoa|Arthropoda|Crustacea|Maxillopoda|Copepoda	0	0	0	0	0	0	0	0	0	0
OTU161	Metazoa|Platyhelminthes	0	0	0	0	0	0	0	0	0	0

OTU162	Metazoa|Annelida|Capitellida|Capitellidae|Capitella	0	0	0	0	0	0	0	0	0	0
OTU163	Metazoa|Nematoda	0	0	0	0	0	0	0	0	0	0
OTU164	Metazoa|Nematoda	0	0	0	0	0	0	0	0	0	0
OTU165	Metazoa|Nematoda	0	0	0	0	0	0	0	0	0	0
OTU167	Metazoa|Nematoda	0	0	0	0	0	0	0	0	0	0
OTU168	Metazoa|Cnidaria|Cnidaria_X|Hydrozoa	0	0	0	0	0	0	0	0	0	0
OTU169	Metazoa|Arthropoda|Crustacea|Maxillopoda|Copepoda	0	0	0	0	0	0	0	0	0	0
OTU170	Metazoa|Gastrotricha	0	0	0	0	0	0	0	0	0	0
OTU171	Metazoa|Annelida|Phyllodocida|Syllidae	0	0	0	0	0	0	0	0	0	0
OTU173	Metazoa|Nematoda	0	0	0	0	0	0	0	0	0	0
OTU174	Metazoa|Arthropoda|Crustacea|Maxillopoda|Copepoda	0	0	0	0	0	0	0	0	0	0
OTU175	Metazoa|Arthropoda|Crustacea|Maxillopoda|Copepoda	0	0	0	0	0	0	0	0	0	0
OTU176	Metazoa|Platyhelminthes	0	0	0	0	0	0	0	0	0	0
OTU178	Metazoa|Nematoda	0	0	0	0	0	0	0	0	0	0
OTU180	Metazoa|Nematoda	0	0	0	0	0	0	0	0	0	0
OTU183	Metazoa|Annelida|Oligochaeta|Tubificidae	0	0	0	0	0	0	0	0	0	0
OTU184	Metazoa|Nematoda	0	0	0	0	0	0	0	0	0	0
OTU186	Metazoa|Platyhelminthes	0	0	0	0	0	0	0	0	0	0
OTU188	Metazoa|Annelida|Terebellida|Ampharetidae	0	0	0	0	0	0	0	0	0	0
OTU190	Metazoa|Annelida|Terebellida|Terebellidae	0	0	0	0	0	0	0	0	0	0
OTU191	Metazoa|Annelida|Phyllodocida|Syllidae	0	0	0	0	0	0	0	0	0	0
OTU192	Metazoa|Annelida|Spionida|Cirratulidae	0	0	0	0	0	0	0	0	0	0

Supplementary Table 7. continued

OTU	assign	DNA_4_1	DNA_4_2	DNA_4_3	DNA_4_4	DNA_4_5	DNA_5_1	DNA_5_2	DNA_5_3	DNA_5_4	DNA_5_5
OTU1	Metazoa|Annelida|Capitellida|Capitellidae|Capitella	3076.96	8.96	158.65	0	0	849.16	19.83	3504.61	0	0
OTU2	Metazoa|Annelida|Oligochaeta|Tubificidae	458.41	2179.98	3458.96	3002.85	1451.4	0	780.64	119.93	3600.58	1816.97
OTU4	Metazoa|Annelida|Phyllodocida|Phyllodocidae|Phyllodoce|Phyllodoce+maculata	42.77	65.91	49.06	44.86	0	46.52	0	82.68	110.22	1633.71
OTU5	Metazoa|Annelida|Spionida|Spionidae|Malacoceros|Malacoceros+fuliginosus	0	0	0	0	0	0	0	0	0	0
OTU6	Metazoa|Annelida|Spionida|Cirratulidae	383.25	1628.9	365.28	774.17	779.47	2002.82	908.39	162.15	79.67	0
OTU7	Metazoa|Platyhelminthes	22.58	50.26	0	39.77	0	0	52.63	0	40.6	98.78
OTU8	Metazoa|Annelida|Phyllodocida|Syllidae|Salvatoria	0	0	0	0	0	0	1038.32	0	0	0
OTU9	Metazoa|Annelida	0	0	0	0	0	0	223.55	0	0	0
OTU10	Metazoa|Nematoda	0	0	0	0	1694.89	0	0	0	0	106.5
OTU11	Metazoa|Nematoda	6.37	73.13	0	0	0	939.89	915.86	134.33	134.87	357.67
OTU12	Metazoa|Nematoda	58.95	73.32	32.98	168.06	77.9	47.26	34.05	10.36	7.66	33.63
OTU13	Metazoa|Cnidaria|Cnidaria_X|Anthozoa	0	0	0	0	0	0	0	0	0	0
OTU14	Metazoa|Nemertea	0	0	0	0	0	0	0	0	0	0
OTU15	Metazoa|Mollusca|Bivalvia|Heteroconchia|Abra	0	0	0	0	0	0	0	0	0	0
OTU16	Metazoa|Platyhelminthes	0	0	0	0	0	0	0	0	0	0
OTU17	Metazoa|Cnidaria|Cnidaria_X|Hydrozoa	1.6	0	0	0	0	10.83	0	0	0	8.93
OTU18	Metazoa|Platyhelminthes	0	0	0	0	1.36	12.46	3.17	0	0	0
OTU19	Metazoa|Arthropoda|Crustacea|Ostracoda	0	0	7.31	0	0	0	10.32	0	0	0
OTU20	Metazoa|Nematoda	13.04	8.14	29.76	22.08	6.52	107.53	73.4	75.9	34.23	42.58
OTU21	Metazoa|Annelida|Terebellida|Ampharetidae	0	0	0	0	0	0	0	0	0	0
OTU22	Metazoa|Annelida|Phyllodocida|Syllidae|Exogone|Exogone+naidina	0	0	0	0	0	0	0	0	0	0
OTU23	Metazoa|Platyhelminthes	0	0	0	0	0	0	0	0	0	0
OTU24	Metazoa|Platyhelminthes	0	0	0	0	0	0	0	0	94.17	0
OTU25	Metazoa|Mollusca|Bivalvia|Heteroconchia|Corbula	0	0	0	0	0	0	0	0	0	0
OTU26	Metazoa|Annelida|Capitellida|Capitellidae|Heteromastus	2.74	0	0	0	2.42	11.02	0	0	0	0

OTU27	Metazoa|Arthropoda|Crustacea|Maxillopoda|Copepoda	0	0	0	0	0	0	0	0	0	0
OTU28	Metazoa|Nematoda	2.24	6.24	0	15.25	1.58	71.28	19.89	3.24	0	0
OTU29	Metazoa|Cnidaria|Cnidaria_X|Hydrozoa	0	0	0	0	0	0	0	0	0	0
OTU30	Metazoa|Mollusca|Gastropoda|Heterobranchia|Bulla	0	0	0	0	65.1	0	2.67	0	0	0
OTU31	Metazoa|Platyhelminthes	0	0	0	0	0	0	0	0	0	0
OTU32	Metazoa|Arthropoda|Crustacea|Ostracoda	0	0	0	0	0	0	0	0	0	0
OTU33	Metazoa|Nemertea	0	0	0	0	0	0	0	0	0	0
OTU35	Metazoa|Nemertea	0	0	0	0	0	0	0	0	0	0
OTU36	Metazoa|Arthropoda|Chelicerata	4.13	0	0	0	0	0	0	1.79	0	0
OTU37	Metazoa|Annelida|Phyllodocida|Syllidae|Erinaceusyllis|Erinaceusyllis+cryptica	0	0	0	0	0	0	0	0	0	0
OTU38	Metazoa|Platyhelminthes	0	0	0	0	0	0	0	0	0	0
OTU39	Metazoa|Arthropoda|Crustacea|Maxillopoda|Copepoda	0	0	0	0	0	0	0	0	0	0
OTU40	Metazoa|Annelida|Capitellida|Maldanidae|Praxillella	0	0	0	0	0	0	0	0	0	0
OTU41	Metazoa|Arthropoda|Crustacea|Maxillopoda|Copepoda	0	0	0	0	0	0	0	0	0	0
OTU42	Metazoa|Annelida|Phyllodocida|Syllidae|Exogone|Exogone+naidina	0	0	0	0	0	0	0	0	0	0
OTU43	Metazoa|Hemichordata	0	0	0	0	0	0	0	0	0	0
OTU44	Metazoa|Cnidaria|Cnidaria_X|Hydrozoa	0	0	0	0	0	0	0	0	0	0
OTU45	Metazoa|Mollusca|Bivalvia|Protobranchia|Nucula	0	0	0	0	0	0	0	0	0	0
OTU46	Metazoa|Annelida|Capitellida|Capitellidae|Capitella	0	0	0	0	0	0	0	0	0	0
OTU47	Metazoa|Platyhelminthes	0	0	0	0	0	0	0	0	0	0
OTU48	Metazoa|Platyhelminthes	0	0	0	0	0	0	0	0	0	0
OTU49	Metazoa|Platyhelminthes	0	0	0	0	0	0	0	0	0	0
OTU51	Metazoa|Nematoda	0	0	0	0	0	0	0	0	0	3.23
OTU52	Metazoa|Annelida|Capitellida|Maldanidae	0	0	0	0	0	0	0	0	0	0
OTU53	Metazoa|Nematoda	0	0	0	0	0	0	0	0	0	0
OTU54	Metazoa|Annelida|Oligochaeta|Tubificidae	2.01	1.95	0	0	0	0	0	1.06	0	0
OTU55	Metazoa|Platyhelminthes	0	0	0	0	0	0	0	0	0	0
OTU56	Metazoa|Platyhelminthes	0	0	0	0	0	0	0	0	0	0
OTU58	Metazoa|Nematoda	3.7	0	0	6.19	3.22	0	3.5	0	0	0

OTU59	Metazoa|Echinodermata|Echinodermata_X|Echinodermata_XX|Amphiura	0	0	0	0	0	0	0	0	0	0
OTU60	Metazoa|Platyhelminthes	0	0	0	0	0	0	0	0	0	0
OTU61	Metazoa|Cnidaria|Cnidaria_X|Hydrozoa	0	0	0	0	0	0	0	0	0	0
OTU62	Metazoa|Platyhelminthes	0	0	0	0	0	0	0	0	0	0
OTU64	Metazoa|Arthropoda|Crustacea|Maxillopoda|Copepoda	0	0	0	0	0	0	0	0	0	0
OTU65	Metazoa|Nematoda	0	0	0	0	0	0	0	0	0	0
OTU66	Metazoa|Arthropoda|Crustacea|Maxillopoda|Copepoda	0	0	0	0	0	0	0	0	0	0
OTU68	Metazoa|Platyhelminthes	0	0	0	0	0	0	0	0	0	0
OTU69	Metazoa|Platyhelminthes	0	0	0	0	0	0	0	0	0	0
OTU70	Metazoa|Arthropoda|Crustacea|Maxillopoda|Copepoda	0	0	0	0	0	0	0	0	0	0
OTU71	Metazoa|Platyhelminthes	0	0	0	0	0	0	0	0	0	0
OTU72	Metazoa|Nematoda	0	0	0	3.33	0	0	0	0	0	0
OTU73	Metazoa|Nematoda	2.95	0	0	9.28	4.74	0	0	2.6	0	0
OTU75	Metazoa|Gastrotricha	0	0	0	0	0	0	0	0	0	0
OTU76	Metazoa|Arthropoda|Crustacea|Maxillopoda|Copepoda	0	0	0	0	0	0	0	0	0	0
OTU78	Metazoa|Mollusca|Gastropoda|Heterobranchia|Calliopaea|Calliopaea+bellula	19.32	0	0	0	0	0	0	0	0	0
OTU79	Metazoa|Annelida|Phyllodocida|Syllidae	0	0	0	0	0	0	7.31	0	0	0
OTU80	Metazoa|Echinodermata|Echinodermata_X|Echinodermata_XX|Leptosynapta	0	0	0	0	0	0	0	0	0	0
OTU82	Metazoa|Annelida|Capitellida|Capitellidae|Capitella	0	0	0	0	0	3.23	0	0	0	0
OTU84	Metazoa|Annelida|Capitellida|Capitellidae|Capitella	0	0	0	0	0	0	0	0	0	0
OTU85	Metazoa|Platyhelminthes	0	0	0	0	0	0	0	0	0	0
OTU86	Metazoa|Platyhelminthes	0	0	0	0	0	0	0	0	0	0
OTU87	Metazoa|Annelida|Terebellida|Terebellidae	0	0	0	0	0	0	0	0	0	0
OTU88	Metazoa|Platyhelminthes	0	0	0	0	0	0	0	0	0	0
OTU89	Metazoa|Annelida|Oligochaeta|Tubificidae	0	5.21	0	11.17	3.29	0	6.05	0	0	0
OTU90	Metazoa|Cnidaria|Cnidaria_X|Anthozoa|Ceriantheopsis	0	0	0	0	0	0	0	0	0	0
OTU91	Metazoa|Annelida|Capitellida|Scalibregmatidae|Scalibregma|Scalibregma+inflatum	0	0	0	0	0	0	0	0	0	0
OTU92	Metazoa|Annelida|Scolecida|Opheliidae|Ophelina	0	0	0	0	0	0	0	0	0	0
OTU93	Metazoa|Gastrotricha	0	0	0	0	0	0	0	0	0	0

OTU94	Metazoa|Platyhelminthes	0	0	0	0	0	0	0	0	0	0
OTU95	Metazoa|Arthropoda|Crustacea|Ostracoda	0	0	0	0	0	0	0	0	0	0
OTU96	Metazoa|Platyhelminthes	0	0	0	0	0	0	0	0	0	0
OTU97	Metazoa|Platyhelminthes	0	0	0	0	0	0	0	0	0	0
OTU98	Metazoa|Platyhelminthes	0	0	0	0	0	0	0	0	0	0
OTU99	Metazoa|Platyhelminthes	0	0	0	0	0	0	0	0	0	0
OTU100	Metazoa|Arthropoda|Crustacea|Ostracoda	0	0	0	0	0	0	0	0	0	0
OTU101	Metazoa|Annelida|Phyllodocida|Phyllodocidae	0	0	0	0	0	0	0	0	0	0
OTU102	Metazoa|Arthropoda|Crustacea|Maxillopoda|Copepoda	0	0	0	0	0	0	0	0	0	0
OTU103	Metazoa|Nemertea	0	0	0	0	0	0	0	0	0	0
OTU104	Metazoa|Annelida|Phyllodocida|Syllidae	0	0	0	0	0	0	0	0	0	0
OTU107	Metazoa|Arthropoda|Crustacea|Ostracoda	0	0	0	0	0	0	0	0	0	0
OTU108	Metazoa|Annelida|Spionida|Spionidae|Malacoceros|Malacoceros+fuliginosus	0	0	0	0	0	0	0	0	0	0
OTU109	Metazoa|Arthropoda|Crustacea|Maxillopoda|Copepoda	0	0	0	0	0	0	0	0	0	0
OTU110	Metazoa|Arthropoda|Crustacea|Maxillopoda|Copepoda	0	0	0	0	0	0	0	0	0	0
OTU111	Metazoa|Kinorhyncha	0	0	0	0	0	0	0	0	0	0
OTU112	Metazoa|Platyhelminthes	0	0	0	0	0	0	0	0	0	0
OTU113	Metazoa|Nematoda	0	0	0	0	0	0	0	0	0	0
OTU114	Metazoa|Nematoda	0	0	0	0	0	0	0	0	0	0
OTU115	Metazoa	0	0	0	0	0	0	0	0	0	0
OTU116	Metazoa|Platyhelminthes	0	0	0	0	0	0	0	0	0	0
OTU117	Metazoa|Nematoda	0	0	0	0	0	0	0	0	0	0
OTU118	Metazoa|Arthropoda|Crustacea|Maxillopoda|Copepoda	0	0	0	0	0	0	0	0	0	0
OTU119	Metazoa|Nematoda	0	0	0	0	0	0	0	0	0	0
OTU120	Metazoa|Platyhelminthes	0	0	0	0	0	0	0	0	0	0
OTU121	Metazoa|Nematoda	0	0	0	0	0	0	0	0	0	0
OTU122	Metazoa|Annelida|Oligochaeta|Tubificidae	0	0	0	0	1.49	0	0	0	0	0
OTU123	Metazoa|Mollusca|Bivalvia|Heteroconchia|Myrtea|Myrtea+spinifera	0	0	0	0	0	0	0	0	0	0
OTU124	Metazoa|Annelida|Terebellida|Pectinariidae|Pectinaria	0	0	0	0	0	0	0	0	0	0

OTU125	Metazoa|Platyhelminthes	0	0	0	0	0	0	0	0	0	0
OTU126	Metazoa|Nematoda	0	0	0	0	0	0	0	0	0	0
OTU128	Metazoa|Kinorhyncha	0	0	0	0	0	0	0	0	0	0
OTU130	Metazoa|Annelida|Phyllodocida|Phyllodocidae	0	0	0	0	0	0	0	0	0	0
OTU131	Metazoa|Annelida|Oligochaeta|Tubificidae	0	0	0	0	4.42	0	0	0	0	0
OTU132	Metazoa|Nematoda	0	0	0	0	0	0	0	0	0	0
OTU134	Metazoa|Annelida|Phyllodocida|Syllidae	0	0	0	0	0	0	0	0	0	0
OTU135	Metazoa|Annelida|Capitellida|Capitellidae|Capitella	0	0	0	0	0	0	0	0	0	0
OTU136	Metazoa|Nematoda	0	0	0	0	0	0	0	0	0	0
OTU137	Metazoa|Hemichordata	0	0	0	0	0	0	0	0	0	0
OTU138	Metazoa	0	0	0	0	0	0	0	0	0	0
OTU141	Metazoa|Nematoda	0	0	0	0	3.27	0	0	0	0	0
OTU143	Metazoa|Annelida|Capitellida|Capitellidae|Capitella	0	0	0	0	0	0	0	0	0	0
OTU144	Metazoa|Platyhelminthes	0	0	0	0	0	0	0	0	0	0
OTU145	Metazoa|Annelida|Spionida|Spionidae|Malacoceros|Malacoceros+fuliginosus	0	0	0	0	0	0	0	0	0	0
OTU146	Metazoa|Arthropoda|Crustacea|Maxillopoda|Copepoda	0	0	0	0	0	0	0	0	0	0
OTU147	Metazoa|Porifera	0	0	0	0	0	0	0	0	0	0
OTU149	Metazoa|Mollusca|Bivalvia|Heteroconchia|Macoma|Macoma+nasuta	0	0	0	0	0	0	0	0	0	0
OTU151	Metazoa|Nematoda	0	0	0	0	0	0	0	0	0	0
OTU152	Metazoa|Platyhelminthes	0	0	0	0	0	0	0	0	0	0
OTU153	Metazoa|Annelida|Phyllodocida|Phyllodocidae	0	0	0	0	0	0	0	0	0	0
OTU154	Metazoa|Annelida|Spionida|Cirratulidae	0	0	0	0	0	0	0	0	0	0
OTU155	Metazoa|Annelida|Spionida|Cirratulidae	0	0	0	0	0	0	0	0	0	0
OTU156	Metazoa|Arthropoda|Crustacea|Maxillopoda|Copepoda	0	0	0	0	0	0	0	0	0	0
OTU157	Metazoa|Annelida|Phyllodocida|Phyllodocidae	0	0	0	0	0	0	0	0	0	0
OTU158	Metazoa|Annelida|Phyllodocida|Phyllodocidae	0	0	0	0	0	0	0	0	0	0
OTU159	Metazoa|Rotifera	0	0	0	1.91	0	0	0	0	0	0
OTU160	Metazoa|Arthropoda|Crustacea|Maxillopoda|Copepoda	0	0	0	0	0	0	0	0	0	0
OTU161	Metazoa|Platyhelminthes	0	0	0	0	0	0	0	0	0	0

OTU162	Metazoa|Annelida|Capitellida|Capitellidae|Capitella	0	0	0	0	0	0	0	0	0	0
OTU163	Metazoa|Nematoda	0	0	0	0	0	0	0	0	0	0
OTU164	Metazoa|Nematoda	0	0	0	0	0	0	0	0	0	0
OTU165	Metazoa|Nematoda	0	0	0	0	0	0	0	0	0	0
OTU167	Metazoa|Nematoda	0	0	0	0	0	0	0	0	0	0
OTU168	Metazoa|Cnidaria|Cnidaria_X|Hydrozoa	0	0	0	0	0	0	0	0	0	0
OTU169	Metazoa|Arthropoda|Crustacea|Maxillopoda|Copepoda	0	0	0	0	0	0	0	0	0	0
OTU170	Metazoa|Gastrotricha	0	0	0	0	0	0	0	0	0	0
OTU171	Metazoa|Annelida|Phyllodocida|Syllidae	0	0	0	0	0	0	0	0	0	0
OTU173	Metazoa|Nematoda	0	0	0	0	0	0	0	0	0	0
OTU174	Metazoa|Arthropoda|Crustacea|Maxillopoda|Copepoda	0	0	0	0	0	0	0	0	0	0
OTU175	Metazoa|Arthropoda|Crustacea|Maxillopoda|Copepoda	0	0	0	0	0	0	0	0	0	0
OTU176	Metazoa|Platyhelminthes	0	0	0	0	0	0	0	0	0	0
OTU178	Metazoa|Nematoda	0	0	0	3.08	0	0	0	0	0	0
OTU180	Metazoa|Nematoda	0	0	0	0	0	0	0	0	0	0
OTU183	Metazoa|Annelida|Oligochaeta|Tubificidae	0	0	0	0	0	0	0	0	0	0
OTU184	Metazoa|Nematoda	0	0	0	0	0	0	0	0	0	0
OTU186	Metazoa|Platyhelminthes	0	0	0	0	0	0	0	0	0	0
OTU188	Metazoa|Annelida|Terebellida|Ampharetidae	0	0	0	0	0	0	0	0	0	0
OTU190	Metazoa|Annelida|Terebellida|Terebellidae	0	0	0	0	0	0	0	0	0	0
OTU191	Metazoa|Annelida|Phyllodocida|Syllidae	0	0	0	0	0	0	0	0	0	0
OTU192	Metazoa|Annelida|Spionida|Cirratulidae	0	0	0	0	0	0	2.42	0	0	0

Supplementary Table 7. continued

OTU	assign	DNA_6_1	DNA_6_2	DNA_6_3	DNA_6_4	DNA_6_5	DNA_7_1	DNA_7_2	DNA_7_3	DNA_7_4	DNA_7_5
OTU1	Metazoa|Annelida|Capitellida|Capitellidae|Capitella	2644.65	2244.77	3662.77	1748.11	2391.33	0	0	0	0	0
OTU2	Metazoa|Annelida|Oligochaeta|Tubificidae	515.22	329.37	107.01	1070.22	785.85	0	0	0	0	0
OTU4	Metazoa|Annelida|Phyllodocida|Phyllodocidae|Phyllodoce|Phyllodoce+maculata	5.36	92.02	0	676.28	254.26	0	2175.44	0	0	0
OTU5	Metazoa|Annelida|Spionida|Spionidae|Malacoceros|Malacoceros+fuliginosus	1.38	0	0	0	0	0	0	0	0	0
OTU6	Metazoa|Annelida|Spionida|Cirratulidae	0	0	0	0	0	0	0	0	0	0
OTU7	Metazoa|Platyhelminthes	0	193.31	21	0	30.14	23.39	32.04	2533.29	0	0
OTU8	Metazoa|Annelida|Phyllodocida|Syllidae|Salvatoria	262.72	671.46	0	183.56	44.56	0	0	0	7.73	0
OTU9	Metazoa|Annelida	43.13	81.51	32.16	236.24	191.18	827.65	1248.42	0	795.49	2438.83
OTU10	Metazoa|Nematoda	0	0	0	0	0	0	0	0	0	0
OTU11	Metazoa|Nematoda	16.43	0	0	0	0	0	0	0	0	0
OTU12	Metazoa|Nematoda	48.51	306.94	147.37	107.7	118.28	0	0	0	0	0
OTU13	Metazoa|Cnidaria|Cnidaria_X|Anthozoa	0	0	0	0	0	0	0	0	0	0
OTU14	Metazoa|Nemertea	0	0	0	0	0	0	197.7	0	0	0
OTU15	Metazoa|Mollusca|Bivalvia|Heteroconchia|Abra	0	0	0	0	0	0	0	0	0	0
OTU16	Metazoa|Platyhelminthes	0	0	0	0	0	179.71	15.18	64.5	8.42	256.42
OTU17	Metazoa|Cnidaria|Cnidaria_X|Hydrozoa	0	0	0	0	0	0	0	22.2	24.26	0
OTU18	Metazoa|Platyhelminthes	7.7	24.16	34.9	55.04	141.54	224.18	0	418.56	0	0
OTU19	Metazoa|Arthropoda|Crustacea|Ostracoda	0	0	0	0	0	148.25	4.02	0	69.92	382.29
OTU20	Metazoa|Nematoda	19.23	67.33	43.54	15.54	43.77	0	0	0	0	0
OTU21	Metazoa|Annelida|Terebellida|Ampharetidae	0	0	0	0	0	0	0	0	2630.81	0
OTU22	Metazoa|Annelida|Phyllodocida|Syllidae|Exogone|Exogone+naidina	0	0	0	0	0	0	0	0	0	0
OTU23	Metazoa|Platyhelminthes	0	0	0	0	0	131.8	6.72	28.74	0	386.33
OTU24	Metazoa|Platyhelminthes	0	0	0	0	0	0	0	0	0	0
OTU25	Metazoa|Mollusca|Bivalvia|Heteroconchia|Corbula	0	0	0	0	0	0	0	0	0	0
OTU26	Metazoa|Annelida|Capitellida|Capitellidae|Heteromastus	0	0	0	0	0	0	0	0	0	0

OTU27	Metazoa|Arthropoda|Crustacea|Maxillopoda|Copepoda	0	0	0	0	0	154.16	119.45	667.66	71.03	638.13
OTU28	Metazoa|Nematoda	0	0	0	0	0	0	0	0	0	0
OTU29	Metazoa|Cnidaria|Cnidaria_X|Hydrozoa	0	4.82	0	0	0	1842.18	0	0	0	0
OTU30	Metazoa|Mollusca|Gastropoda|Heterobranchia|Bulla	0	0	0	0	0	177.22	0	0	0	0
OTU31	Metazoa|Platyhelminthes	0	0	0	0	0	66.59	24.3	93	28.55	0
OTU32	Metazoa|Arthropoda|Crustacea|Ostracoda	0	0	0	0	0	0	0	0	0	0
OTU33	Metazoa|Nemertea	0	0	0	0	0	0	210.16	0	0	0
OTU35	Metazoa|Nemertea	0	0	0	0	0	0	0	179.56	0	0
OTU36	Metazoa|Arthropoda|Chelicerata	0	0	0	0	0	0	18.05	0	0	0
OTU37	Metazoa|Annelida|Phyllodocida|Syllidae|Erinaceusyllis|Erinaceusyllis+cryptica	0	7.16	0	0	17.66	0	0	0	0	0
OTU38	Metazoa|Platyhelminthes	442.89	0	0	0	0	0	0	0	0	0
OTU39	Metazoa|Arthropoda|Crustacea|Maxillopoda|Copepoda	0	0	0	0	0	0	0	0	0	0
OTU40	Metazoa|Annelida|Capitellida|Maldanidae|Praxillella	0	0	0	0	0	0	0	0	0	0
OTU41	Metazoa|Arthropoda|Crustacea|Maxillopoda|Copepoda	0	0	0	0	0	0	0	0	0	0
OTU42	Metazoa|Annelida|Phyllodocida|Syllidae|Exogone|Exogone+naidina	0	0	41.02	0	79.3	0	0	0	0	0
OTU43	Metazoa|Hemichordata	0	0	0	0	0	0	13.07	0	0	0
OTU44	Metazoa|Cnidaria|Cnidaria_X|Hydrozoa	0	0	0	0	0	0	0	0	16.22	0
OTU45	Metazoa|Mollusca|Bivalvia|Protobranchia|Nucula	3.25	0	0	0	0	0	0	0	7.99	0
OTU46	Metazoa|Annelida|Capitellida|Capitellidae|Capitella	0	0	0	0	0	0	0	0	0	0
OTU47	Metazoa|Platyhelminthes	0	0	0	0	0	20.16	0	0	0	0
OTU48	Metazoa|Platyhelminthes	0	0	0	0	0	5.69	0	0	0	0
OTU49	Metazoa|Platyhelminthes	0	0	0	0	0	0	0	0	0	0
OTU51	Metazoa|Nematoda	0	9.31	0	0	0	0	0	0	0	0
OTU52	Metazoa|Annelida|Capitellida|Maldanidae	0	0	0	0	0	0	0	0	0	0
OTU53	Metazoa|Nematoda	0	0	7.32	0	0	0	0	0	0	0
OTU54	Metazoa|Annelida|Oligochaeta|Tubificidae	0	0	0	0	0	0	0	0	0	0
OTU55	Metazoa|Platyhelminthes	0	0	0	0	0	0	0	0	0	0
OTU56	Metazoa|Platyhelminthes	0	0	0	0	0	0	0	0	0	0
OTU58	Metazoa|Nematoda	2.48	33.88	0	0	0	0	0	0	0	0

OTU59	Metazoa|Echinodermata|Echinodermata_X|Echinodermata_XX|Amphiura	0	0	0	0	0	0	0	0	420.52	0
OTU60	Metazoa|Platyhelminthes	0	0	0	0	0	0	0	0	0	0
OTU61	Metazoa|Cnidaria|Cnidaria_X|Hydrozoa	0	0	0	0	0	0	0	0	8.49	0
OTU62	Metazoa|Platyhelminthes	0	0	0	0	0	0	0	0	0	0
OTU64	Metazoa|Arthropoda|Crustacea|Maxillopoda|Copepoda	0	0	0	0	0	0	0	0	0	0
OTU65	Metazoa|Nematoda	0	0	0	0	0	0	0	0	0	0
OTU66	Metazoa|Arthropoda|Crustacea|Maxillopoda|Copepoda	0	0	0	0	0	0	0	0	0	0
OTU68	Metazoa|Platyhelminthes	0	0	0	0	0	0	30.65	0	0	0
OTU69	Metazoa|Platyhelminthes	84.96	0	0	0	0	0	0	0	0	0
OTU70	Metazoa|Arthropoda|Crustacea|Maxillopoda|Copepoda	0	0	0	0	0	0	0	0	0	0
OTU71	Metazoa|Platyhelminthes	0	0	0	0	0	0	0	0	0	0
OTU72	Metazoa|Nematoda	0	0	0	0	0	0	0	0	0	0
OTU73	Metazoa|Nematoda	4.09	35.96	4.91	0	4.13	0	0	0	0	0
OTU75	Metazoa|Gastrotricha	0	0	0	0	0	0	0	0	0	0
OTU76	Metazoa|Arthropoda|Crustacea|Maxillopoda|Copepoda	0	0	0	0	0	0	2.79	0	0	0
OTU78	Metazoa|Mollusca|Gastropoda|Heterobranchia|Calliopaea|Calliopaea+bellula	0	0	0	0	0	0	0	0	0	0
OTU79	Metazoa|Annelida|Phyllodocida|Syllidae	0	0	0	0	0	0	0	0	0	0
OTU80	Metazoa|Echinodermata|Echinodermata_X|Echinodermata_XX|Leptosynapta	0	0	0	0	0	0	0	0	0	0
OTU82	Metazoa|Annelida|Capitellida|Capitellidae|Capitella	0	0	0	0	0	0	0	0	0	0
OTU84	Metazoa|Annelida|Capitellida|Capitellidae|Capitella	0	0	0	0	0	0	0	0	0	0
OTU85	Metazoa|Platyhelminthes	0	0	0	0	0	0	0	0	0	0
OTU86	Metazoa|Platyhelminthes	0	0	0	0	0	0	0	0	0	0
OTU87	Metazoa|Annelida|Terebellida|Terebellidae	0	0	0	0	0	167.71	0	0	0	0
OTU88	Metazoa|Platyhelminthes	0	0	0	0	0	0	0	0	0	0
OTU89	Metazoa|Annelida|Oligochaeta|Tubificidae	0	0	0	0	0	0	0	0	0	0
OTU90	Metazoa|Cnidaria|Cnidaria_X|Anthozoa|Ceriantheopsis	0	0	0	0	0	0	0	0	0	0
OTU91	Metazoa|Annelida|Capitellida|Scalibregmatidae|Scalibregma|Scalibregma+inflatum	0	0	0	0	0	0	0	0	12.57	0
OTU92	Metazoa|Annelida|Scolecida|Opheliidae|Ophelina	0	0	0	0	0	0	0	0	0	0
OTU93	Metazoa|Gastrotricha	0	0	0	0	0	0	0	14.41	0	0

OTU94	Metazoa|Platyhelminthes	0	0	0	0	0	0	0	0	0	0
OTU95	Metazoa|Arthropoda|Crustacea|Ostracoda	0	0	0	0	0	0	0	0	0	0
OTU96	Metazoa|Platyhelminthes	0	0	0	0	0	14.25	0	0	0	0
OTU97	Metazoa|Platyhelminthes	0	0	0	0	0	0	0	0	0	0
OTU98	Metazoa|Platyhelminthes	0	0	0	0	0	0	0	0	0	0
OTU99	Metazoa|Platyhelminthes	0	0	0	0	0	37.48	0	0	0	0
OTU100	Metazoa|Arthropoda|Crustacea|Ostracoda	0	0	0	0	0	0	0	0	0	0
OTU101	Metazoa|Annelida|Phyllodocida|Phyllodocidae	0	0	0	0	0	0	0	0	0	0
OTU102	Metazoa|Arthropoda|Crustacea|Maxillopoda|Copepoda	0	0	0	0	0	0	0	0	0	0
OTU103	Metazoa|Nemertea	0	0	0	0	0	0	0	0	0	0
OTU104	Metazoa|Annelida|Phyllodocida|Syllidae	0	0	0	0	0	36.9	0	80.08	0	0
OTU107	Metazoa|Arthropoda|Crustacea|Ostracoda	0	0	0	0	0	0	0	0	0	0
OTU108	Metazoa|Annelida|Spionida|Spionidae|Malacoceros|Malacoceros+fuliginosus	0	0	0	0	0	0	0	0	0	0
OTU109	Metazoa|Arthropoda|Crustacea|Maxillopoda|Copepoda	0	0	0	0	0	0	0	0	0	0
OTU110	Metazoa|Arthropoda|Crustacea|Maxillopoda|Copepoda	0	0	0	0	0	0	0	0	0	0
OTU111	Metazoa|Kinorhyncha	0	0	0	0	0	0	0	0	0	0
OTU112	Metazoa|Platyhelminthes	0	0	0	0	0	0	0	0	0	0
OTU113	Metazoa|Nematoda	0	0	0	0	0	0	0	0	0	0
OTU114	Metazoa|Nematoda	0	0	0	0	0	33.26	0	0	0	0
OTU115	Metazoa	0	0	0	0	0	0	0	0	0	0
OTU116	Metazoa|Platyhelminthes	0	0	0	0	0	0	0	0	0	0
OTU117	Metazoa|Nematoda	0	0	0	0	0	0	0	0	0	0
OTU118	Metazoa|Arthropoda|Crustacea|Maxillopoda|Copepoda	0	0	0	0	0	0	0	0	0	0
OTU119	Metazoa|Nematoda	0	0	0	0	0	0	0	0	0	0
OTU120	Metazoa|Platyhelminthes	0	0	0	0	0	0	0	0	0	0
OTU121	Metazoa|Nematoda	0	0	0	0	0	0	0	0	0	0
OTU122	Metazoa|Annelida|Oligochaeta|Tubificidae	0	0	0	9.31	0	0	0	0	0	0
OTU123	Metazoa|Mollusca|Bivalvia|Heteroconchia|Myrtea|Myrtea+spinifera	0	0	0	0	0	0	0	0	0	0
OTU124	Metazoa|Annelida|Terebellida|Pectinariidae|Pectinaria	0	0	0	0	0	0	0	0	0	0

OTU125	Metazoa|Platyhelminthes	0	0	0	0	0	0	0	0	0	0
OTU126	Metazoa|Nematoda	0	0	0	0	0	0	0	0	0	0
OTU128	Metazoa|Kinorhyncha	0	0	0	0	0	0	0	0	0	0
OTU130	Metazoa|Annelida|Phyllodocida|Phyllodocidae	0	0	0	0	0	0	0	0	0	0
OTU131	Metazoa|Annelida|Oligochaeta|Tubificidae	0	0	0	0	0	0	0	0	0	0
OTU132	Metazoa|Nematoda	0	0	0	0	0	0	0	0	0	0
OTU134	Metazoa|Annelida|Phyllodocida|Syllidae	0	0	0	0	0	0	0	0	0	0
OTU135	Metazoa|Annelida|Capitellida|Capitellidae|Capitella	0	0	0	0	0	0	0	0	0	0
OTU136	Metazoa|Nematoda	0	0	0	0	0	0	0	0	0	0
OTU137	Metazoa|Hemichordata	0	0	0	0	0	0	0	0	0	0
OTU138	Metazoa	0	0	0	0	0	0	0	0	0	0
OTU141	Metazoa|Nematoda	0	0	0	0	0	0	0	0	0	0
OTU143	Metazoa|Annelida|Capitellida|Capitellidae|Capitella	0	0	0	0	0	0	0	0	0	0
OTU144	Metazoa|Platyhelminthes	0	0	0	0	0	0	0	0	0	0
OTU145	Metazoa|Annelida|Spionida|Spionidae|Malacoceros|Malacoceros+fuliginosus	0	0	0	0	0	0	0	0	0	0
OTU146	Metazoa|Arthropoda|Crustacea|Maxillopoda|Copepoda	0	0	0	0	0	0	0	0	0	0
OTU147	Metazoa|Porifera	0	0	0	0	0	0	0	0	0	0
OTU149	Metazoa|Mollusca|Bivalvia|Heteroconchia|Macoma|Macoma+nasuta	0	0	0	0	0	0	0	0	0	0
OTU151	Metazoa|Nematoda	0	0	0	0	0	0	0	0	0	0
OTU152	Metazoa|Platyhelminthes	0	0	0	0	0	0	0	0	0	0
OTU153	Metazoa|Annelida|Phyllodocida|Phyllodocidae	0	0	0	0	0	0	0	0	0	0
OTU154	Metazoa|Annelida|Spionida|Cirratulidae	0	0	0	0	0	0	0	0	0	0
OTU155	Metazoa|Annelida|Spionida|Cirratulidae	0	0	0	0	0	0	0	0	0	0
OTU156	Metazoa|Arthropoda|Crustacea|Maxillopoda|Copepoda	0	0	0	0	0	0	0	0	0	0
OTU157	Metazoa|Annelida|Phyllodocida|Phyllodocidae	0	0	0	0	0	0	0	0	0	0
OTU158	Metazoa|Annelida|Phyllodocida|Phyllodocidae	0	0	0	0	0	0	0	0	0	0
OTU159	Metazoa|Rotifera	0	0	0	0	0	0	0	0	0	0
OTU160	Metazoa|Arthropoda|Crustacea|Maxillopoda|Copepoda	0	0	0	0	0	0	0	0	0	0
OTU161	Metazoa|Platyhelminthes	0	0	0	0	0	0	0	0	0	0

OTU162	Metazoa|Annelida|Capitellida|Capitellidae|Capitella	0	0	0	0	0	0	0	0	0	0
OTU163	Metazoa|Nematoda	0	0	0	0	0	0	0	0	0	0
OTU164	Metazoa|Nematoda	0	0	0	0	0	5.72	4.01	0	0	0
OTU165	Metazoa|Nematoda	0	0	0	0	0	0	0	0	0	0
OTU167	Metazoa|Nematoda	0	0	0	0	0	0	0	0	0	0
OTU168	Metazoa|Cnidaria|Cnidaria_X|Hydrozoa	0	0	0	0	0	0	0	0	0	0
OTU169	Metazoa|Arthropoda|Crustacea|Maxillopoda|Copepoda	0	0	0	0	0	0	0	0	0	0
OTU170	Metazoa|Gastrotricha	0	0	0	0	0	0	0	0	0	0
OTU171	Metazoa|Annelida|Phyllodocida|Syllidae	0	0	0	0	0	0	0	0	0	0
OTU173	Metazoa|Nematoda	0	0	0	0	0	0	0	0	0	0
OTU174	Metazoa|Arthropoda|Crustacea|Maxillopoda|Copepoda	0	0	0	0	0	0	0	0	0	0
OTU175	Metazoa|Arthropoda|Crustacea|Maxillopoda|Copepoda	0	0	0	0	0	0	0	0	0	0
OTU176	Metazoa|Platyhelminthes	0	0	0	0	0	0	0	0	0	0
OTU178	Metazoa|Nematoda	0	0	0	0	0	0	0	0	0	0
OTU180	Metazoa|Nematoda	0	0	0	0	0	0	0	0	0	0
OTU183	Metazoa|Annelida|Oligochaeta|Tubificidae	0	0	0	0	0	0	0	0	0	0
OTU184	Metazoa|Nematoda	0	0	0	0	0	0	0	0	0	0
OTU186	Metazoa|Platyhelminthes	0	0	0	0	0	0	0	0	0	0
OTU188	Metazoa|Annelida|Terebellida|Ampharetidae	0	0	0	0	0	0	0	0	0	0
OTU190	Metazoa|Annelida|Terebellida|Terebellidae	0	0	0	0	0	5.7	0	0	0	0
OTU191	Metazoa|Annelida|Phyllodocida|Syllidae	0	0	0	0	0	0	0	0	0	0
OTU192	Metazoa|Annelida|Spionida|Cirratulidae	0	0	0	0	0	0	0	0	0	0

Supplementary Table 7. continued

OTU	assign	DNA_8_1	DNA_8_2	DNA_8_3	DNA_8_4	DNA_8_5	DNA_9_1	DNA_9_2	DNA_9_3	DNA_9_4	DNA_9_5
OTU1	Metazoa|Annelida|Capitellida|Capitellidae|Capitella	0	0	28.58	0	1482.61	12.79	0	0	0	0
OTU2	Metazoa|Annelida|Oligochaeta|Tubificidae	0	0	0	0	0	0	0	0	0	0
OTU4	Metazoa|Annelida|Phyllodocida|Phyllodocidae|Phyllodoce|Phyllodoce+maculata	0	0	0	2852.49	1087.12	0	0	0	0	0

OTU5	Metazoa|Annelida|Spionida|Spionidae|Malacoceros|Malacoceros+fuliginosus	0	0	0	0	0	0	0	0	0	0
OTU6	Metazoa|Annelida|Spionida|Cirratulidae	0	0	0	0	0	0	0	0	0	0
OTU7	Metazoa|Platyhelminthes	339.48	0	0	24.06	655.84	0	0	749.79	0	911.79
OTU8	Metazoa|Annelida|Phyllodocida|Syllidae|Salvatoria	0	0	0	0	0	0	0	621.76	0	841.78
OTU9	Metazoa|Annelida	0	2825.47	0	0	0	0	0	0	0	0
OTU10	Metazoa|Nematoda	0	0	0	0	0	0	0	0	0	0
OTU11	Metazoa|Nematoda	0	0	0	0	0	0	0	0	0	0
OTU12	Metazoa|Nematoda	0	0	43.52	0	0	13.69	0	0	0	138.89
OTU13	Metazoa|Cnidaria|Cnidaria_X|Anthozoa	0	0	0	0	0	0	0	0	0	0
OTU14	Metazoa|Nemertea	0	0	0	620.2	0	0	0	0	0	0
OTU15	Metazoa|Mollusca|Bivalvia|Heteroconchia|Abra	0	0	0	0	0	0	0	864.96	0	0
OTU16	Metazoa|Platyhelminthes	94.14	207.19	112.97	47.56	0	13.64	0	0	0	0
OTU17	Metazoa|Cnidaria|Cnidaria_X|Hydrozoa	527.94	0	1061.25	0	270.05	156.73	831.51	0	0	0
OTU18	Metazoa|Platyhelminthes	0	0	0	0	0	881.95	0	0	0	0
OTU19	Metazoa|Arthropoda|Crustacea|Ostracoda	467.58	92.36	87.61	23.63	64.16	0	2595.9	68.16	0	138.44
OTU20	Metazoa|Nematoda	0	0	0	0	0	0	0	0	0	0
OTU21	Metazoa|Annelida|Terebellida|Ampharetidae	0	0	0	0	0	0	0	0	0	0
OTU22	Metazoa|Annelida|Phyllodocida|Syllidae|Exogone|Exogone+naidina	0	0	0	0	0	0	0	0	0	0
OTU23	Metazoa|Platyhelminthes	406.01	0	70.97	8.81	0	0	208.36	329.76	80.03	0
OTU24	Metazoa|Platyhelminthes	0	0	0	0	0	0	0	0	0	0
OTU25	Metazoa|Mollusca|Bivalvia|Heteroconchia|Corbula	0	0	0	0	0	0	0	0	0	0
OTU26	Metazoa|Annelida|Capitellida|Capitellidae|Heteromastus	0	0	99.36	0	126.35	0	0	0	0	0
OTU27	Metazoa|Arthropoda|Crustacea|Maxillopoda|Copepoda	1426.81	763.03	990.76	206.09	415.87	540.52	163.34	1269.31	731.22	1055.14
OTU28	Metazoa|Nematoda	0	0	0	0	0	0	0	0	0	0
OTU29	Metazoa|Cnidaria|Cnidaria_X|Hydrozoa	0	130.5	1232.37	0	0	0	0	0	0	0
OTU30	Metazoa|Mollusca|Gastropoda|Heterobranchia|Bulla	0	0	115.51	0	0	0	0	0	0	0
OTU31	Metazoa|Platyhelminthes	0	0	56.83	69.64	0	0	29.62	0	0	0
OTU32	Metazoa|Arthropoda|Crustacea|Ostracoda	0	0	0	0	0	0	0	0	0	0
OTU33	Metazoa|Nemertea	0	0	0	0	0	0	0	43.37	3122.16	0

OTU35	Metazoa|Nemertea	0	0	0	59.42	0	0	0	0	0	0
OTU36	Metazoa|Arthropoda|Chelicerata	122.9	0	0	0	0	0	86.09	0	0	0
OTU37	Metazoa|Annelida|Phyllodocida|Syllidae|Erinaceusyllis|Erinaceusyllis+cryptica	0	0	0	0	0	0	0	0	0	0
OTU38	Metazoa|Platyhelminthes	0	0	0	0	0	0	0	0	0	0
OTU39	Metazoa|Arthropoda|Crustacea|Maxillopoda|Copepoda	0	0	0	0	0	0	0	0	0	0
OTU40	Metazoa|Annelida|Capitellida|Maldanidae|Praxillella	0	0	0	0	0	2393.93	0	0	0	0
OTU41	Metazoa|Arthropoda|Crustacea|Maxillopoda|Copepoda	0	0	0	0	0	0	0	0	0	0
OTU42	Metazoa|Annelida|Phyllodocida|Syllidae|Exogone|Exogone+naidina	0	0	0	0	0	0	0	0	0	0
OTU43	Metazoa|Hemichordata	0	0	0	0	0	0	0	0	0	0
OTU44	Metazoa|Cnidaria|Cnidaria_X|Hydrozoa	0	0	0	0	0	0	86.21	0	0	0
OTU45	Metazoa|Mollusca|Bivalvia|Protobranchia|Nucula	0	0	0	145.47	0	0	0	0	0	0
OTU46	Metazoa|Annelida|Capitellida|Capitellidae|Capitella	0	0	0	0	0	0	0	0	0	0
OTU47	Metazoa|Platyhelminthes	0	0	0	0	0	0	0	0	0	0
OTU48	Metazoa|Platyhelminthes	0	0	0	4.74	0	0	0	0	0	0
OTU49	Metazoa|Platyhelminthes	0	0	0	0	0	0	0	0	0	0
OTU51	Metazoa|Nematoda	0	0	0	0	0	0	0	0	0	0
OTU52	Metazoa|Annelida|Capitellida|Maldanidae	0	0	0	0	0	88.75	0	0	0	0
OTU53	Metazoa|Nematoda	0	0	0	0	0	0	0	0	0	0
OTU54	Metazoa|Annelida|Oligochaeta|Tubificidae	0	0	0	0	0	0	0	0	0	0
OTU55	Metazoa|Platyhelminthes	0	0	0	0	0	0	0	0	0	0
OTU56	Metazoa|Platyhelminthes	0	0	0	0	0	0	0	0	0	0
OTU58	Metazoa|Nematoda	126.14	0	0	0	0	0	0	0	0	0
OTU59	Metazoa|Echinodermata|Echinodermata_X|Echinodermata_XX|Amphiura	0	0	0	0	0	0	0	0	0	0
OTU60	Metazoa|Platyhelminthes	247.56	0	0	0	0	0	0	0	0	0
OTU61	Metazoa|Cnidaria|Cnidaria_X|Hydrozoa	249.65	0	0	0	0	0	0	0	0	0
OTU62	Metazoa|Platyhelminthes	0	0	0	0	0	0	0	0	0	0
OTU64	Metazoa|Arthropoda|Crustacea|Maxillopoda|Copepoda	0	0	0	0	0	0	0	0	0	0
OTU65	Metazoa|Nematoda	0	0	0	0	0	0	0	0	0	0
OTU66	Metazoa|Arthropoda|Crustacea|Maxillopoda|Copepoda	0	0	0	0	0	0	0	66.23	34.99	0

OTU68	Metazoa|Platyhelminthes	0	0	0	0	0	0	0	0	0	0
OTU69	Metazoa|Platyhelminthes	0	0	0	0	0	0	0	0	0	0
OTU70	Metazoa|Arthropoda|Crustacea|Maxillopoda|Copepoda	0	0	0	0	0	0	0	0	0	0
OTU71	Metazoa|Platyhelminthes	0	0	0	0	0	0	0	0	0	0
OTU72	Metazoa|Nematoda	0	0	0	0	0	0	0	0	0	0
OTU73	Metazoa|Nematoda	93.79	83.45	0	0	0	0	0	0	0	0
OTU75	Metazoa|Gastrotricha	0	0	0	0	0	0	0	0	0	0
OTU76	Metazoa|Arthropoda|Crustacea|Maxillopoda|Copepoda	0	0	0	0	0	0	14.58	45.02	81.62	69.19
OTU78	Metazoa|Mollusca|Gastropoda|Heterobranchia|Calliopaea|Calliopaea+bellula	0	0	0	0	0	0	0	0	0	0
OTU79	Metazoa|Annelida|Phyllodocida|Syllidae	0	0	0	0	0	0	0	0	0	0
OTU80	Metazoa|Echinodermata|Echinodermata_X|Echinodermata_XX|Leptosynapta	0	0	0	0	0	0	0	0	0	0
OTU82	Metazoa|Annelida|Capitellida|Capitellidae|Capitella	0	0	0	0	0	0	0	0	0	0
OTU84	Metazoa|Annelida|Capitellida|Capitellidae|Capitella	0	0	0	0	0	0	0	0	0	0
OTU85	Metazoa|Platyhelminthes	0	0	0	0	0	0	0	0	0	0
OTU86	Metazoa|Platyhelminthes	0	0	0	0	0	0	0	0	0	0
OTU87	Metazoa|Annelida|Terebellida|Terebellidae	0	0	0	0	0	0	0	0	0	0
OTU88	Metazoa|Platyhelminthes	0	0	0	0	0	0	0	0	0	0
OTU89	Metazoa|Annelida|Oligochaeta|Tubificidae	0	0	0	0	0	0	0	0	0	0
OTU90	Metazoa|Cnidaria|Cnidaria_X|Anthozoa|Ceriantheopsis	0	0	0	0	0	0	0	0	0	0
OTU91	Metazoa|Annelida|Capitellida|Scalibregmatidae|Scalibregma|Scalibregma+inflatum	0	0	0	0	0	0	0	0	0	0
OTU92	Metazoa|Annelida|Scolecida|Opheliidae|Ophelina	0	0	0	0	0	0	0	0	0	0
OTU93	Metazoa|Gastrotricha	0	0	0	0	0	0	0	0	0	0
OTU94	Metazoa|Platyhelminthes	0	0	130.47	0	0	0	0	0	0	0
OTU95	Metazoa|Arthropoda|Crustacea|Ostracoda	0	0	0	0	0	0	0	0	0	0
OTU96	Metazoa|Platyhelminthes	0	0	0	0	0	0	0	0	0	0
OTU97	Metazoa|Platyhelminthes	0	0	0	0	0	0	0	0	0	0
OTU98	Metazoa|Platyhelminthes	0	0	0	0	0	0	0	0	0	351.06
OTU99	Metazoa|Platyhelminthes	0	0	0	0	0	0	0	0	0	0
OTU100	Metazoa|Arthropoda|Crustacea|Ostracoda	0	0	0	0	0	0	0	0	0	0

OTU101	Metazoa|Annelida|Phyllodocida|Phyllodocidae	0	0	0	0	0	0	0	0	0	0
OTU102	Metazoa|Arthropoda|Crustacea|Maxillopoda|Copepoda	0	0	0	0	0	0	0	43.64	0	0
OTU103	Metazoa|Nemertea	0	0	0	5.88	0	0	0	0	0	0
OTU104	Metazoa|Annelida|Phyllodocida|Syllidae	0	0	0	0	0	0	0	0	0	0
OTU107	Metazoa|Arthropoda|Crustacea|Ostracoda	0	0	0	0	0	0	0	0	0	0
OTU108	Metazoa|Annelida|Spionida|Spionidae|Malacoceros|Malacoceros+fuliginosus	0	0	0	0	0	0	0	0	0	0
OTU109	Metazoa|Arthropoda|Crustacea|Maxillopoda|Copepoda	0	0	0	0	0	0	0	0	0	0
OTU110	Metazoa|Arthropoda|Crustacea|Maxillopoda|Copepoda	0	0	0	0	0	0	0	0	0	0
OTU111	Metazoa|Kinorhyncha	0	0	0	0	0	0	0	0	0	0
OTU112	Metazoa|Platyhelminthes	0	0	28.92	0	0	0	0	0	0	0
OTU113	Metazoa|Nematoda	0	0	42.88	5.74	0	0	0	0	0	0
OTU114	Metazoa|Nematoda	0	0	0	0	0	0	0	0	0	0
OTU115	Metazoa	0	0	0	0	0	0	0	0	0	0
OTU116	Metazoa|Platyhelminthes	0	0	0	0	0	0	0	0	0	0
OTU117	Metazoa|Nematoda	0	0	0	0	0	0	86.39	0	0	0
OTU118	Metazoa|Arthropoda|Crustacea|Maxillopoda|Copepoda	0	0	0	0	0	0	0	0	0	0
OTU119	Metazoa|Nematoda	0	0	0	0	0	0	0	0	0	0
OTU120	Metazoa|Platyhelminthes	0	0	0	0	0	0	0	0	0	316.4
OTU121	Metazoa|Nematoda	0	0	0	22.05	0	0	0	0	0	0
OTU122	Metazoa|Annelida|Oligochaeta|Tubificidae	0	0	0	0	0	0	0	0	0	0
OTU123	Metazoa|Mollusca|Bivalvia|Heteroconchia|Myrtea|Myrtea+spinifera	0	0	0	0	0	0	0	0	0	0
OTU124	Metazoa|Annelida|Terebellida|Pectinariidae|Pectinaria	0	0	0	0	0	0	0	0	0	0
OTU125	Metazoa|Platyhelminthes	0	0	0	0	0	0	0	0	0	0
OTU126	Metazoa|Nematoda	0	0	0	0	0	0	0	0	0	0
OTU128	Metazoa|Kinorhyncha	0	0	0	0	0	0	0	0	0	0
OTU130	Metazoa|Annelida|Phyllodocida|Phyllodocidae	0	0	0	6.22	0	0	0	0	0	0
OTU131	Metazoa|Annelida|Oligochaeta|Tubificidae	0	0	0	0	0	0	0	0	0	0
OTU132	Metazoa|Nematoda	0	0	0	0	0	0	0	0	0	0
OTU134	Metazoa|Annelida|Phyllodocida|Syllidae	0	0	0	0	0	0	0	0	0	0

OTU135	Metazoa|Annelida|Capitellida|Capitellidae|Capitella	0	0	0	0	0	0	0	0	0	0
OTU136	Metazoa|Nematoda	0	0	0	0	0	0	0	0	0	0
OTU137	Metazoa|Hemichordata	0	0	0	0	0	0	0	0	0	0
OTU138	Metazoa	0	0	0	0	0	0	0	0	0	0
OTU141	Metazoa|Nematoda	0	0	0	0	0	0	0	0	0	0
OTU143	Metazoa|Annelida|Capitellida|Capitellidae|Capitella	0	0	0	0	0	0	0	0	0	0
OTU144	Metazoa|Platyhelminthes	0	0	0	0	0	0	0	0	0	0
OTU145	Metazoa|Annelida|Spionida|Spionidae|Malacoceros|Malacoceros+fuliginosus	0	0	0	0	0	0	0	0	0	0
OTU146	Metazoa|Arthropoda|Crustacea|Maxillopoda|Copepoda	0	0	0	0	0	0	0	0	0	0
OTU147	Metazoa|Porifera	0	0	0	0	0	0	0	0	0	0
OTU149	Metazoa|Mollusca|Bivalvia|Heteroconchia|Macoma|Macoma+nasuta	0	0	0	0	0	0	0	0	0	0
OTU151	Metazoa|Nematoda	0	0	0	0	0	0	0	0	51.98	0
OTU152	Metazoa|Platyhelminthes	0	0	0	0	0	0	0	0	0	279.31
OTU153	Metazoa|Annelida|Phyllodocida|Phyllodocidae	0	0	0	0	0	0	0	0	0	0
OTU154	Metazoa|Annelida|Spionida|Cirratulidae	0	0	0	0	0	0	0	0	0	0
OTU155	Metazoa|Annelida|Spionida|Cirratulidae	0	0	0	0	0	0	0	0	0	0
OTU156	Metazoa|Arthropoda|Crustacea|Maxillopoda|Copepoda	0	0	0	0	0	0	0	0	0	0
OTU157	Metazoa|Annelida|Phyllodocida|Phyllodocidae	0	0	0	0	0	0	0	0	0	0
OTU158	Metazoa|Annelida|Phyllodocida|Phyllodocidae	0	0	0	0	0	0	0	0	0	0
OTU159	Metazoa|Rotifera	0	0	0	0	0	0	0	0	0	0
OTU160	Metazoa|Arthropoda|Crustacea|Maxillopoda|Copepoda	0	0	0	0	0	0	0	0	0	0
OTU161	Metazoa|Platyhelminthes	0	0	0	0	0	0	0	0	0	0
OTU162	Metazoa|Annelida|Capitellida|Capitellidae|Capitella	0	0	0	0	0	0	0	0	0	0
OTU163	Metazoa|Nematoda	0	0	0	0	0	0	0	0	0	0
OTU164	Metazoa|Nematoda	0	0	0	0	0	0	0	0	0	0
OTU165	Metazoa|Nematoda	0	0	0	0	0	0	0	0	0	0
OTU167	Metazoa|Nematoda	0	0	0	0	0	0	0	0	0	0
OTU168	Metazoa|Cnidaria|Cnidaria_X|Hydrozoa	0	0	0	0	0	0	0	0	0	0
OTU169	Metazoa|Arthropoda|Crustacea|Maxillopoda|Copepoda	0	0	0	0	0	0	0	0	0	0

OTU170	Metazoa|Gastrotricha	0	0	0	0	0	0	0	0	0	0
OTU171	Metazoa|Annelida|Phyllodocida|Syllidae	0	0	0	0	0	0	0	0	0	0
OTU173	Metazoa|Nematoda	0	0	0	0	0	0	0	0	0	0
OTU174	Metazoa|Arthropoda|Crustacea|Maxillopoda|Copepoda	0	0	0	0	0	0	0	0	0	0
OTU175	Metazoa|Arthropoda|Crustacea|Maxillopoda|Copepoda	0	0	0	0	0	0	0	0	0	0
OTU176	Metazoa|Platyhelminthes	0	0	0	0	0	0	0	0	0	0
OTU178	Metazoa|Nematoda	0	0	0	0	0	0	0	0	0	0
OTU180	Metazoa|Nematoda	0	0	0	0	0	0	0	0	0	0
OTU183	Metazoa|Annelida|Oligochaeta|Tubificidae	0	0	0	0	0	0	0	0	0	0
OTU184	Metazoa|Nematoda	0	0	0	0	0	0	0	0	0	0
OTU186	Metazoa|Platyhelminthes	0	0	0	0	0	0	0	0	0	0
OTU188	Metazoa|Annelida|Terebellida|Ampharetidae	0	0	0	0	0	0	0	0	0	0
OTU190	Metazoa|Annelida|Terebellida|Terebellidae	0	0	0	0	0	0	0	0	0	0
OTU191	Metazoa|Annelida|Phyllodocida|Syllidae	0	0	0	0	0	0	0	0	0	0
OTU192	Metazoa|Annelida|Spionida|Cirratulidae	0	0	0	0	0	0	0	0	0	0

Supplementary Table 7. continued

OTU	assign	DNA_10_1	DNA_10_2	DNA_10_3	DNA_10_4	DNA_10_5
OTU1	Metazoa|Annelida|Capitellida|Capitellidae|Capitella	61.25	0	0	0	0
OTU2	Metazoa|Annelida|Oligochaeta|Tubificidae	0	0	0	0	0
OTU4	Metazoa|Annelida|Phyllodocida|Phyllodocidae|Phyllodoce|Phyllodoce+maculata	0	0	0	0	0
OTU5	Metazoa|Annelida|Spionida|Spionidae|Malacoceros|Malacoceros+fuliginosus	0	0	0	0	0
OTU6	Metazoa|Annelida|Spionida|Cirratulidae	0	0	182.01	0	0
OTU7	Metazoa|Platyhelminthes	0	546.06	0	0	0
OTU8	Metazoa|Annelida|Phyllodocida|Syllidae|Salvatoria	0	0	0	0	0
OTU9	Metazoa|Annelida	2524.68	0	0	0	0
OTU10	Metazoa|Nematoda	0	0	0	0	0
OTU11	Metazoa|Nematoda	0	0	0	0	0
OTU12	Metazoa|Nematoda	0	0	0	0	0
OTU13	Metazoa|Cnidaria|Cnidaria_X|Anthozoa	0	0	0	0	0
OTU14	Metazoa|Nemertea	0	0	0	0	0
OTU15	Metazoa|Mollusca|Bivalvia|Heteroconchia|Abra	0	0	0	0	0
OTU16	Metazoa|Platyhelminthes	0	1263.45	3680.48	1615.87	240.92
OTU17	Metazoa|Cnidaria|Cnidaria_X|Hydrozoa	0	716.09	0	2110.86	0
OTU18	Metazoa|Platyhelminthes	0	0	0	0	0
OTU19	Metazoa|Arthropoda|Crustacea|Ostracoda	0	0	0	0	0
OTU20	Metazoa|Nematoda	0	0	0	0	0
OTU21	Metazoa|Annelida|Terebellida|Ampharetidae	0	0	0	0	0
OTU22	Metazoa|Annelida|Phyllodocida|Syllidae|Exogone|Exogone+naidina	0	0	0	0	0
OTU23	Metazoa|Platyhelminthes	0	0	0	0	0
OTU24	Metazoa|Platyhelminthes	0	0	0	0	0
OTU25	Metazoa|Mollusca|Bivalvia|Heteroconchia|Corbula	0	0	0	0	3751.12
OTU26	Metazoa|Annelida|Capitellida|Capitellidae|Heteromastus	102.59	0	0	0	0

OTU27	Metazoa|Arthropoda|Crustacea|Maxillopoda|Copepoda	0	0	0	0	0
OTU28	Metazoa|Nematoda	0	0	0	0	0
OTU29	Metazoa|Cnidaria|Cnidaria_X|Hydrozoa	0	0	0	0	0
OTU30	Metazoa|Mollusca|Gastropoda|Heterobranchia|Bulla	41.42	0	0	0	0
OTU31	Metazoa|Platyhelminthes	0	0	0	0	0
OTU32	Metazoa|Arthropoda|Crustacea|Ostracoda	0	0	0	0	0
OTU33	Metazoa|Nemertea	0	0	0	0	0
OTU35	Metazoa|Nemertea	0	1029.42	0	0	0
OTU36	Metazoa|Arthropoda|Chelicerata	0	0	0	0	0
OTU37	Metazoa|Annelida|Phyllodocida|Syllidae|Erinaceusyllis|Erinaceusyllis+cryptica	0	0	0	0	0
OTU38	Metazoa|Platyhelminthes	0	0	0	0	0
OTU39	Metazoa|Arthropoda|Crustacea|Maxillopoda|Copepoda	0	0	0	0	0
OTU40	Metazoa|Annelida|Capitellida|Maldanidae|Praxillella	0	0	0	0	0
OTU41	Metazoa|Arthropoda|Crustacea|Maxillopoda|Copepoda	0	0	0	0	0
OTU42	Metazoa|Annelida|Phyllodocida|Syllidae|Exogone|Exogone+naidina	0	0	0	0	0
OTU43	Metazoa|Hemichordata	0	0	0	0	0
OTU44	Metazoa|Cnidaria|Cnidaria_X|Hydrozoa	672.97	0	0	0	0
OTU45	Metazoa|Mollusca|Bivalvia|Protobranchia|Nucula	0	0	0	0	0
OTU46	Metazoa|Annelida|Capitellida|Capitellidae|Capitella	0	0	0	0	0
OTU47	Metazoa|Platyhelminthes	0	0	0	0	0
OTU48	Metazoa|Platyhelminthes	0	0	0	0	0
OTU49	Metazoa|Platyhelminthes	699.09	0	0	0	0
OTU51	Metazoa|Nematoda	0	0	0	0	0
OTU52	Metazoa|Annelida|Capitellida|Maldanidae	0	418.97	0	0	0
OTU53	Metazoa|Nematoda	0	0	0	0	0
OTU54	Metazoa|Annelida|Oligochaeta|Tubificidae	0	0	0	0	0
OTU55	Metazoa|Platyhelminthes	0	0	0	0	0
OTU56	Metazoa|Platyhelminthes	0	0	0	0	0
OTU58	Metazoa|Nematoda	0	0	0	0	0

OTU59	Metazoa|Echinodermata|Echinodermata_X|Echinodermata_XX|Amphiura	0	0	0	0	0
OTU60	Metazoa|Platyhelminthes	0	0	0	0	0
OTU61	Metazoa|Cnidaria|Cnidaria_X|Hydrozoa	0	0	0	0	95.01
OTU62	Metazoa|Platyhelminthes	0	0	0	0	0
OTU64	Metazoa|Arthropoda|Crustacea|Maxillopoda|Copepoda	0	0	0	0	0
OTU65	Metazoa|Nematoda	0	0	0	0	0
OTU66	Metazoa|Arthropoda|Crustacea|Maxillopoda|Copepoda	0	0	0	0	0
OTU68	Metazoa|Platyhelminthes	0	53.54	0	0	0
OTU69	Metazoa|Platyhelminthes	0	0	0	0	0
OTU70	Metazoa|Arthropoda|Crustacea|Maxillopoda|Copepoda	0	0	0	0	0
OTU71	Metazoa|Platyhelminthes	0	0	0	0	0
OTU72	Metazoa|Nematoda	0	0	0	0	0
OTU73	Metazoa|Nematoda	0	21.3	0	0	0
OTU75	Metazoa|Gastrotricha	0	0	120.06	375.27	0
OTU76	Metazoa|Arthropoda|Crustacea|Maxillopoda|Copepoda	0	0	0	0	0
OTU78	Metazoa|Mollusca|Gastropoda|Heterobranchia|Calliopaea|Calliopaea+bellula	0	0	0	0	0
OTU79	Metazoa|Annelida|Phyllodocida|Syllidae	0	0	0	0	0
OTU80	Metazoa|Echinodermata|Echinodermata_X|Echinodermata_XX|Leptosynapta	0	0	0	0	0
OTU82	Metazoa|Annelida|Capitellida|Capitellidae|Capitella	0	0	0	0	0
OTU84	Metazoa|Annelida|Capitellida|Capitellidae|Capitella	0	0	0	0	0
OTU85	Metazoa|Platyhelminthes	0	0	0	0	0
OTU86	Metazoa|Platyhelminthes	0	0	0	0	0
OTU87	Metazoa|Annelida|Terebellida|Terebellidae	0	0	0	0	0
OTU88	Metazoa|Platyhelminthes	0	0	0	0	0
OTU89	Metazoa|Annelida|Oligochaeta|Tubificidae	0	0	0	0	0
OTU90	Metazoa|Cnidaria|Cnidaria_X|Anthozoa|Ceriantheopsis	0	0	0	0	0
OTU91	Metazoa|Annelida|Capitellida|Scalibregmatidae|Scalibregma|Scalibregma+inflatum	0	53.17	0	0	0
OTU92	Metazoa|Annelida|Scolecida|Opheliidae|Ophelina	0	0	0	0	0
OTU93	Metazoa|Gastrotricha	0	0	0	0	0

OTU94	Metazoa|Platyhelminthes	0	0	0	0	0
OTU95	Metazoa|Arthropoda|Crustacea|Ostracoda	0	0	0	0	0
OTU96	Metazoa|Platyhelminthes	0	0	0	0	0
OTU97	Metazoa|Platyhelminthes	0	0	0	0	0
OTU98	Metazoa|Platyhelminthes	0	0	0	0	0
OTU99	Metazoa|Platyhelminthes	0	0	0	0	0
OTU100	Metazoa|Arthropoda|Crustacea|Ostracoda	0	0	0	0	0
OTU101	Metazoa|Annelida|Phyllodocida|Phyllodocidae	0	0	0	0	0
OTU102	Metazoa|Arthropoda|Crustacea|Maxillopoda|Copepoda	0	0	0	0	0
OTU103	Metazoa|Nemertea	0	0	0	0	0
OTU104	Metazoa|Annelida|Phyllodocida|Syllidae	0	0	0	0	0
OTU107	Metazoa|Arthropoda|Crustacea|Ostracoda	0	0	0	0	0
OTU108	Metazoa|Annelida|Spionida|Spionidae|Malacoceros|Malacoceros+fuliginosus	0	0	0	0	0
OTU109	Metazoa|Arthropoda|Crustacea|Maxillopoda|Copepoda	0	0	0	0	0
OTU110	Metazoa|Arthropoda|Crustacea|Maxillopoda|Copepoda	0	0	0	0	0
OTU111	Metazoa|Kinorhyncha	0	0	0	0	0
OTU112	Metazoa|Platyhelminthes	0	0	0	0	0
OTU113	Metazoa|Nematoda	0	0	0	0	0
OTU114	Metazoa|Nematoda	0	0	0	0	0
OTU115	Metazoa	0	0	0	0	14.95
OTU116	Metazoa|Platyhelminthes	0	0	0	0	0
OTU117	Metazoa|Nematoda	0	0	0	0	0
OTU118	Metazoa|Arthropoda|Crustacea|Maxillopoda|Copepoda	0	0	0	0	0
OTU119	Metazoa|Nematoda	0	0	0	0	0
OTU120	Metazoa|Platyhelminthes	0	0	0	0	0
OTU121	Metazoa|Nematoda	0	0	0	0	0
OTU122	Metazoa|Annelida|Oligochaeta|Tubificidae	0	0	0	0	0
OTU123	Metazoa|Mollusca|Bivalvia|Heteroconchia|Myrtea|Myrtea+spinifera	0	0	0	0	0
OTU124	Metazoa|Annelida|Terebellida|Pectinariidae|Pectinaria	0	0	0	0	0

OTU125	Metazoa|Platyhelminthes	0	0	0	0	0
OTU126	Metazoa|Nematoda	0	0	0	0	0
OTU128	Metazoa|Kinorhyncha	0	0	0	0	0
OTU130	Metazoa|Annelida|Phyllodocida|Phyllodocidae	0	0	0	0	0
OTU131	Metazoa|Annelida|Oligochaeta|Tubificidae	0	0	0	0	0
OTU132	Metazoa|Nematoda	0	0	0	0	0
OTU134	Metazoa|Annelida|Phyllodocida|Syllidae	0	0	0	0	0
OTU135	Metazoa|Annelida|Capitellida|Capitellidae|Capitella	0	0	0	0	0
OTU136	Metazoa|Nematoda	0	0	0	0	0
OTU137	Metazoa|Hemichordata	0	0	0	0	0
OTU138	Metazoa	0	0	0	0	0
OTU141	Metazoa|Nematoda	0	0	0	0	0
OTU143	Metazoa|Annelida|Capitellida|Capitellidae|Capitella	0	0	0	0	0
OTU144	Metazoa|Platyhelminthes	0	0	0	0	0
OTU145	Metazoa|Annelida|Spionida|Spionidae|Malacoceros|Malacoceros+fuliginosus	0	0	0	0	0
OTU146	Metazoa|Arthropoda|Crustacea|Maxillopoda|Copepoda	0	0	0	0	0
OTU147	Metazoa|Porifera	0	0	0	0	0
OTU149	Metazoa|Mollusca|Bivalvia|Heteroconchia|Macoma|Macoma+nasuta	0	0	0	0	0
OTU151	Metazoa|Nematoda	0	0	0	0	0
OTU152	Metazoa|Platyhelminthes	0	0	0	0	0
OTU153	Metazoa|Annelida|Phyllodocida|Phyllodocidae	0	0	0	0	0
OTU154	Metazoa|Annelida|Spionida|Cirratulidae	0	0	0	0	0
OTU155	Metazoa|Annelida|Spionida|Cirratulidae	0	0	0	0	0
OTU156	Metazoa|Arthropoda|Crustacea|Maxillopoda|Copepoda	0	0	0	0	0
OTU157	Metazoa|Annelida|Phyllodocida|Phyllodocidae	0	0	0	0	0
OTU158	Metazoa|Annelida|Phyllodocida|Phyllodocidae	0	0	0	0	0
OTU159	Metazoa|Rotifera	0	0	0	0	0
OTU160	Metazoa|Arthropoda|Crustacea|Maxillopoda|Copepoda	0	0	0	0	0
OTU161	Metazoa|Platyhelminthes	0	0	0	0	0

OTU162	Metazoa|Annelida|Capitellida|Capitellidae|Capitella	0	0	0	0	0
OTU163	Metazoa|Nematoda	0	0	0	0	0
OTU164	Metazoa|Nematoda	0	0	0	0	0
OTU165	Metazoa|Nematoda	0	0	0	0	0
OTU167	Metazoa|Nematoda	0	0	0	0	0
OTU168	Metazoa|Cnidaria|Cnidaria_X|Hydrozoa	0	0	0	0	0
OTU169	Metazoa|Arthropoda|Crustacea|Maxillopoda|Copepoda	0	0	0	0	0
OTU170	Metazoa|Gastrotricha	0	0	0	0	0
OTU171	Metazoa|Annelida|Phyllodocida|Syllidae	0	0	0	0	0
OTU173	Metazoa|Nematoda	0	0	0	0	0
OTU174	Metazoa|Arthropoda|Crustacea|Maxillopoda|Copepoda	0	0	0	0	0
OTU175	Metazoa|Arthropoda|Crustacea|Maxillopoda|Copepoda	0	0	0	0	0
OTU176	Metazoa|Platyhelminthes	0	0	0	0	0
OTU178	Metazoa|Nematoda	0	0	0	0	0
OTU180	Metazoa|Nematoda	0	0	0	0	0
OTU183	Metazoa|Annelida|Oligochaeta|Tubificidae	0	0	0	0	0
OTU184	Metazoa|Nematoda	0	0	119.45	0	0
OTU186	Metazoa|Platyhelminthes	0	0	0	0	0
OTU188	Metazoa|Annelida|Terebellida|Ampharetidae	0	0	0	0	0
OTU190	Metazoa|Annelida|Terebellida|Terebellidae	0	0	0	0	0
OTU191	Metazoa|Annelida|Phyllodocida|Syllidae	0	0	0	0	0
OTU192	Metazoa|Annelida|Spionida|Cirratulidae	0	0	0	0	0
